# Synthesis of a Novel Cysteine-Incorporated Anthraquinone Derivative and Its Structural Properties

**DOI:** 10.3390/molecules200610192

**Published:** 2015-06-03

**Authors:** Akihiro Nomoto, Toshihide Taniguchi, Yuta Minatobe, Syouhei Katao, Kiyomi Kakiuchi, Shigenobu Yano, Akiya Ogawa

**Affiliations:** 1 Department of Applied Chemistry, Graduate School of Engineering, Osaka Prefecture University, 1-1 Gakuen-cho, Nakaku, Sakai, Osaka 599-8531, Japan; E-Mails: tttoshihide1@yahoo.co.jp (T.T.); yuta_minatobe@shokubai.co.jp (Y.M.); 2 Graduate School of Materials Science, Nara Institute of Science and Technology (NAIST), 8916-5, Takayama, Ikoma, Nara 630-0192, Japan; E-Mails: katao@ms.naist.jp (S.K.); kakiuchi@ms.naist.jp (K.K.); yano-shigenobu@ms.naist.jp (S.Y.); 3 Seika Corporation, 1-1-82 Kozaika, Wakayama 641-0007, Japan

**Keywords:** cysteine, anthraquinone, mercapto group, X-ray crystal analysis, packing structure, π-π interaction, artificial molecules

## Abstract

A novel cysteine-incorporated anthraquinone derivative was synthesized, and its molecular structure was determined by X-ray crystal analysis. Each mercapto group was located separately and did not form a disulfide bond, and hydrogen bondings and π-π interaction were observed from the packing structure.

## 1. Introduction

In the field of artificial molecular modeling, cysteine is an attractive compound because of its unique properties. In living organisms, cysteine plays important roles such as protein folding or antioxidant activity depending on the sulfur moiety it contains [1,2,3]. The protein morphology related to cysteine can change drastically because of the formation of a disulfide bond by oxidation. Therefore, the construction of artificial molecules containing mercapto moieties is difficult, because a multi-step synthes is required owing to the reactivities of the mercapto group. For example, in the case of two adjacent cysteine residues, a disulfide bond is easily formed by oxidation, and occasionally, enzymes work well by exploiting such reactivities of cysteine. 

In recent times, anthraquinone has been focused on as a functional molecule in photo- or electrochemical moieties, and also has a rigid planar structure at the molecular level [4,5,6,7,8,9,10,11]. Such properties of anthraquinone have been exploited to synthesize functional ligands as metal receptors [12,13,14,15]. Although bioconjugated molecules containing cysteine and some molecular structures have been reported [16,17,18,19], synthetic examples of anthraquinone derivatives bearing amino acids are still rare [20]. Therefore, in order to realize the incorporation of cysteine residues into anthraquinone, amino acid-containing functional molecules have been investigated. 

From these viewpoints, the construction of cysteine-containing artificial molecules is important for opening up new possibilities for elucidating the functionalities of cysteine. Herein, we report the synthesis and structural properties of a novel cysteine-incorporated anthraquinone derivative.

## 2. Results and Discussion

**Scheme 1:**
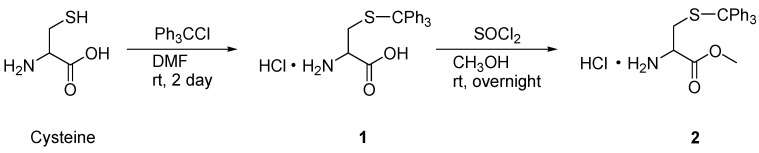
Preparation of *S*-trityl-l-cysteine methyl ester **2**.

According to the reported method [21], cysteine was converted to *S*-trityl-L-cysteine methyl ester (**2**) as shown in Scheme 1. At first, the mercapto group of cysteine was protected by a trityl group. *S*-Trityl-L-cysteine methyl ester (**2**) was prepared by esterification by using SOCl_2_.

**Scheme 2:**
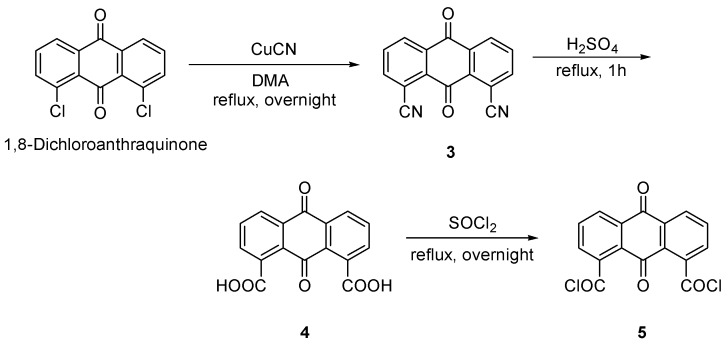
Preparation of anthraquinone-1,8-dicarbonyl dichloride **5**.

Anthraquinone-1,8-dicarbonyl dichloride (**5**) was prepared from 1,8-dichloroanthraquinone in three steps (Scheme 2). Anthraquinone-1,8-dicarboxylic acid (**4**) was prepared [22], followed by chlorination with SOCl_2_ [20]. 

**Scheme 3:**
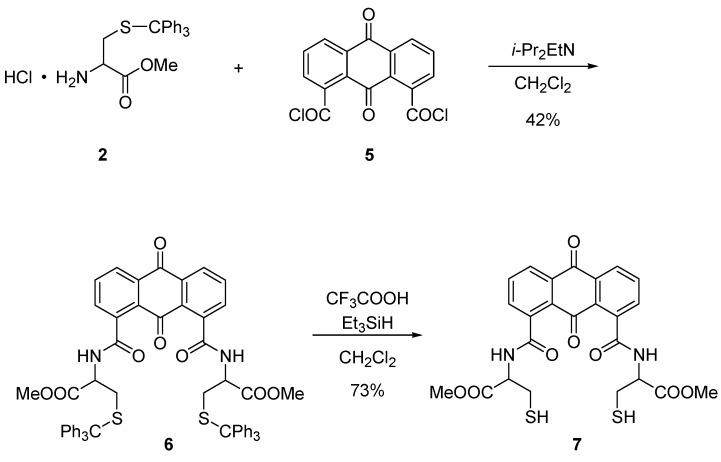
Synthetic route for preparing cysteine-incorporated anthraquinone **7**.

After the coupling reaction of **2** and **5**, the reaction mixture was subjected to silica-gel column chromatography to afford anthraquinone bearing *S*-trityl-L-cysteine methyl esters **6**. Deprotection was conducted using triethylsilane and trifluoroacetic acid, and product anthraquinone bearing L-cysteine methyl esters **7** was also purified by silica-gel column chromatography (Scheme 3). From the ^1^H- and ^13^C-NMR, IR, and mass spectral measurements, **7** was synthesized successfully. 

**Scheme 4:**
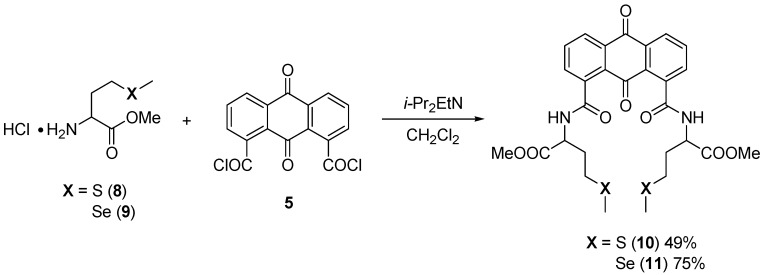
Synthesis of methionine- and selenomethionine-incorporated anthraquinones.

To apply this synthetic protocol to other chalcogen-containing amino acids, L-methionine methyl ester and L-selenomethionine methyl ester were also introduced into the anthraquinone system, successfully affording methionine-anthraquinone (**8**) and selenomethionine-anthraquinone (**9**), respectively (Scheme 4). L-Methionine methyl ester hydrochloride and L-selenomethionine methyl ester hydrochloride were prepared in the same manner as that of *S*-trityl-L-cysteine (shown in Scheme 1) and treated with **5**. After purification of the respective reaction mixtures by column chromatography, the corresponding anthraquinone bearing L-methionine methyl esters **10** and anthraquinone bearing L-selenomethionine methyl esters **11** were obtained. We also attempted to apply this protocol to selenocysteine-incorporated anthraquinone, however, the corresponding trityl-group-protected selenocysteine could not be obtained from commercially available L-selenocystine (Se(Cys)_2_).

**Figure 1:**
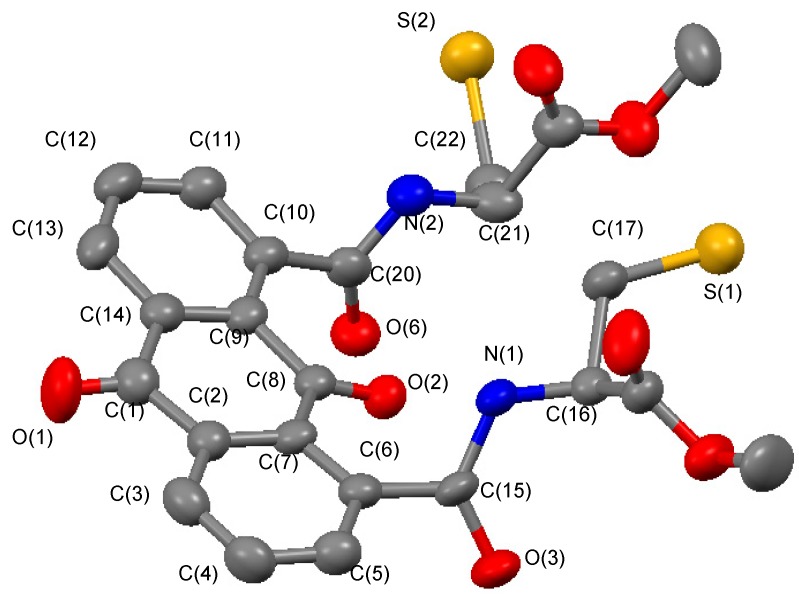
ORTEP drawing of **7** with ellipsoids at 50% probability. Hydrogen atoms are omitted for clarity. Blue, red, yellow, and black atoms indicate N, O, S, and C, respectively.

To clarify the molecular structure, recrystallization of each anthraquinone derivatives was carried out and a single crystal was successfully obtained by recrystallization of compound **7** from acetonitrile. The molecular structure as determined by X-ray crystal analysis is shown in Figure 1. 

The two cysteine chains of **7** were oriented in a similar manner, and the two carbonyl groups at the 1,8-positions were incorporated perpendicularly to the anthraquinone rings with torsion angles of 61.09° and 83.04°, respectively [23,24,25,26,27,28,29]. About the anthraquinone moiety, in contrast to the reported structure, both benzene rings were twisted with a torsion angle of 8.25°. Each mercapto group was located individually, and the distance between them was 7.205 Å in the molecule. Table 1 summarizes the selected bond lengths and angles.

**Table 1:** Selected bond lengths (Å) and bond angles (°).

Bond Lengths (Å)		Bond Angles (°)	
S1–C17	1.8098(19)	S1–C17–C16	114.01(13)
S2–C22	1.810(2)	S2–C22–C21	114.27(13)
O1–C1	1.217(2)	C20–N2–C21	120.32(15)
O2–C8	1.221(2)	C15–N1–C16	121.67(15)
O3–C15	1.229(2)	N1–C15–C6	116.48(15)
O6–C20	1.228(2)	N2–C20–C10	116.00(15)
N1–C15	1.339(2)	C9–C10–C20	122.98(16)
N2–C20	1.346(2)	C7–C6–C15	125.68(15)
C6–C15	1.504(3)	N2–C21–C22	113.14(15)
C10–C20	1.511(2)	N1–C16–C17	108.90(14)
C16–C17	1.524(3)		
C21–C22	1.527(2)		

**Figure 2:**
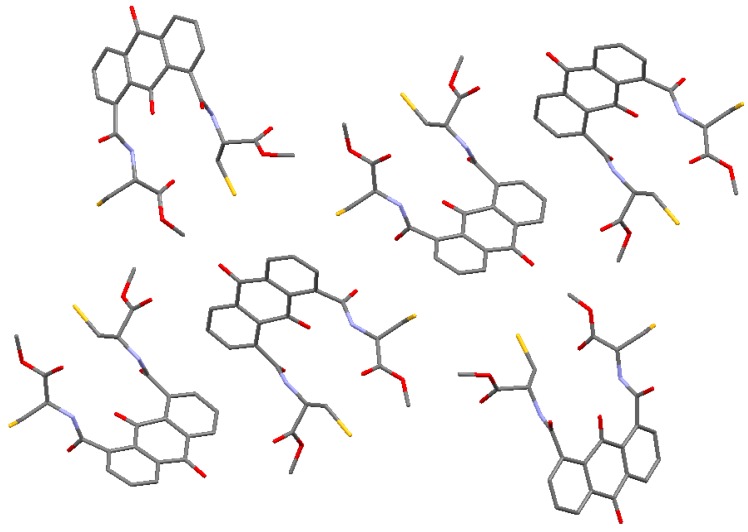
Packing diagram of the unit cell along the *a* axis. Hydrogen atoms are omitted for clarity.

**Figure 3:**
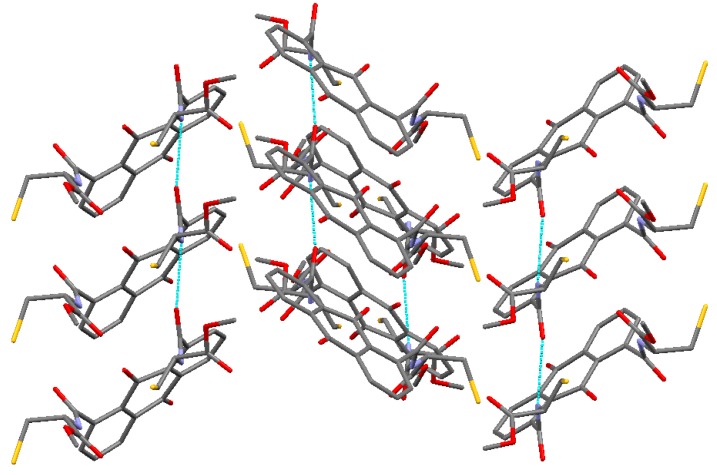
Packing diagram of the unit cell along the *c* axis. Hydrogen atoms are omitted for clarity. Intermolecular hydrogen bondings are indicated by dotted blue lines.

By contrast, intermolecular interactions were also clearly observed. Figure 2 and Figure 3 show the packing diagrams along the *a* and *c* axies, respectively. Anthraquinone moieties were stacked over each other by π-π interactions, and the distance between the planes formed by the middle ring of the anthraquinone moiety calculated using C(1)–C(2)–C(7)–C(8)–C(9)–C(14) atoms is 3.593 Å (Table 2). According to reported crystal structure of anthraquinone derivatives bearing dicarbonyl groups, anthraquinone-2,3-dicarboxylic acid (abbreviated as H_2_AQDC) coordinates with the metal ion species to afford metal complexes [25]. As compared to the π-π interactions of CaAQDC and ZnAQDC with that of compound **7**, distances of π-π interaction distances are 3.04 Å (Ca complex) and 3.56 Å (Zn complex) calculated by PLATON (Table 3) [11]. In the crystal of **7**, hydrogen bonding was observed between the carbonyl group incorporated at C6 and the peptide amine, and the selected distances and angles were 0.88 Å (N-H), 2.08 Å (N-H ∙∙∙ O=C), and 2.89 Å and 153° (N-H ∙∙∙ O) (Table 4) [30]. 

**Table 2:** π-π interactions (Å, °).

*Cg*(*I*)	*Cg*(*J*)	*Cg*–*Cg*	Alpha	*CgI*_Perp	*CgJ*_Perp
*Cg*(1)	*Cg*(2) ^(i)^	3.9249(12)	4.45(9)	3.7289(8)	3.6403(8)
*Cg*(3)	*Cg*(1) ^(i)^	3.7590(11)	0.8900	3.4720(8)	3.3207(8)
*Cg*(3)	*Cg*(2) ^(i)^	4.0899(11)	0.0355	3.5948(8)	3.5693(8)

*Cg*(1), *Cg*(2) and *Cg*(3) are the centroids of the C(1)–C(2)–C(7)–C(8)–C(9)–C(14), C(2)–C(3)–C(4)–C(5)–C(6)–C(7) and C(9)–C(10)–C(11)–C(12)–C(13)–C(14) rings, respectively. *Cg*–*Cg* = distance between ring centroids; Alpha = dihedral angle between planes *I* and *J*; *CgI*_Perp = perpendicular distance of *Cg*(*I*) on ring *J*; *CgJ*_Perp = perpendicular distance of *Cg*(*J*) on ring *I*; Slippage = distance between *Cg*(*I*) and perpendicular projection of *Cg*(*J*) on ring *I*.: 3.498 Å (*Cg*(1)–*Cg*(1)), 3.393 Å (*Cg*(2)–*Cg*(2)), and 3.829 Å (*Cg*(3)–*Cg*(3)). Symmetry codes: (i) −1 + X, Y, Z.

**Table 3:** Comparison of π-π interactions (Å).

Compounds	*Cg*–*Cg*	*CgI*_Perp	Slippage
7	– *^a^*	3.5932(8)	– *^b^*
Ca complex	– *^a^*	3.0382(13)	– *^b^*
Zn complex	4.063(4)	3.557(3)	1.964

*Cg*–*Cg* = distance between the central rings of anthraquinone: *^a^* 5.0147(12) Å (**7**): 5.960(3) Å (Ca complex). Slippage = distance between the central rings of anthraquinone: *^b^* 3.498 Å (**7**): 5.127 Å (Ca complex).

**Table 4:** Hydrogen-bond geometry (Å, °).

*D*–H ••• *A*	*D*–H	H ••• *A*	*D* ••• *A*	*D*–H ••• *A*
N1–H3 ••• O3 ^(i)^	0.88	2.08	2.890(2)	153

Symmetry codes: (i) −1 + X, Y, Z.

The molecules are linked into an infinite chain along the *a*-axis by hydrogen bonds, and mercapto groups were free from forming hydrogen bondings. Intra- or intermolecular disulfide bonds (-S-S-) were not formed in the crystal, and this was also supported by a ^1^H-NMR measurement in solution. From these results, this molecule may act as metal receptor for recognizing metal ions coordinated by mercapto groups between two cysteine strands.

**Figure 4:**
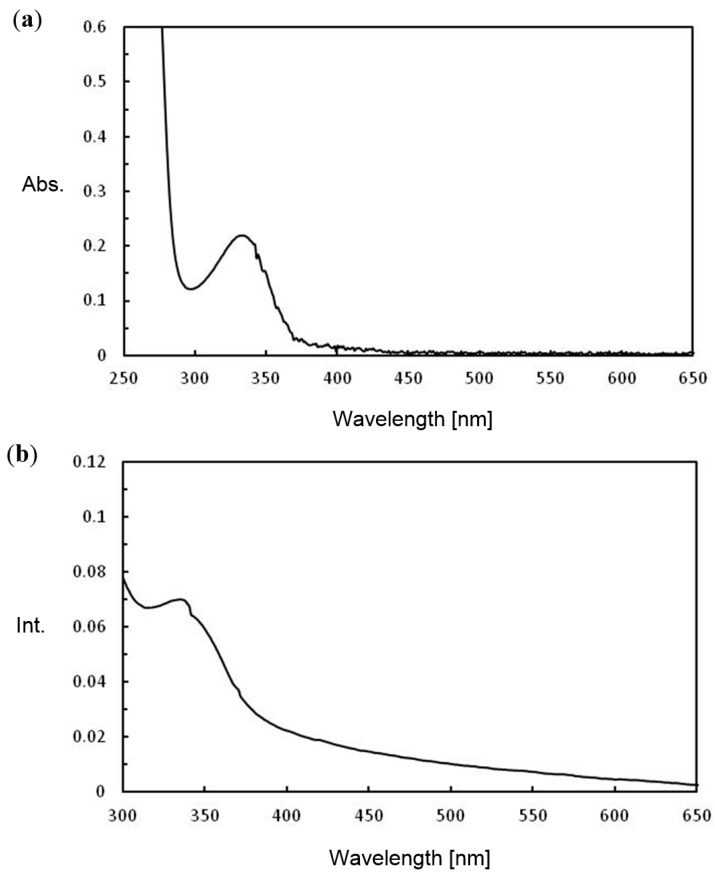
UV-vis. spectra of **7** (**a**) in acetonitrile (2.0 × 10^−^^3^ M); (**b**) casted onto Pyrex slide glass.

The photochemical properties were elucidated from the UV-vis and fluorescence spectra. Compound **7** was dissolved in acetonitrile (2.0 × 10^−3^ M) and measurements were carried out (Figure 4a). In the UV-vis spectrum, an absorbance peak was observed at 335 nm (ε = 110) [31]. The solid-state spectrum was also measured by depositing onto Pyrex glass (Figure 4b). Although a broadened peak was observed around 400–500 nm, there was no shift in the absorbance peak, thus each amide group is oriented in a perpendicular manner. The fluorescence excited at 350 nm appeared at 400–600 nm in Supplementary Materials (Fluorescence spectra of **7**), which corresponds to the anthraquinone moiety as reported [23,24,25,26].

## 3. Experimental Section 

### 3.1. General

All chemicals were purchased from Aldrich (St. Louis, MO, USA), Wako (Osaka, Japan), TCI (Tokyo, Japan), and Nacalai (Kyoto, Japan). The starting materials **2** and **5** were prepared by modifying the reported procedures as described below. All reagents and solvents were used without further purification. ^1^H-NMR spectra were recorded on a JNM-AL 400 (400 MHz) spectrometer (JEOL, Tokyo, Japan) using CDCl_3_ as the solvent with Me_4_Si as the internal standard. ^13^C-NMR spectra were taken on a JEOL JNM-GSX-400 (100 MHz) spectrometer using CDCl_3_ as the solvent. The purification of the products were carried out by MPLC (silica gel, 25–40 µm, length 310 mm, i.d. 25 mm), preparative TLC (PTLC) on Wakogel B-5F silica gel, or using a recycling preparative HPLC (Model LC-908, Japan Analytical Industry Co. Ltd., Tokyo, Japan) equipped with JAIGEL-1H and -2H columns (GPC) using CHCl_3_ as an eluent. IR, HR-mass spectra, UV-vis. and fluorescence spectra were determined on a JEOL JMS-700, FT/IR-8900 Fourier Transform Infrared Microsampling System (JASCO, Tokyo, Japan), JASCO V-560 and JASCO FP-6300, respectively. X-ray crystallographic measurements were carried out on a RAXIS-RAPID diffractometer (Rigaku, Tokyo, Japan) with Mo-Kα radiation. The resulting report and PRATON files are in Supplementary Materials.

### 3.2. Synthesis of S-Trityl-l-cysteine (**1**)

L-Cysteine hydrochloride (10.0 g, 63.4 mmol) and trityl chloride (27.0 g, 96.9 mmol) were stirred in DMF (40 mL) for 2 days at room temperature. A 10% sodium acetate solution (350 mL) was then added, and the precipitate was filtered and washed with distilled water. Afterward, the residue was stirred in acetone at 50 °C for 30 min and filtered after cooling. The residue was washed with little acetone and diethyl ether. After drying *in vacuo*, 20.5 g (89%) of **1** was obtained as a white powder: mp 195 °C (dec); ^1^H-NMR (DMSO-*d_6_*) δ 2.34–2.39 (m, 1H), 2.52–2.56 (m, 1H), 2.86–2.89 (m, 1H), 7.27–7.37 (m, 15H).

### 3.3. Synthesis of S-Trityl-l-cysteine Methyl Ester (**2**)

*S*-Trityl-l-cysteine (3.2 g, 8.0 mmol) was added to methanol (70 mL) to form a suspension. After cooling to 0–5 °C, thionyl chloride (23 mL, 320 mmol) was added, and the reaction mixture was allowed to warm for 20 h at room temperature. The mixture was then refluxed for 5 h, cooled, and evaporated *in vacuo*. After drying *in vacuo*, compound **2** (3.2 g, 98%) was obtained as crystalline solid. ^1^H-NMR (DMSO-*d_6_*) δ 2.46–2.64 (m, 2H), 3.67 (s, 3H), 3.72–3.78 (m, 1H), 7.24–7.33 (m, 15H).

### 3.4. Synthesis of 1,8-Dicyanoanthraquinone (**3**)

1,8-Dichloroanthraquinone (1.39 g, 5.0 mmol) and CuCN (1.34 g, 15 mmol) were slurried in DMA (50 mL) and refluxed under N_2_ for 3 h. The hot brown solution was poured onto ice (700 g), and the brown-green precipitate was filtered and washed with water. The copper complex was decomposed with 3 N HNO_3_ (150 mL) at 60 °C for 5 h. The brown solid was filtered, washed with water and air-dried. This procedure afforded crude **3** (1.2 g, 88%).

### 3.5. Synthesis of Anthraquinone-1,8-dicarboxylic Acid (**4**)

1,8-Dicyanoanthraquinone (**3**, 2.4 g, 9.3 mmol) was refluxed in 70% H_2_SO_4_ (500 mL) for 1 h. The hot solution was poured onto ice (500 g) to precipitate crude **4** as a brown solid (2.6 g, 94%).

### 3.6. Synthesis of Anthraquinone-1,8-dicarbonyl Dichloride (**5**)

Anthraquinone-1,8-dicarboxylic acid (**4**, 2.4 g, 9.3 mmol) was refluxed in thionyl chloride (11 mL, 150 mmol) for 20 h. After drying *in vacuo*, compound **5** was obtained as a black solid and used without further purification.

### 3.7. Synthesis of **6**

Anthraquinone-1,8-dicarboxylic acid (1.60 g, 6 mmol) was refluxed in SOCl_2_ (10 mL) in two-necked flask (100 mL) for 6 h. After refluxing, the solution was removed *in vacuo* to give dark-brown solid, anthraquinone-1,8-dicarbonyl chloride. After adding dry CH_2_Cl_2_ (30 mL) and *S*-trt-Cys(OMe) hydrochloride (5.38 g, 13 mmol) into the flask, the solution was stirred at 0 °C in the ice bath under inert atomosphere with dropwise addition of diisopropylethylamine (4.31 mL, 25 mmol). The resulting mixture was allowed to warm to ambient temperature and was stirred overnight. After evaporation of solvents, CHCl_3_ (20 mL) was added and the mixture was extracted with 1 N HCl (20 mL), sat. NaHCO_3_ (20 mL), H_2_O (20 mL) and sat. brine (20 mL). The organic layer was dried by MgSO_4_ and evaporated the CHCl_3_. The resulting brown oil was purified by silica gel column chromatography (Rf = 0.37, eluant hexane/EtOAc = 1:1) to yield 42% of anthraquinone-*S*-trt-Cys(OMe) (yellow solid). ^1^H-NMR (400 MHz, CDCl_3_) δ 2.73–2.84 (m, 4H), 3.73 (s, 6H), 4.67–4.71 (m, 2H), 6.13 (d, 6.88 Hz, 2H), 7.11–7.42 (m, 15H), 7.72–7.81 (m, 4H), 8.36 (dd, 1.36 Hz, 4.56 Hz, 2H); ^13^C-NMR (100 MHz, CDCl_3_) δ 33.7, 52.2, 52.8, 67.0, 126.9, 128.1, 128.7, 129.6, 131.2, 133.4, 133.8, 134.2, 137.7, 144.5, 168.5, 170.6, 181.4, 182.0. 

### 3.8. Synthesis of **7**

Anthraquinone-*S*-trt-Cys(OMe) (2.49 g, 2.53 mmol) was dissolved in CH_2_Cl_2_ (20 mL) in a 100 mL round-bottomed flask, and triethylsilane (1.2 mL, 7.53 mmol) and TFA (19.4 mL, 0.26 mol) were added. The reaction mixture was subsequently stirred at ambient temperature under an inert atmosphere for 30 min. Solvents were removed, and the residue was purified by silica-gel column chromatography (Rf = 0.2, eluant MeOH). Mp 218–220 °C (decomp.); ^1^H-NMR (400 MHz, CDCl_3_) δ 1.90 (t, *J* = 8.7 Hz, 2H), 3.07–3.14 (m, 2H), 3.47–3.55 (m, 2H), 3.82 (s, 6H), 5.12–5.15 (m, 2H), 6.78 (d, *J* = 7.3 Hz, 2H), 7.71 (dd, *J* = 1.4, 7.7 Hz, 2H), 7.82 (t, *J* = 7.3 Hz, 2H), 8.37 (dd, *J* = 1.4, 7.7 Hz, 2H); ^13^C-NMR (100 MHz, CDCl_3_) δ 26.2, 52.9, 54.4, 128.7, 130.3, 133.3, 133.8, 134.2, 137.6, 168.7, 170.4, 181.4, 182.0; IR (KBr, cm^−^^1^) 1541.0, 1637.5, 1683.7, 1739.7 (C=O); HRMass (FAB) Calcd. for C_24_H_23_O_8_N_2_S_2_ (M + H^+^) 531.0896 Found: 531.0897 MR Mass. Calcd for C_24_H_23_O_8_N_2_S_2_; Found (M + H^+^) 531.0896. 

### 3.9. Crystal Structures of **7**

Of the 23,904 reflections were collected, 5463 were unique (Rint = 0.0368); equivalent reflections were merged. The linear absorption coefficient, µ, for Mo-Kα radiation is 2.771 cm^−1^. An empirical absorption correction was applied which resulted in transmission factors ranging from 0.823 to 0.992. The data were corrected for Lorentz and polarization effects. 

The structure was solved by direct methods1 and expanded using Fourier techniques. The non-hydrogen atoms were refined anisotropically. Hydrogen atoms were refined using the riding model.

Crystallographic data: formula weight = 530.57; orthorhombic; space group *P*2_1_2_1_2_1_; *a* = 5.01471(10) Å, *b* = 18.6873(3) Å, *c* = 25.4365(6) Å; *V* = 2383.70(8) Å^3^, *Z* = 4; ρ_calcd_ = 1.478 g cm^−3^; total reflections collected = 23,904; *GOF* = 1.081; *R_1_* = 0.0362; *wR_2_* = 0.0925. Crystallographic data have been deposited with Cambridge Crystallographic Data Centre (CCDC-867403). These data can be obtained free of charge via http://www.ccdc.cam.ac.uk/conts/retrieving.html (or from the CCDC, 12 Union Road, Cambridge CB2 1EZ, UK; Fax: +44 1223 336033; E-mail: deposit@ccdc.cam.ac.uk).

### 3.10. Synthesis of **10**

Anthraquinone-1,8-dicarbonyl chloride (0.24 g, 0.72 mmol) was prepared as described in the synthesis of **7** and dissolved in dry CH_2_Cl_2_ (20 mL) and methionine methylester hydrochloride (0.24 g, 1.44 mmol) was added into the flask, the solution was stirred at 0 °C in the ice bath under an inert atomosphere with dropwise addition of diisopropylethylamine (0.5 mL, 2.88 mmol). The resulting mixture was allowed to warm to ambient temperature and was stirred overnight. After evaporation of solvents, the resulting brown oil was purified by silica gel column chromatography without extraction (Rf = 0.11, eluant hexane/EtOAc = 1:1) to yield 49% of anthraquinone-Met(OMe) (yellow solid). Mp 78–80 °C; ^1^H-NMR (400 MHz, CDCl_3_) δ 2.07 (s, 6H), 2.05–2.25 (m, 4H), 2.58–2.66 (m, 4H), 3.72 (s, 6H), 4.85 (td, *J* = 8.0 Hz, 12.0 Hz, 2H), 7.01 (d, *J* = 8.0 Hz, 2H), 7.54–7.60 (m, 4H), 8.06 (dd, *J* = 4.0 Hz, 8.0 Hz, 2H); ^13^C-NMR (100 MHz, CDCl_3_) δ 15.2, 29.8, 31.1, 52.0, 52.3, 128.1, 130.5, 132.7, 133.6, 133.8, 137.4, 168.6, 172.2, 181.2, 181.5; IR (KBr, cm^−^^1^) 1438.8, 1523.7, 1678.0, 1743.5 (C=O), 3340.5 (NH); Mass (FAB) Calcd. for C_28_H_30_O_8_N_2_S_2_ (M + H^+^) 587. Found: 587.

### 3.11. Synthesis of **11**

Anthraquinone-1,8-dicarbonyl chloride (0.409 g, 1.22 mmol) was prepared as described in the synthesis of **7** and dissolved in dry CH_2_Cl_2_ (30 mL) and selenomethionine methylester hydrochloride (0.606 g, 2.45 mmol) was added into the flask, the solution was stirred at 0 °C in the ice bath under inert atomosphere with dropwise addition of diisopropylethylamine (0.855 mL, 4.88 mmol). The resulting mixture was allowed to warm to ambient temperature and was stirred overnight. After evaporation of solvents, the resulting brown oil was purified by silica gel column chromatography without extraction (Rf = 0.11, eluant hexane/EtOAc = 1:1) to yield 75% of anthraquinone-SeMet(OMe) (yellow solid). Mp 88–90 °C; ^1^H-NMR (400 MHz, CDCl_3_) δ 2.01 (s, 6H), 2.17–2.23 (m, 2H), 2.28–2.37 (m, 2H), 2.62–2.74 (m, 4H), 3.75 (s, 6H), 4.91 (td, *J* = 5.5 Hz, 7.8 Hz, 2H), 6.74 (d, *J* = 7.8 Hz, 2H), 7.60–7.67 (m, 4H), 8.14 (dd, *J* = 6.0 Hz, 9.2 Hz, 2H); ^13^C-NMR (100 MHz, CDCl_3_) δ 4.3, 20.6, 32.7, 52.5, 53.2, 128.5, 130.8, 133.0, 133.9, 134.1, 137.7, 168.7, 172.3, 181.5, 181.8; IR (KBr, cm^−^^1^) 1577.7, 1647.1, 1678.0, 1743.5 (C=O), 3267.2 (NH); HRMass (FAB) Calcd. for C_24_H_22_N_2_O_8_Se_2_ (M + H^+^) 683.0411. Found: 683.0417.

## 4. Conclusions 

In summary, we synthesized novel cysteine-, methionine-, and selenomethionine-incorporated anthraquinone derivatives. A single crystal of cysteine-incorporated anthraquinone was obtained, and its crystal structure was successfully confirmed. Intermolecular hydrogen bonds were observed in the crystal structure of **7**, and the molecules are linked into an infinite chain by hydrogen bonding. Although the results of UV-vis. spectra in solution and in the solid state indicated that the intermolecular interaction does not seem to be so strong, this molecule may act as a metal receptor based on the cysteine moieties, as the mercapto groups are individually detected, without the formation of disulfide and hydrogen bonds. The investigation of the electrochemical and coordination properties of this derivative is currently underway.

## Supplementary Materials

Supplementary materials can be accessed at: http://www.mdpi.com/1420-3049/20/06/10192/s1.
