# Supplementary material for: Synthesis of a Novel Cysteine-Incorporated Anthraquinone Derivative and Its Structural Properties
Source: Molecules. 2015 Jun 3;20(6):10192–204. doi: 10.3390/molecules200610192 (PMC6272162; doi:10.3390/molecules200610192)
Supplement: Supplementary file 1 [file molecules-20-10192-s004.zip › molecules-80292-supplementary-3.pdf]

PLATON(V-271014)-Run for: A-Co P 21 21 21 R = 0.04 TIME: May 04 19:55:19 2015

(C) 1980-2014 A.L.Spek

Crystal Data

Input Cell (Lattice Type: P) - Temp = 123K				Reduced Cell (Acta Cryst.(1976),A32,297-298)		
a =	5.01471(10) Angstrom	alpha =	90 Degree	a = 5.015	alpha = 90.00	V = 2383.7
b =	18.6873(3)	beta =	90	b = 18.687	beta = 90.00	
c =	25.4365(6)	gamma =	90	c = 25.437	gamma = 90.00	

				Niggli Values		
V =	2383.69(8) Cubic-Angstrom	d(100) =	5.0147 Angstrom			
		d(010) =	18.6873	25.147	349.215	647.016
Lambda(MoKa) =	0.71075 Angstrom	d(001) =	25.4365	0.000	0.000	0.000

Orthogonalization Matrices

(See e.g. J.D.Dunitz, Xray Analysis and Structure Determination of Organic Molecules, Cornell Univ. Press, 1979, P236)

```
(XO)   (   5.01471         0          0 ) (X)   ,   (X)   (   0.19941        0          0 ) (XO)     Orthogonal Axes AO, BO and CO
(YO) = (         0  18.68730          0 )*(Y)   ,   (Y) = (         0  0.05351          0 )*(YO)          are defined as:
(ZO)   (         0         0   25.43650 ) (Z)   ,   (Z)   (         0        0    0.03931 ) (ZO)    AO // A, CO // C*, BO // CO X AO
```

Space Group Symmetry

(See e.g. G. Burns & A.M. Glazer, Space Groups for Solid State Scientists, Academic Press, 1990 or Int. Tables A)

Space Group H-M: P212121 Laue: mmm

Space Group Hall: P 2ac 2ab [Schoenflies: D2^4]

Lattice Type: oP, Acentric, Orthorhombic, Multiplicity: 4(4), No: 19

CHIRAL - See P.G. Jones, Acta Cryst. (1986), A42, 57.

Nr	***** Symmetry Operation(s) *****		
1	X ,	Y ,	Z
2	1/2 - X ,	- Y ,	1/2 + Z
3	1/2 + X ,	1/2 - Y ,	- Z
4	- X ,	1/2 + Y ,	1/2 - Z

ADDSYM - CHECK (cf. MISSYM (C): Le Page, Y., J. Appl. Cryst. (1987), 20, 264-269; J. Appl. Cryst. (1988), 21, 983-984)

- ADDSYM Search on ALL NON-H Chemical Types [Max NonFit 20 Perc]
- Number of Input Atoms Included in Search 36 (Unitcell 144)
- Density based on Input Atom Set = 1.478 g.cm-3 - Vol / Non-H atom = 16.6 Ang+3
- The Structure Implies the Following Symmetry Elements Subject to the Criteria:
- Criteria 1.00 Deg (Metric), 0.25 Ang (Rot.), 0.45 Ang (Inv), 0.45 Ang (Transl)

Symm. Elem	Input Cell_Row	Reduced Cell_Row	(Ang) d	Typ	Dot	(Deg) Angle	Perc Fit	AvrDev. MaxDev.	(Ang)	Input Cell x	y	z
2	[1 0 0]	[1 0 0]	5.01	2	1	0	100	0	Through	0	1/4	1/2
1								0	Screw	1/2	0	0
2	[0 1 0]	[0-1 0]	18.69	2	1	0	100	0	Through	1/2	0	3/4
1								0	Screw	0	1/2	0
2	[0 0 1]	[0 0 1]	25.44	2	1	0	100	0	Through	3/4	1/2	0
1								0	Screw	0	0	1/2

T.R.A.N.S.F.O.R.M.A.T.I.O.N M.A.T.R.I.X for CELL and HKL DATA

```
   Reduced->Convent         Input->Reduced       T = Input->Convent:    a' = T a
-------------------------------------------------------------------------------
(    1     0     0 )   (     1     0     0 )   (     1     0     0 )     Det(T)
(    0    -1     0 ) X (     0    -1     0 ) = (     0     1     0 )       =
(    0     0    -1 )   (     0     0    -1 )   (     0     0     1 )     1.000
```

Cell	Lattice	a	b	c	Alpha	Beta	Gamma	Volume	CrystalSystem	Laue
Input	oP	5.015	18.687	25.437	90.00	90.00	90.00	2384	orthorhombic	mmm
Reduced	P	5.015	18.687	25.437	90.00	90.00	90.00	2384		
Convent	oP	5.015	18.687	25.437	90.00	90.00	90.00	2384	orthorhombic	mmm

:: *** No Obvious Extra Crystallographic Symmetry was Detected ***

:: Note: Rerun in EQUAL Atom Type Mode for more Checks

(1:19-Rule Rounded) Coordinates of Unique Residue(s) Identified. Standard Deviations in the Last Digit are in Parentheses.

Site = Site Symmetry; SSN = Site Symmetry Number; SSOF = SHELX Site Occupation Factor (= S.O.F / SSN).
******* Move = Transformation on Input Data: N.IJK (N = SymOp, IJK = Translation) i.e. 1.555 = nomove
SYMBOLS: Type = D/A = Potential Donor or Acceptor atom, D-H = H on Donor atom, MET = Metal.
******* El Type = AK = Alkali Metal, AE = Alkaline Earth, HL = Halogen, AN = Actinide, LN = Lanthanide, TR = Transition Element.
ARU = Asymmetric Residue Unit encoded as sklm.nn, with s = symmetry op, klm = translation, nn = residue #.
RESIDUE = collection of ARU's constituting an isolated unit (= molecule, ion).
FLAGS : d = determined, c = calculated, R = riding G = group

Atom Types :	C	H	N	O	S
Cov.Rad(Ang):	0.68	0.35	0.68	0.68	1.04
Atom Volume :	13.87	5.08	11.80	11.39	25.20
Atom Number :	6	1	7	8	16
Atom Weight :	12.010	1.008	14.01	16.00	32.06
Scat.Fact.f0:	5.999	1.000	6.995	7.999	16.000
Scat.Fact.f':	0.003	0.000	0.006	0.011	0.125
Scat.Fact.f":	0.002	0.000	0.003	0.006	0.123
Mu/Rho(MoKa):	0.58	0.37	0.84	1.22	9.99
Elem. Type :	--	--	--	--	--

Sources - Cov. Radii : Manual Cambridge Crystallographic Data Base
- Atom Volume: D.W.M. Hofmann (2002). Acta Cryst. B58, 489-493
- Atomic Wt. : SHELXL NOMOVE
- Scat. Fact.: SHELXL (International Tables)
- mu/rho : International Tables C, Table 4.2.4.2, 193-199 - [Multiply by Atom Weight and 1.66054 for Barns/atom values]

Flags	Label	Fractional Coordinates (x,y,z)			Orthogonal Coordinates (XO,YO,ZO)			Site	SSN*SSOF =	S.O.F	Move	Type
d	S(1)	0.72615(12)	0.45508(3)	0.74252(2)	3.6414(6)	8.5042(6)	18.8871(5)	1	1	1	-	D/A
d	S(2)	0.11009(11)	0.76088(3)	0.61998(2)	0.5521(6)	14.2188(6)	15.7701(5)	1	1	1	-	D/A
d	O(1)	0.5950(4)	0.41462(8)	0.34165(5)	2.984(2)	7.7481(15)	8.6904(13)	1	1	1	-	D/A
d	O(2)	0.8653(3)	0.51705(6)	0.52992(5)	4.3392(15)	9.6623(11)	13.4793(13)	1	1	1	-	D/A
d	O(3)	1.3628(3)	0.40269(10)	0.57261(6)	6.8340(15)	7.5252(19)	14.5652(15)	1	1	1	-	D/A
d	O(4)	0.8638(4)	0.28464(7)	0.67573(6)	4.332(2)	5.3192(13)	17.1882(15)	1	1	1	-	D/A
d	O(5)	1.2260(3)	0.33814(8)	0.70915(6)	6.1480(15)	6.3189(15)	18.0383(15)	1	1	1	-	D/A
d	O(6)	0.7103(3)	0.66903(7)	0.52069(5)	3.5619(15)	12.5024(13)	13.2445(13)	1	1	1	-	D/A
d	O(7)	0.1614(3)	0.56930(7)	0.65940(5)	0.8094(15)	10.6387(13)	16.7728(13)	1	1	1	-	D/A
d	O(8)	0.3970(3)	0.64610(7)	0.70896(5)	1.9908(15)	12.0739(13)	18.0335(13)	1	1	1	-	D/A
d	N(1)	0.9272(3)	0.39175(8)	0.59414(6)	4.6496(15)	7.3207(15)	15.1128(15)	1	1	1	-	D/A
d	N(2)	0.3927(3)	0.61475(8)	0.57017(6)	1.9693(15)	11.4880(15)	14.5031(15)	1	1	1	-	D/A
d	C(1)	0.6300(4)	0.43285(10)	0.38704(7)	3.159(2)	8.0888(19)	9.8449(18)	1	1	1	-	-
d	C(2)	0.8268(4)	0.39436(9)	0.42089(7)	4.146(2)	7.3695(17)	10.7060(18)	1	1	1	-	-
d	C(3)	0.9540(4)	0.33419(10)	0.40047(8)	4.784(2)	6.2451(19)	10.187(2)	1	1	1	-	-
d	C(4)	1.1399(5)	0.29758(10)	0.43053(8)	5.716(3)	5.5610(19)	10.951(2)	1	1	1	-	-
d	C(5)	1.1909(4)	0.31988(10)	0.48145(8)	5.972(2)	5.9777(19)	12.246(2)	1	1	1	-	-
d	C(6)	1.0606(4)	0.37888(9)	0.50325(7)	5.319(2)	7.0802(17)	12.8009(18)	1	1	1	-	-
d	C(7)	0.8819(4)	0.41809(9)	0.47201(7)	4.422(2)	7.8130(17)	12.0063(18)	1	1	1	-	-

d	C(8)	0.7736(4)	0.48843(9)	0.49073(6)	3.879(2)	9.1274(17)	12.4825(15)	1	1	1	-	-
d	C(9)	0.5579(4)	0.52272(9)	0.45912(6)	2.798(2)	9.7682(17)	11.6784(15)	1	1	1	-	-
d	C(10)	0.4304(4)	0.58437(9)	0.47781(7)	2.158(2)	10.9203(17)	12.1538(18)	1	1	1	-	-
d	C(11)	0.2157(4)	0.61272(10)	0.44981(7)	1.082(2)	11.4501(19)	11.4416(18)	1	1	1	-	-
d	C(12)	0.1394(4)	0.58322(10)	0.40194(8)	0.699(2)	10.8988(19)	10.224(2)	1	1	1	-	-
d	C(13)	0.2758(4)	0.52540(10)	0.38155(7)	1.383(2)	9.8183(19)	9.7053(18)	1	1	1	-	-
d	C(14)	0.4829(4)	0.49434(10)	0.41026(7)	2.422(2)	9.2379(19)	10.4356(18)	1	1	1	-	-
d	C(15)	1.1287(4)	0.39458(9)	0.55965(7)	5.660(2)	7.3736(17)	14.2355(18)	1	1	1	-	-
d	C(16)	0.9681(4)	0.40581(9)	0.65013(7)	4.855(2)	7.5835(17)	16.5370(18)	1	1	1	-	-
d	C(17)	0.7230(4)	0.44389(10)	0.67185(7)	3.626(2)	8.2951(19)	17.0895(18)	1	1	1	-	-
d	C(18)	1.0102(4)	0.33530(10)	0.67921(7)	5.066(2)	6.2659(19)	17.2767(18)	1	1	1	-	-
d	C(19)	1.2760(5)	0.27564(15)	0.74116(10)	6.399(3)	5.151(3)	18.853(3)	1	1	1	-	-
d	C(20)	0.5274(4)	0.62575(9)	0.52513(7)	2.645(2)	11.6936(17)	13.3575(18)	1	1	1	-	-
d	C(21)	0.4806(4)	0.64832(9)	0.61868(7)	2.410(2)	12.1154(17)	15.7371(18)	1	1	1	-	-
d	C(22)	0.4516(4)	0.72966(9)	0.61812(8)	2.265(2)	13.6354(17)	15.723(2)	1	1	1	-	-
d	C(23)	0.3275(4)	0.61577(9)	0.66404(7)	1.642(2)	11.5071(17)	16.8909(18)	1	1	1	-	-
d	C(24)	0.2502(6)	0.62259(11)	0.75493(8)	1.255(3)	11.635(2)	19.203(2)	1	1	1	-	-
cR	H(1)	0.88193	0.49110	0.74706	4.4226	9.1773	19.0026	1	1	1	-	D-H
cR	H(2)	0.03550	0.74079	0.58269	0.1780	13.8434	14.8216	1	1	1	-	D-H
cR	H(3)	0.76613	0.38116	0.58283	3.8419	7.1229	14.8252	1	1	1	-	D-H
cR	H(4)	0.25072	0.58708	0.57024	1.2573	10.9709	14.5049	1	1	1	-	D-H
cR	H(5)	0.91339	0.31825	0.36592	4.5804	5.9472	9.3077	1	1	1	-	-
cR	H(6)	1.23158	0.25752	0.41630	6.1760	4.8124	10.5892	1	1	1	-	-
cR	H(7)	1.31750	0.29446	0.50203	6.6069	5.5027	12.7699	1	1	1	-	-
cR	H(8)	0.12084	0.65253	0.46358	0.6060	12.1940	11.7919	1	1	1	-	-
cR	H(9)	-0.00704	0.60294	0.38320	-0.0353	11.2673	9.7473	1	1	1	-	-
cR	H(10)	0.22886	0.50678	0.34805	1.1477	9.4704	8.8532	1	1	1	-	-
cR	H(11)	1.12845	0.43696	0.65482	5.6588	8.1656	16.6563	1	1	1	-	-
cR	H(12)	0.56210	0.41633	0.66189	2.8188	7.7801	16.8362	1	1	1	-	-
cR	H(13)	0.70933	0.49163	0.65520	3.5571	9.1872	16.6660	1	1	1	-	-
cR	H(14)	1.14569	0.27370	0.76983	5.7453	5.1147	19.5818	1	1	1	-	-
cR	H(15)	1.45638	0.27836	0.75587	7.3033	5.2018	19.2267	1	1	1	-	-
cR	H(16)	1.25995	0.23250	0.71948	6.3183	4.3448	18.3011	1	1	1	-	-
cR	H(17)	0.67370	0.63678	0.62365	3.3784	11.8997	15.8635	1	1	1	-	-
cR	H(18)	0.53787	0.74852	0.58598	2.6973	13.9878	14.9053	1	1	1	-	-
cR	H(19)	0.54800	0.74958	0.64876	2.7481	14.0076	16.5022	1	1	1	-	-
cR	H(20)	0.31375	0.57521	0.76568	1.5734	10.7491	19.4762	1	1	1	-	-
cR	H(21)	0.05972	0.61999	0.74650	0.2995	11.5859	18.9883	1	1	1	-	-
cR	H(22)	0.27776	0.65668	0.78370	1.3929	12.2716	19.9346	1	1	1	-	-

```
======================================================================================================================================
  Ordered Structure                            Unit Cell Contents (Based on Contents of Atom List, that may be Incomplete)
======================================================================================================================================
Resd Site  X(cen) Y(cen) Z(cen)  Mol.Wt S.O.F   Z      C     H    N    O    S
======================================================================================================================================
  1     1  0.677  0.497  0.567   530.56     1   4     24    22    2    8    2
                                  ----------------------------------------------------------------------------------------------------
       Unit Cell Weight =      2122.22              96    88    8   32    8

 Calculated Analysis (%) =                        54.3   4.2  5.3 24.1 12.1

         Moiety_Formula = C24 H22 N2 O8 S2

            Sum_Formula = C24 H22 N2 O8 S2

         Formula_Weight =      530.56 [Note: Based on SHELXL2014 Atomic Weights]

              Formula_Z =           4

           SpaceGroup_Z =           4  ===>  Z' =  4 /  4 =   1.000

     Calculated Density =   1.4784(1) g cm-3 [= Mg m-3]                          ** WARNING **
                                                                       Please Check the Derived Crystal Data.
                 F(000) =      1104.0 [    1105.56]                    They may be Incorrect for Disordered,
                                                                       Incomplete or Polymeric Structures.
              mu(MoKa) =        2.77 cm-1 =   0.277 mm-1

    Resonant Scattering =          55 * 0.0001 - (E. Girard et al. (2003). Acta Cryst. D59, 1914-1922)

    Friedif            =          90         - (H. Flack & U. Shmueli (2007). Acta Cryst. A63, 257-265)

       Predicted Volume =      2439.0[    2398.5] Ang**3, 298[123]K - (D.W.M. Hofmann (2002). Acta Cryst. B58, 489-493)

Note on F000: The first number is a pure electron count, the second number between [] is calculated from f,f' & f"
```

==

MOLSYM: Search for (additional) Molecular (Point Group) Symmetry - Hydrogen Atoms Excluded ! - Ordered Residues Only !

==

For Details on the Molecular Symmetry Determination see: T. Pilati & A. Forni, J.Appl.Cryst. (1998), 31, 503-504 & (2000), 33, 417.

For CSM (i.e. Continuous Symmetry Measure), see: H. Zabrodsky et al. (1993) JACS, 115, 8278-8289

***** Weighting Mode = ATOMIC *****

:: Resd # 1, No Molecular Symmetry Within Tolerance = 0.80 Ang.

:: Resd # 1, No Molecular Symmetry Within Tolerance = 0.80 Ang.

==

MOLSYM: Search for (additional) Molecular (Point Group) Symmetry - Hydrogen Atoms Excluded ! - Ordered Residues Only !

==

For Details on the Molecular Symmetry Determination see: T. Pilati & A. Forni, J.Appl.Cryst. (1998), 31, 503-504 & (2000), 33, 417.

For CSM (i.e. Continuous Symmetry Measure), see: H. Zabrodsky et al. (1993) JACS, 115, 8278-8289

***** Weighting Mode = UNIT   *****

:: Resd # 1, No Molecular Symmetry Within Tolerance = 0.80 Ang.

:: Resd # 1, No Molecular Symmetry Within Tolerance = 0.80 Ang.

===

NONSYM Search for Additional (Non)Crystallographic Symmetry between Residues (Experimental)

===

- Residue numbers with opposite signs indicate potential enantiomeric pairs
- Hydrogen atoms omitted from the analysis
- Residues with more than 6 atoms are analysed only

RES#	Coords Center of Gravity			Main axes (hor)			EigenV	Asym	Angle a,b,c		
1	0.6783	0.4966	0.5653	0.835	0.546	-0.063	9276	65.02	33	57	94
	3.4013	9.2807	14.3802	-0.474	0.659	-0.584	5175	85.75	118	49	126
	Res.Mol.Wt. =		508.38	-0.278	0.518	0.809	4799	120.92	106	59	36

(An)isotropic, Equivalent and Main Axes Displacement Parameters - Unusual Values Marked with a # - [Optional Coordinate Split-up]

Atom	Label	U11 or Uiso	U22	U33	U23	U13	U12	Ueq(sUeq)	U1	U2	U3	U3/U1
1	S1	0.0331(3)	0.0502(3)	0.0290(3)	-0.0137(2)	0.0015(2)	0.0013(3)	0.03743(17)	0.0219	0.0334	0.0569	2.59
		[0.7276	0.4596	0.7413]	[0.7247	0.4505	0.7437]					
2	S2	0.0266(2)	0.0316(2)	0.0355(3)	-0.0019(2)	0.0002(2)	0.0061(2)	0.03123(14)	0.0224	0.0342	0.0371	1.66
		[0.1174	0.7618	0.6194]	[0.1028	0.7599	0.6205]					
3	O1	0.0499(10)	0.0462(9)	0.0194(7)	-0.0087(6)	-0.0103(7)	0.0066(8)	0.0385(5)	0.0149	0.0412	0.0594	4.00
		[0.6187	0.4160	0.3413]	[0.5713	0.4133	0.3420]					
4	O2	0.0254(7)	0.0223(6)	0.0176(6)	0.0001(5)	-0.0039(5)	-0.0003(6)	0.0218(4)	0.0160	0.0223	0.0270	1.69
		[0.8729	0.5170	0.5298]	[0.8577	0.5171	0.5300]					
5	O3	0.0113(6)	0.0662(10)	0.0313(8)	-0.0057(7)	-0.0021(6)	-0.0009(7)	0.0363(5)	0.0110	0.0306	0.0671	6.08
		[1.3616	0.4109	0.5719]	[1.3640	0.3945	0.5733]					
6	O4	0.0528(9)	0.0235(7)	0.0335(8)	0.0068(5)	-0.0081(7)	-0.0100(7)	0.0366(5)	0.0190	0.0312	0.0596	3.14
		[0.8848	0.2841	0.6754]	[0.8428	0.2852	0.6761]					
7	O5	0.0225(7)	0.0441(8)	0.0326(7)	0.0197(6)	-0.0057(6)	0.0027(7)	0.0331(4)	0.0135	0.0267	0.0589	4.35
		[1.2224	0.3440	0.7115]	[1.2296	0.3323	0.7068]					
8	O6	0.0255(7)	0.0238(6)	0.0274(7)	0.0002(5)	0.0061(6)	-0.0042(6)	0.0256(4)	0.0182	0.0252	0.0334	1.84
		[0.7196	0.6687	0.5211]	[0.7010	0.6693	0.5203]					
9	O7	0.0371(8)	0.0268(7)	0.0256(7)	-0.0015(5)	0.0068(6)	-0.0127(6)	0.0298(4)	0.0173	0.0246	0.0476	2.75
		[0.1784	0.5685	0.6596]	[0.1444	0.5701	0.6592]					
10	O8	0.0427(9)	0.0326(7)	0.0199(6)	-0.0021(5)	0.0032(6)	-0.0140(7)	0.0317(4)	0.0195	0.0228	0.0530	2.72
		[0.4148	0.6452	0.7091]	[0.3792	0.6470	0.7089]					
11	N1	0.0122(7)	0.0232(7)	0.0187(7)	0.0038(6)	-0.0020(6)	-0.0024(6)	0.0180(4)	0.0115	0.0166	0.0261	2.27
		[0.9200	0.3938	0.5948]	[0.9344	0.3897	0.5935]					
12	N2	0.0198(7)	0.0219(7)	0.0225(7)	-0.0016(6)	0.0035(6)	-0.0041(7)	0.0214(4)	0.0160	0.0207	0.0275	1.72
		[0.3853	0.6153	0.5699]	[0.4001	0.6142	0.5705]					
13	C1	0.0264(9)	0.0288(9)	0.0186(8)	-0.0006(7)	-0.0010(8)	-0.0040(8)	0.0246(5)	0.0183	0.0237	0.0318	1.73
		[0.6220	0.4336	0.3870]	[0.6380	0.4321	0.3870]					
14	C2	0.0207(9)	0.0221(8)	0.0198(8)	-0.0003(7)	0.0022(7)	-0.0034(7)	0.0209(5)	0.0170	0.0202	0.0254	1.49
		[0.8213	0.3948	0.4208]	[0.8323	0.3939	0.4210]					
15	C3	0.0343(11)	0.0288(10)	0.0197(9)	-0.0057(7)	0.0056(8)	-0.0035(9)	0.0276(6)	0.0162	0.0276	0.0390	2.41
		[0.9678	0.3336	0.4007]	[0.9402	0.3348	0.4002]					
16	C4	0.0333(11)	0.0239(9)	0.0323(10)	-0.0047(7)	0.0083(9)	0.0041(9)	0.0298(6)	0.0180	0.0304	0.0411	2.29
		[1.1534	0.2976	0.4310]	[1.1264	0.2976	0.4300]					
17	C5	0.0226(10)	0.0242(9)	0.0301(10)	0.0034(7)	0.0029(8)	0.0037(7)	0.0256(6)	0.0196	0.0239	0.0333	1.70
		[1.1984	0.3205	0.4821]	[1.1834	0.3192	0.4808]					
18	C6	0.0134(8)	0.0216(8)	0.0233(9)	0.0015(7)	0.0028(6)	-0.0019(7)	0.0194(5)	0.0121	0.0217	0.0244	2.01
		[1.0664	0.3798	0.5045]	[1.0548	0.3780	0.5020]					
19	C7	0.0144(8)	0.0213(8)	0.0172(8)	0.0010(6)	0.0016(7)	-0.0027(7)	0.0176(5)	0.0127	0.0179	0.0223	1.75
		[0.8763	0.4193	0.4721]	[0.8875	0.4168	0.4719]					
20	C8	0.0156(8)	0.0196(8)	0.0153(7)	0.0025(6)	-0.0006(6)	-0.0020(7)	0.0168(4)	0.0141	0.0150	0.0215	1.53
		[0.7691	0.4893	0.4909]	[0.7781	0.4876	0.4905]					
21	C9	0.0149(8)	0.0204(8)	0.0168(8)	0.0043(6)	0.0012(6)	-0.0016(7)	0.0174(5)	0.0125	0.0163	0.0233	1.87

		[0.5553	0.5246	0.4598]	[0.5605	0.5208	0.4585]					
22	C10	0.0170(8)	0.0225(8)	0.0194(8)	0.0049(7)	0.0036(7)	-0.0020(7)	0.0196(5)	0.0123	0.0205	0.0261	2.13
		[0.4331	0.5866	0.4788]	[0.4277	0.5821	0.4769]					
23	C11	0.0187(9)	0.0255(9)	0.0266(9)	0.0078(7)	0.0038(7)	0.0021(8)	0.0236(5)	0.0169	0.0190	0.0350	2.07
		[0.2242	0.6143	0.4508]	[0.2072	0.6111	0.4489]					
24	C12	0.0195(9)	0.0313(10)	0.0295(10)	0.0129(8)	-0.0032(8)	-0.0024(8)	0.0268(6)	0.0172	0.0192	0.0440	2.56
		[0.1306	0.5861	0.4034]	[0.1482	0.5804	0.4005]					
25	C13	0.0231(9)	0.0307(9)	0.0207(8)	0.0052(7)	-0.0055(8)	-0.0057(8)	0.0248(5)	0.0162	0.0213	0.0370	2.28
		[0.2641	0.5268	0.3819]	[0.2875	0.5240	0.3812]					
26	C14	0.0200(9)	0.0246(9)	0.0179(8)	0.0030(7)	-0.0013(7)	-0.0052(7)	0.0208(5)	0.0162	0.0173	0.0289	1.78
		[0.4751	0.4952	0.4104]	[0.4907	0.4935	0.4101]					
27	C15	0.0144(8)	0.0209(8)	0.0244(9)	0.0028(6)	-0.0020(7)	0.0006(7)	0.0199(5)	0.0138	0.0197	0.0261	1.89
		[1.1242	0.3957	0.5609]	[1.1332	0.3934	0.5584]					
28	C16	0.0154(8)	0.0200(8)	0.0208(8)	0.0024(6)	-0.0017(7)	-0.0004(7)	0.0187(5)	0.0149	0.0182	0.0232	1.55
		[0.9641	0.4067	0.6507]	[0.9721	0.4049	0.6495]					
29	C17	0.0193(9)	0.0289(9)	0.0248(9)	-0.0021(7)	-0.0017(8)	0.0024(8)	0.0243(5)	0.0185	0.0240	0.0305	1.65
		[0.7285	0.4453	0.6715]	[0.7175	0.4425	0.6722]					
30	C18	0.0240(9)	0.0265(9)	0.0179(8)	0.0013(7)	0.0008(7)	0.0039(8)	0.0228(5)	0.0177	0.0212	0.0295	1.67
		[1.0175	0.3360	0.6793]	[1.0029	0.3346	0.6791]					
31	C19	0.0372(12)	0.0612(15)	0.0399(12)	0.0319(11)	-0.0009(11)	0.0121(12)	0.0461(8)	0.0141	0.0382	0.0860	6.08
		[1.2974	0.2822	0.7436]	[1.2546	0.2691	0.7387]					
32	C20	0.0196(8)	0.0188(8)	0.0219(9)	0.0036(7)	0.0029(7)	0.0035(7)	0.0201(5)	0.0155	0.0179	0.0269	1.73
		[0.5348	0.6263	0.5255]	[0.5200	0.6252	0.5248]					
33	C21	0.0210(9)	0.0220(8)	0.0215(8)	-0.0001(7)	0.0037(7)	-0.0021(7)	0.0215(5)	0.0171	0.0218	0.0256	1.50
		[0.4749	0.6486	0.6185]	[0.4863	0.6481	0.6189]					
34	C22	0.0234(9)	0.0205(9)	0.0278(9)	-0.0001(7)	0.0033(8)	-0.0019(7)	0.0239(5)	0.0193	0.0227	0.0297	1.54
		[0.4579	0.7296	0.6186]	[0.4453	0.7298	0.6177]					
35	C23	0.0251(10)	0.0192(8)	0.0219(9)	0.0003(7)	0.0031(7)	-0.0015(7)	0.0221(5)	0.0184	0.0207	0.0272	1.48
		[0.3333	0.6157	0.6642]	[0.3217	0.6158	0.6639]					
36	C24	0.0578(14)	0.0354(11)	0.0206(9)	-0.0014(8)	0.0083(10)	-0.0092(11)	0.0379(7)	0.0188	0.0323	0.0627	3.33
		[0.2727	0.6220	0.7551]	[0.2277	0.6232	0.7547]					
37	H1	0.05780						0.05780				
38	H2	0.04700						0.04700				
39	H3	0.02160						0.02160				
40	H4	0.02570						0.02570				
41	H5	0.03310						0.03310				
42	H6	0.03580						0.03580				
43	H7	0.03070						0.03070				
44	H8	0.02830						0.02830				
45	H9	0.03210						0.03210				
46	H10	0.02980						0.02980				
47	H11	0.02250						0.02250				
48	H12	0.02920						0.02920				
49	H13	0.02920						0.02920				
50	H14	0.05540						0.05540				
51	H15	0.05540						0.05540				
52	H16	0.05540						0.05540				
53	H17	0.02580						0.02580				

54	H18	0.02870						0.02870					
55	H19	0.02870						0.02870					
56	H20	0.04550						0.04550					
57	H21	0.04550						0.04550					
58	H22	0.04550						0.04550					
U(i,j)-Average		0.0253	0.0286	0.0239	0.0019	0.0007	-0.0014	0.0260	0.0227	0.0255	0.0296	1.30	

The Displacement Factor has the Form of Exp(-T)

where

T = 8*(pi**2)*Uiso*sin(theta/lambda)**2, for Isotropic Atoms,

T = 2*(pi**2)*(U11*(h*as)**2+U22*(k*bs)**2+U33*(l*cs)**2+2*U23*k*l*bs*cs+2*U13*h*l*as*cs+2*U12*h*k*as*bs),for Anisotr. Atoms

Ueq = 1/3 Sum(i,j) (Uij*as(i)*as(j)*a(i).a(j))

U1, U2, U3 are the three Main Axes Components of Uij

Reference U(eq): R.X. Fischer & E. Tillmanns, Acta Cryst. (1988). C44, 775-776

Ueq [or U(iso)] Averages per Element

	Non-H	C	H	N	O	S
Average	0.0259	0.0239	0.0368	0.0197	0.0317	0.0343
Minimum	0.0168	0.0168	0.0216	0.0180	0.0218	0.0312
Maximum	0.0461	0.0461	0.0578	0.0214	0.0385	0.0374
Ratio	2.7440	2.7440	2.6759	1.1889	1.7661	1.1985
Number	36	24	22	2	8	2

V.Schomaker and K.N.Trueblood Rigid Body Motion Analysis, TLS - Model (Acta Cryst. (1968), B24, 63-76) - see also Dunitz, p244

Observed Vibration Tensor in Inertial System I(1) = L, I(2) = M, I(3) = N (Difference U(calc) - U(obs) in Parentheses)

Label	U(L,L)<br>U11	U(L,M)<br>U12	U(L,N)<br>U13	U(M,M)<br>U22	U(M,N)<br>U23	U(N,N)<br>U33	Ueq(obs)	Ueq(cal)
S(1)	0.02247[0.00748]	-0.00373[-.00854]	0.00084[0.00059]	0.04968[-.02044]	-0.01063[0.01089]	0.04015[-.00629]	0.0374	0.0310
	0.03310[0.00220]	0.00130[-.00423]	0.00150[-.00351]	0.05020[-.01040]	-0.01370[0.01735]	0.02900[-.01104]		
S(2)	0.03033[-.00271]	0.00560[0.00409]	0.00204[0.00197]	0.02957[0.00947]	-0.00449[0.01309]	0.03380[-.00124]	0.0312	0.0331
	0.02660[0.00944]	0.00610[-.00464]	0.00020[0.01157]	0.03160[-.00808]	-0.00190[-.00016]	0.03550[0.00416]		
O(1)	0.02438[-.00052]	-0.01061[0.00104]	-0.00834[0.00524]	0.03475[0.00054]	-0.00733[0.00630]	0.05638[-.01935]	0.0385	0.0321
	0.04990[-.01113]	0.00660[-.00883]	-0.01030[0.00803]	0.04620[-.00795]	-0.00870[0.00441]	0.01940[-.00024]		
O(2)	0.02138[0.00011]	-0.00365[0.00126]	-0.00323[0.00431]	0.01934[0.00090]	-0.00026[-.00102]	0.02457[-.01308]	0.0218	0.0177
	0.02540[-.01206]	-0.00030[-.00421]	-0.00390[0.00371]	0.02230[-.00116]	0.00010[0.00125]	0.01760[0.00116]		
O(3)	0.03554[-.00120]	-0.00881[0.00816]	0.01047[-.00556]	0.04572[-.02327]	-0.02323[0.01748]	0.02754[-.00656]	0.0363	0.0259
	0.01130[0.00438]	-0.00090[0.00129]	-0.00210[0.00013]	0.06620[-.03365]	-0.00570[0.01082]	0.03130[-.00176]		
O(4)	0.04452[-.00595]	-0.00678[0.00387]	-0.01295[0.01844]	0.03003[-.01001]	0.01289[-.00956]	0.03525[-.00346]	0.0366	0.0301
	0.05280[-.02228]	-0.01000[0.01203]	-0.00810[0.00800]	0.02350[0.00770]	0.00680[0.00469]	0.03350[-.00484]		
O(5)	0.05320[-.00658]	-0.00723[-.00171]	0.01046[-.00610]	0.01531[0.00567]	-0.00444[-.00087]	0.03069[0.00826]	0.0331	0.0355
	0.02250[0.00913]	0.00270[0.00075]	-0.00570[-.00239]	0.04410[0.00150]	0.01970[-.00764]	0.03260[-.00327]		
O(6)	0.02492[0.00089]	0.00098[-.00063]	0.00255[-.00163]	0.03126[-.00609]	0.00463[-.00133]	0.02052[-.00611]	0.0256	0.0218
	0.02550[-.00569]	-0.00420[-.00135]	0.00610[-.00345]	0.02380[-.00377]	0.00020[0.00192]	0.02740[-.00185]		
O(7)	0.02615[-.00427]	-0.00551[0.00516]	-0.00180[0.00640]	0.04156[-.01177]	0.01040[-.00726]	0.02179[-.00069]	0.0298	0.0243
	0.03710[-.01355]	-0.01270[0.01010]	0.00680[-.00002]	0.02680[-.00114]	-0.00150[0.00158]	0.02560[-.00205]		
O(8)	0.02589[-.00563]	-0.01214[0.00898]	-0.00427[0.00341]	0.04267[-.00143]	0.00925[-.00449]	0.02664[0.00471]	0.0317	0.0310
	0.04270[-.00497]	-0.01400[0.00952]	0.00320[0.00291]	0.03260[-.00170]	-0.00210[-.00426]	0.01990[0.00432]		
N(1)	0.02418[0.00176]	-0.00302[0.00077]	0.00227[-.00040]	0.01666[0.00232]	-0.00304[0.00150]	0.01326[0.00410]	0.0180	0.0208
	0.01220[0.00469]	-0.00240[0.00067]	-0.00200[0.00061]	0.02320[0.00087]	0.00380[-.00009]	0.01870[0.00262]		
N(2)	0.02040[0.00145]	-0.00005[0.00028]	0.00108[0.00026]	0.02736[-.00430]	0.00137[-.00009]	0.01644[-.00180]	0.0214	0.0198
	0.01980[-.00238]	-0.00410[0.00053]	0.00350[-.00122]	0.02190[-.00199]	-0.00160[0.00234]	0.02250[-.00028]		
C(1)	0.02303[-.00098]	-0.00630[-.00004]	-0.00099[-.00030]	0.02718[0.00120]	-0.00009[-.00086]	0.02358[0.00341]	0.0246	0.0258
	0.02640[0.00205]	-0.00400[0.00147]	-0.00100[-.00024]	0.02880[0.00175]	-0.00060[-.00141]	0.01860[-.00016]		
C(2)	0.02022[-.00006]	-0.00180[-.00063]	0.00089[-.00045]	0.02457[0.00305]	0.00142[-.00111]	0.01780[0.00636]	0.0209	0.0240
	0.02070[0.00462]	-0.00340[0.00188]	0.00220[-.00043]	0.02210[0.00418]	-0.00030[-.00185]	0.01980[0.00056]		
C(3)	0.01698[0.00353]	-0.00397[0.00395]	-0.00025[0.00113]	0.03661[-.00203]	0.00404[-.00178]	0.02921[0.00347]	0.0276	0.0293
	0.03430[-.00074]	-0.00350[0.00424]	0.00560[-.00069]	0.02880[0.00033]	-0.00570[0.00105]	0.01970[0.00538]		
C(4)	0.02128[0.00217]	0.00741[-.00285]	0.00238[0.00162]	0.03454[-.00046]	0.00433[-.00140]	0.03369[0.00043]	0.0298	0.0305
	0.03330[-.00075]	0.00410[-.00033]	0.00830[-.00079]	0.02390[0.00438]	-0.00470[0.00189]	0.03230[-.00150]		
C(5)	0.02842[-.00228]	0.00445[-.00021]	0.00451[0.00067]	0.02253[0.00461]	0.00063[0.00002]	0.02594[0.00147]	0.0256	0.0269
	0.02260[0.00165]	0.00370[-.00035]	0.00290[0.00214]	0.02420[0.00233]	0.00340[-.00245]	0.03010[-.00018]		
C(6)	0.02263[0.00105]	0.00134[-.00059]	0.00384[-.00059]	0.02192[0.00059]	-0.00093[0.00008]	0.01375[0.00565]	0.0194	0.0219
	0.01340[0.00465]	-0.00190[0.00179]	0.00280[-.00087]	0.02160[0.00223]	0.00150[-.00006]	0.02330[0.00041]		
C(7)	0.01898[0.00161]	-0.00206[0.00070]	0.00257[-.00098]	0.02015[0.00236]	-0.00070[0.00018]	0.01377[0.00374]	0.0176	0.0202
	0.01440[0.00368]	-0.00270[0.00071]	0.00160[-.00034]	0.02130[0.00141]	0.00100[-.00086]	0.01720[0.00262]		
C(8)	0.01942[0.00106]	-0.00307[0.00060]	0.00119[0.00007]	0.01623[0.00368]	-0.00022[-.00072]	0.01486[-.00160]	0.0168	0.0179
	0.01560[-.00097]	-0.00200[-.00155]	-0.00060[0.00072]	0.01960[0.00155]	0.00250[-.00128]	0.01530[0.00256]		

C(9)	0.02114[0.00101]	-0.00239[-.00122]	0.00360[-.00269]	0.01629[0.00347]	0.00052[-.00160]	0.01467[0.00080]	0.0174	0.0191
	0.01490[0.00172]	-0.00160[-.00168]	0.00120[-.00218]	0.02040[0.00247]	0.00430[-.00234]	0.01680[0.00109]		
C(10)	0.02311[0.00131]	-0.00193[-.00021]	0.00537[-.00357]	0.01968[-.00066]	0.00193[-.00238]	0.01611[-.00072]	0.0196	0.0196
	0.01700[-.00072]	-0.00200[-.00071]	0.00360[-.00406]	0.02250[-.00031]	0.00490[-.00144]	0.01940[0.00097]		
C(11)	0.02992[0.00093]	0.00136[-.00366]	0.00794[-.00505]	0.01914[-.00017]	0.00121[-.00284]	0.02174[-.00079]	0.0236	0.0236
	0.01870[0.00054]	0.00210[-.00260]	0.00380[-.00583]	0.02550[0.00162]	0.00780[-.00177]	0.02660[-.00219]		
C(12)	0.04215[-.00681]	-0.00400[-.00231]	0.00521[-.00372]	0.01783[0.00276]	-0.00073[-.00367]	0.02032[0.00661]	0.0268	0.0276
	0.01950[0.00414]	-0.00240[0.00217]	-0.00320[-.00283]	0.03130[0.00344]	0.01290[-.00646]	0.02950[-.00503]		
C(13)	0.03204[-.00108]	-0.00858[-.00018]	-0.00102[0.00011]	0.02209[0.00166]	-0.00163[-.00259]	0.02038[0.00736]	0.0248	0.0275
	0.02310[0.00338]	-0.00570[0.00370]	-0.00550[-.00083]	0.03070[0.00467]	0.00520[-.00219]	0.02070[-.00010]		
C(14)	0.02445[-.00023]	-0.00577[-.00043]	0.00027[-.00069]	0.02150[0.00145]	0.00042[-.00248]	0.01655[0.00528]	0.0208	0.0230
	0.02000[0.00247]	-0.00520[0.00233]	-0.00130[-.00154]	0.02460[0.00382]	0.00300[-.00191]	0.01790[0.00021]		
C(15)	0.02576[0.00164]	0.00168[-.00236]	0.00126[0.00200]	0.01699[0.00332]	-0.00279[-.00003]	0.01694[0.00128]	0.0199	0.0220
	0.01440[0.00143]	0.00060[-.00113]	-0.00200[0.00133]	0.02090[0.00502]	0.00280[0.00072]	0.02440[-.00022]		
C(16)	0.02306[0.00672]	-0.00012[-.00550]	0.00072[0.00094]	0.01670[0.00349]	-0.00165[-.00097]	0.01644[0.00541]	0.0187	0.0239
	0.01540[0.00532]	-0.00040[-.00140]	-0.00170[-.00184]	0.02000[0.00991]	0.00240[0.00259]	0.02080[0.00039]		
C(17)	0.02379[0.00191]	0.00046[-.00713]	0.00107[0.00024]	0.02452[-.00201]	-0.00589[0.00551]	0.02470[-.00348]	0.0243	0.0231
	0.01930[0.00340]	0.00240[-.00583]	-0.00170[0.00031]	0.02890[-.00035]	-0.00210[0.00542]	0.02480[-.00662]		
C(18)	0.02029[0.01718]	-0.00219[-.00344]	0.00320[0.00095]	0.02001[-.00043]	-0.00098[-.00016]	0.02811[0.00255]	0.0228	0.0292
	0.02400[0.00334]	0.00390[-.00233]	0.00080[-.00429]	0.02650[0.00818]	0.00130[0.00840]	0.01790[0.00778]		
C(19)	0.06907[-.01079]	-0.00927[-.00029]	0.02248[-.01462]	0.01592[0.00443]	-0.00117[-.00262]	0.05331[-.00098]	0.0461	0.0437
	0.03720[0.00427]	0.01210[-.00375]	-0.00090[-.00770]	0.06120[-.00745]	0.03190[-.01305]	0.03990[-.00416]		
C(20)	0.02160[0.00164]	0.00267[-.00300]	0.00412[-.00278]	0.01669[0.00477]	0.00181[-.00043]	0.02201[-.00811]	0.0201	0.0195
	0.01960[-.00272]	0.00350[-.00736]	0.00290[-.00141]	0.01880[0.00087]	0.00360[-.00147]	0.02190[0.00015]		
C(21)	0.02045[0.00075]	-0.00016[0.00007]	0.00219[-.00277]	0.02504[0.00453]	0.00189[0.00089]	0.01900[-.00058]	0.0215	0.0231
	0.02100[0.00264]	-0.00210[-.00280]	0.00370[-.00038]	0.02200[-.00026]	-0.00010[-.00224]	0.02150[0.00232]		
C(22)	0.02448[-.00028]	0.00301[0.00072]	0.00067[-.00216]	0.02674[0.01014]	0.00291[0.00351]	0.02048[0.00366]	0.0239	0.0284
	0.02340[0.00841]	-0.00190[-.00278]	0.00330[0.00296]	0.02050[0.00117]	-0.00010[-.00393]	0.02780[0.00394]		
C(23)	0.02071[-.00040]	0.00047[-.00157]	0.00034[0.00111]	0.02387[0.00836]	0.00420[-.00083]	0.02161[0.00128]	0.0221	0.0251
	0.02510[0.00199]	-0.00150[-.00209]	0.00310[0.00238]	0.01920[0.00619]	0.00030[-.00291]	0.02190[0.00106]		
C(24)	0.02518[-.00446]	-0.01173[0.00686]	-0.00185[0.00463]	0.04681[0.00029]	0.01540[-.00838]	0.04181[-.00100]	0.0379	0.0362
	0.05780[-.01157]	-0.00920[0.00678]	0.00830[0.00202]	0.03540[0.00260]	-0.00140[-.00465]	0.02060[0.00381]		

```
R1 = Sum(abs(U(obs)-U(calc)))/Sum(abs(U(obs)))      =   0.23000

R2 = Sqrt(Sum((U(obs)-U(calc))**2)/Sum(U(obs)**2)) =   0.26105

S  = Sqrt(Sum((U(obs)-U(calc))**2)/(6*N-NS*M))      =   0.00552

N  = Number of Atoms in Rigid Group                 =        36

NS = Symmetry Factor                                =         1

M  = Number of Rigid-Body Parameters                =        20

     Largest abs(U(obs)-U(calc))                    =   0.02327

                                                       TLS-Mode

:: No TLS-Analysis for Residue Nr:  1, Because R >  0.25
```

Rigid-Body Model Libration Corrections for Bond Distances and "Hirshfeld Rigid-Bond" Test (Acta Cryst., 1976, A32, 239-244)

Bond		Bond Distance	Components of the Correction				MSDA from U(obs) Vibration Along the Interatomic Bond				Angle with Lib. Axes		
Atom(I)	Atom(J)	Obsd	Calcd	Del(L)	Del(M)	Del(N)	I to J	J to I	Difference	Sqrt(Diff)	L(1)	L(2)	L(3)
S(1)	- C(17)	1.8098(19)	0	0	0	0	0.0262(3)	0.0244(9)	0.0018(9)	0.0424	0.00	0.00	0.00
S(2)	- C(22)	1.810(2)	0	0	0	0	0.0234(2)	0.0241(9)	0.0007(9)	0.0265	0.00	0.00	0.00
O(1)	- C(1)	1.217(2)	0	0	0	0	0.0152(9)	0.0186(9)	0.0034(12)	0.0583	0.00	0.00	0.00
O(2)	- C(8)	1.221(2)	0	0	0	0	0.0172(6)	0.0169(8)	0.0003(10)	0.0173	0.00	0.00	0.00
O(3)	- C(15)	1.229(2)	0	0	0	0	0.0119(8)	0.0145(8)	0.0026(12)	0.0510	0.00	0.00	0.00
O(4)	- C(18)	1.201(3)	0	0	0	0	0.0249(8)	0.0295(9)	0.0046(12)	0.0678	0.00	0.00	0.00
O(5)	- C(18)	1.324(2)	0	0	0	0	0.0216(7)	0.0231(9)	0.0015(11)	0.0387	0.00	0.00	0.00
O(5)	- C(19)	1.446(3)	0	0	0	0	0.0200(7)	0.0211(13)	0.0011(15)	0.0332	0.00	0.00	0.00
O(6)	- C(20)	1.228(2)	0	0	0	0	0.0198(7)	0.0219(8)	0.0021(11)	0.0458	0.00	0.00	0.00
O(7)	- C(23)	1.209(2)	0	0	0	0	0.0198(7)	0.0210(9)	0.0012(12)	0.0346	0.00	0.00	0.00
O(8)	- C(23)	1.322(2)	0	0	0	0	0.0206(7)	0.0229(9)	0.0023(12)	0.0480	0.00	0.00	0.00
O(8)	- C(24)	1.450(3)	0	0	0	0	0.0210(7)	0.0226(11)	0.0016(13)	0.0400	0.00	0.00	0.00
N(1)	- C(15)	1.339(2)	0	0	0	0	0.0167(7)	0.0206(8)	0.0039(11)	0.0624	0.00	0.00	0.00
N(1)	- C(16)	1.463(2)	0	0	0	0	0.0194(7)	0.0210(8)	0.0016(11)	0.0400	0.00	0.00	0.00
N(2)	- C(20)	1.346(2)	0	0	0	0	0.0186(7)	0.0184(8)	0.0002(11)	0.0141	0.00	0.00	0.00
N(2)	- C(21)	1.453(2)	0	0	0	0	0.0217(7)	0.0228(8)	0.0011(11)	0.0332	0.00	0.00	0.00
C(1)	- C(2)	1.494(3)	0	0	0	0	0.0265(9)	0.0247(8)	0.0018(12)	0.0424	0.00	0.00	0.00
C(1)	- C(14)	1.488(3)	0	0	0	0	0.0297(9)	0.0287(9)	0.0010(12)	0.0316	0.00	0.00	0.00
C(2)	- C(3)	1.393(3)	0	0	0	0	0.0231(8)	0.0259(10)	0.0028(13)	0.0529	0.00	0.00	0.00
C(2)	- C(7)	1.401(3)	0	0	0	0	0.0203(8)	0.0184(8)	0.0019(12)	0.0436	0.00	0.00	0.00
C(3)	- C(4)	1.386(3)	0	0	0	0	0.0381(10)	0.0367(10)	0.0014(14)	0.0374	0.00	0.00	0.00
C(4)	- C(5)	1.384(3)	0	0	0	0	0.0323(10)	0.0327(10)	0.0004(14)	0.0200	0.00	0.00	0.00
C(5)	- C(6)	1.396(3)	0	0	0	0	0.0231(10)	0.0214(8)	0.0017(13)	0.0412	0.00	0.00	0.00
C(6)	- C(7)	1.404(3)	0	0	0	0	0.0212(8)	0.0195(8)	0.0017(12)	0.0412	0.00	0.00	0.00
C(6)	- C(15)	1.504(3)	0	0	0	0	0.0243(8)	0.0240(8)	0.0003(12)	0.0173	0.00	0.00	0.00
C(7)	- C(8)	1.500(2)	0	0	0	0	0.0219(8)	0.0214(8)	0.0005(11)	0.0224	0.00	0.00	0.00
C(8)	- C(9)	1.492(3)	0	0	0	0	0.0159(8)	0.0164(8)	0.0005(11)	0.0224	0.00	0.00	0.00
C(9)	- C(10)	1.401(2)	0	0	0	0	0.0221(8)	0.0241(8)	0.0020(11)	0.0447	0.00	0.00	0.00
C(9)	- C(14)	1.403(2)	0	0	0	0	0.0203(8)	0.0193(9)	0.0010(12)	0.0316	0.00	0.00	0.00
C(10)	- C(11)	1.395(3)	0	0	0	0	0.0205(8)	0.0205(9)	0.0000(12)	0	0.00	0.00	0.00
C(10)	- C(20)	1.511(3)	0	0	0	0	0.0252(8)	0.0264(8)	0.0012(12)	0.0346	0.00	0.00	0.00
C(11)	- C(12)	1.390(3)	0	0	0	0	0.0336(9)	0.0359(10)	0.0023(13)	0.0480	0.00	0.00	0.00
C(12)	- C(13)	1.380(3)	0	0	0	0	0.0388(10)	0.0370(9)	0.0018(13)	0.0424	0.00	0.00	0.00
C(13)	- C(14)	1.396(3)	0	0	0	0	0.0207(9)	0.0211(9)	0.0004(12)	0.0200	0.00	0.00	0.00
C(16)	- C(17)	1.524(3)	0	0	0	0	0.0192(8)	0.0206(9)	0.0014(12)	0.0374	0.00	0.00	0.00
C(16)	- C(18)	1.526(3)	0	0	0	0	0.0180(8)	0.0225(9)	0.0045(12)	0.0671	0.00	0.00	0.00
C(21)	- C(22)	1.527(2)	0	0	0	0	0.0224(8)	0.0209(9)	0.0015(12)	0.0387	0.00	0.00	0.00
C(21)	- C(23)	1.514(3)	0	0	0	0	0.0178(8)	0.0191(9)	0.0013(12)	0.0361	0.00	0.00	0.00

Sqrt(Sum(DelIJ**2)/Nrb) =   0.0020

# - Indicates bonds exceeding the 5.0 sigma test level

==

Test Matrix for Rigid-Body Vibrations - /Del(A,B)/ = /Z(A,B)**2 - Z(B,A)**2/ Should be Near Zero (Acta Cryst. A34, 1978, 828)

==

Atom-Atom		1	2	3	4	5	6	7	8	9	10	11	12	13	14	15	16	17	18	19	20	21	22	23	24	25	26	27	28	29	30	31	32	33	34	35	36
1 S(1)	-	0	24	10	16	1	3	4	23	2	6	1	21	10	6	7	2	6	2	9	17	19	22	19	17	16	15	1	1	-2	7	2	28	30	31	27	11
2 S(2)	-	7	0	3	1	18	5	0	3	3	9	2	3	3	5	4	7	2	4	5	4	6	5	0	9	1	3	4	7	1	4	2	7	0	-1	7	3
3 O(1)	-	10	10	0	1	4	5	4	1	2	2	0	1	-3	2	5	3	1	3	1	1	2	0	4	0	1	0	3	3	7	1	4	1	1	2	1	1
4 O(2)	-	6	6	5	0	14	1	3	4	3	5	2	4	2	1	2	4	0	1	0	0	0	0	0	0	1	1	4	0	8	1	4	8	3	1	3	2
5 O(3)	-	5	9	7	3	0	6	14	22	16	7	1	9	2	0	2	0	0	1	3	11	11	12	12	10	7	5	-3	3	1	15	25	24	17	23	9	10
6 O(4)	-	4	10	9	6	4	0	1	4	2	6	5	1	7	8	12	8	11	10	9	8	9	6	8	10	8	8	7	0	2	-5	4	8	4	5	13	0
7 O(5)	-	3	10	10	6	4	2	0	12	24	15	2	10	1	3	9	4	4	2	3	1	1	6	7	4	2	1	0	3	2	-1	-1	1	1	2	2	15
8 O(6)	-	7	4	7	3	6	8	8	0	0	3	6	2	2	3	2	6	4	4	3	4	2	2	0	2	6	4	9	10	4	6	11	-2	3	0	2	3
9 O(7)	-	4	4	9	5	7	6	7	5	0	1	18	1	3	7	10	13	11	14	11	9	6	6	1	2	1	3	18	27	25	24	21	2	0	1	-1	1
10 O(8)	-	4	3	10	6	7	7	7	5	2	0	13	2	1	5	10	12	9	11	8	6	1	2	8	11	3	1	15	24	17	22	11	2	1	1	-2	-2
11 N(1)	-	4	8	7	3	2	3	3	6	5	6	0	5	3	2	3	1	4	1	0	2	0	3	3	2	2	3	-4	-2	1	2	0	4	1	2	2	11
12 N(2)	-	6	3	7	3	6	7	8	2	3	4	5	0	2	1	0	4	1	3	1	0	1	0	4	7	2	2	7	10	1	6	9	0	-1	2	0	2
13 C(1)	-	9	9	-1	4	6	8	9	6	8	9	6	6	0	-2	5	0	4	0	2	2	1	1	4	1	0	-1	1	0	6	2	1	1	1	0	0	1
14 C(2)	-	8	9	2	4	5	7	8	6	8	9	4	6	-1	0	-3	1	2	1	-2	1	1	1	2	1	2	2	0	0	5	3	2	2	0	2	0	4
15 C(3)	-	9	11	3	5	5	7	8	7	9	10	5	7	2	-1	0	-1	0	5	2	1	4	2	7	6	5	5	5	4	8	2	8	3	2	2	2	8
16 C(4)	-	9	11	4	5	4	6	7	8	9	10	5	8	4	2	-1	0	0	2	3	3	0	3	1	0	1	1	0	3	1	11	2	1	3	4	2	9
17 C(5)	-	7	10	5	4	3	5	6	7	8	9	3	7	4	3	2	-1	0	-2	1	0	1	2	0	1	3	3	4	7	4	11	3	5	2	1	3	6
18 C(6)	-	6	9	5	3	2	5	5	6	7	8	2	6	4	2	3	2	-1	0	-2	1	3	1	1	1	0	0	0	1	3	5	1	5	0	0	0	9
19 C(7)	-	7	8	4	2	4	6	6	5	7	8	3	5	3	-1	2	3	2	-1	0	0	2	1	1	0	2	2	1	3	8	0	1	4	1	1	2	7
20 C(8)	-	6	7	4	-1	4	6	7	3	5	7	3	4	3	3	4	4	4	3	-1	0	-1	3	1	2	2	3	0	4	11	4	0	4	0	2	2	5
21 C(9)	-	7	6	4	2	5	7	8	3	6	7	5	3	3	3	4	5	5	4	3	-1	0	-2	1	1	1	-1	0	2	10	4	1	0	0	1	2	1
22 C(10)	-	7	5	5	3	6	8	8	2	5	6	5	2	4	4	6	7	6	5	4	3	-1	0	0	1	2	0	5	2	8	1	5	-1	1	0	1	0
23 C(11)	-	8	5	5	4	8	9	10	3	5	7	7	3	4	5	7	8	7	6	5	4	2	-1	0	-2	0	3	4	4	4	0	5	4	7	7	5	6
24 C(12)	-	9	6	4	5	8	10	11	4	7	8	7	5	4	5	6	7	7	7	5	4	3	2	-1	0	-2	1	2	1	5	2	2	3	8	10	8	10
25 C(13)	-	10	8	3	5	8	9	10	5	7	9	7	5	2	4	5	6	7	6	4	4	2	3	2	-1	0	0	2	1	7	1	1	4	1	0	1	3
26 C(14)	-	9	8	2	4	6	8	9	4	7	8	6	5	-1	3	4	5	5	4	3	3	-1	2	3	2	-1	0	2	1	7	1	1	2	0	1	0	0
27 C(15)	-	5	9	6	3	-1	4	4	6	6	7	-1	6	5	4	4	4	2	-2	3	3	5	5	7	7	7	5	0	3	0	4	3	2	2	2	1	12
28 C(16)	-	3	8	8	4	3	2	2	6	5	6	-1	5	7	6	6	6	5	4	5	4	6	6	7	8	8	7	2	0	-1	-5	2	1	6	4	4	21
29 C(17)	-	-2	7	8	4	4	3	3	6	4	4	2	4	7	6	7	7	6	5	5	5	6	6	7	8	8	7	4	-2	0	2	2	10	4	5	2	17
30 C(18)	-	3	9	9	5	3	-1	-1	8	6	7	2	7	8	7	7	6	5	5	6	6	7	8	9	10	9	8	3	-2	2	0	3	5	2	0	0	24
31 C(19)	-	4	11	11	7	5	3	-1	10	8	8	5	9	10	9	9	8	7	6	8	8	9	10	11	12	11	10	5	4	5	2	0	0	1	5	5	10
32 C(20)	-	6	4	6	3	6	8	8	-1	4	5	5	-1	5	5	7	7	7	5	4	3	3	-2	2	4	4	4	5	6	5	7	9	0	1	1	0	2
33 C(21)	-	5	3	8	4	6	7	7	3	2	2	5	-1	7	7	8	9	8	7	6	5	5	4	5	6	7	6	6	5	4	7	9	2	0	-2	-1	0
34 C(22)	-	6	-2	9	5	8	9	9	3	3	3	7	2	8	8	10	10	9	8	7	6	6	4	5	6	7	7	7	7	6	8	10	3	-2	0	2	2
35 C(23)	-	4	3	9	5	7	7	7	4	-1	-1	5	2	8	8	9	9	8	7	7	5	6	5	5	7	7	7	6	5	4	6	8	4	-2	3	0	2
36 C(24)	-	4	4	11	7	8	7	7	6	3	-1	7	5	10	10	11	11	10	9	9	8	8	7	8	9	10	9	8	6	5	7	8	6	4	4	2	0

==

Remarks

- Upper Triangle Entries Represent /Del(A,B)/*1000 Values

- Lower Triangle Entries Represent Distances (A-B) Angstrom

- Negative Entries Indicate Bonded Atoms

Analysis of Bond Distance and Angle Values - Identification of Chiral Center(s) and Their (R/S)-Configuration (Cahn-Ingold-Prelog)

The Following Tests are done. Faults are Marked Under Note

-- V : Valency Check Fault for H, C

-- S : Bond Too Short

-- A : Unusual Bond Angle Values (PLEASE CHECK)

*** PLEASE NOTE: R/S ASSIGNMENTS ARE TENTATIVE *** (CIP Special rules NOT Implemented)

*** See Angew.Chem.Intern. Ed. Eng.,(1966),5,385 & (1982),21,567 for Authoritative Details for Special Cases

			=A.N.G.L.E.S=				=B.O.N.D.S=						
Flag	Label	- Connected to (May be Incomplete for Polymeric Structures)	nra	min	max	Aver	min	max	nrb	tnr	Hyb	RS	Note
d	S(1)	- C(17) H(1)	1	101	101	101.1	1.038	1.810	2	43			
d	S(2)	- C(22) H(2)	1	101	101	101.0	1.087	1.810	2	43			
d	O(1)	- C(1)	0	0	0	0.0	1.217	1.217	1	35	sp2		
d	O(2)	- C(8)	0	0	0	0.0	1.221	1.221	1	37	sp2		
d	O(3)	- C(15)	0	0	0	0.0	1.229	1.229	1	39	sp2		
d	O(4)	- C(18)	0	0	0	0.0	1.201	1.201	1	41	sp2		
d	O(5)	- C(18) C(19)	1	116	116	115.7	1.324	1.446	2	42	sp3		
d	O(6)	- C(20)	0	0	0	0.0	1.228	1.228	1	39	sp2		
d	O(7)	- C(23)	0	0	0	0.0	1.209	1.209	1	41	sp2		
d	O(8)	- C(23) C(24)	1	116	116	115.7	1.322	1.450	2	42	sp3		
d	N(1)	- C(15) C(16) H(3)	3	119	122	120.0	0.880	1.463	3	33	sp2		
d	N(2)	- C(20) C(21) H(4)	3	120	120	120.0	0.880	1.453	3	33	sp2		
d	C(1)	- O(1) C(2) C(14)	3	118	121	120.0	1.217	1.494	3	24	sp2		
d	C(2)	- C(1) C(3) C(7)	3	118	121	120.0	1.393	1.494	3	18	sp2		
d	C(3)	- C(2) C(4) H(5)	3	120	120	120.0	0.950	1.393	3	14	sp2		
d	C(4)	- C(3) C(5) H(6)	3	119	120	120.0	0.950	1.386	3	12	sp2		
d	C(5)	- C(4) C(6) H(7)	3	119	122	120.0	0.950	1.396	3	17	sp2		
d	C(6)	- C(5) C(7) C(15)	3	115	126	120.0	1.396	1.504	3	20	sp2		
d	C(7)	- C(2) C(6) C(8)	3	119	121	119.9	1.401	1.500	3	19	sp2		
d	C(8)	- O(2) C(7) C(9)	3	118	122	120.0	1.221	1.500	3	26	sp2		
d	C(9)	- C(8) C(10) C(14)	3	119	121	120.0	1.401	1.492	3	19	sp2		
d	C(10)	- C(9) C(11) C(20)	3	117	123	119.9	1.395	1.511	3	20	sp2		
d	C(11)	- C(10) C(12) H(8)	3	120	121	120.0	0.950	1.395	3	17	sp2		
d	C(12)	- C(11) C(13) H(9)	3	120	120	120.0	0.950	1.390	3	12	sp2		
d	C(13)	- C(12) C(14) H(10)	3	120	120	120.0	0.950	1.396	3	14	sp2		
d	C(14)	- C(1) C(9) C(13)	3	119	121	120.0	1.396	1.488	3	18	sp2		
d	C(15)	- O(3) N(1) C(6)	3	116	123	119.9	1.229	1.504	3	28	sp2		
d	C(16)	- N(1) C(17) C(18) H(11)	6	109	110	109.5	1.000	1.526	4	22	sp3	R	
d	C(17)	- S(1) C(16) H(12) H(13)	6	108	114	109.4	0.990	1.810	4	32	sp3		
d	C(18)	- O(4) O(5) C(16)	3	111	125	120.0	1.201	1.526	3	31	sp2		
d	C(19)	- O(5) H(14) H(15) H(16)	6	109	109	109.5	0.980	1.446	4	30	sp3		
d	C(20)	- O(6) N(2) C(10)	3	116	124	120.0	1.228	1.511	3	28	sp2		
d	C(21)	- N(2) C(22) C(23) H(17)	6	108	113	109.4	1.000	1.527	4	22	sp3	R	
d	C(22)	- S(2) C(21) H(18) H(19)	6	108	114	109.4	0.990	1.810	4	32	sp3		

d	C(23)	- O(7)	O(8)	C(21)	-------	-------	-------	-------	-------	-------	3	111	125	120.0	1.209	1.514	3	31	sp2
d	C(24)	- O(8)	H(20)	H(21)	H(22)	-------	-------	-------	-------	-------	6	109	109	109.5	0.980	1.450	4	30	sp3
d	H(1)	- S(1)	-------	-------	-------	-------	-------	-------	-------	-------	0	0	0	0.0	1.038	1.038	1	8	
d	H(2)	- S(2)	-------	-------	-------	-------	-------	-------	-------	-------	0	0	0	0.0	1.087	1.087	1	8	
d	H(3)	- N(1)	-------	-------	-------	-------	-------	-------	-------	-------	0	0	0	0.0	0.880	0.880	1	7	
d	H(4)	- N(2)	-------	-------	-------	-------	-------	-------	-------	-------	0	0	0	0.0	0.880	0.880	1	7	
d	H(5)	- C(3)	-------	-------	-------	-------	-------	-------	-------	-------	0	0	0	0.0	0.950	0.950	1	2	
d	H(6)	- C(4)	-------	-------	-------	-------	-------	-------	-------	-------	0	0	0	0.0	0.950	0.950	1	1	
d	H(7)	- C(5)	-------	-------	-------	-------	-------	-------	-------	-------	0	0	0	0.0	0.950	0.950	1	3	
d	H(8)	- C(11)	-------	-------	-------	-------	-------	-------	-------	-------	0	0	0	0.0	0.950	0.950	1	3	
d	H(9)	- C(12)	-------	-------	-------	-------	-------	-------	-------	-------	0	0	0	0.0	0.950	0.950	1	1	
d	H(10)	- C(13)	-------	-------	-------	-------	-------	-------	-------	-------	0	0	0	0.0	0.950	0.950	1	2	
d	H(11)	- C(16)	-------	-------	-------	-------	-------	-------	-------	-------	0	0	0	0.0	1.000	1.000	1	4	
d	H(12)	- C(17)	-------	-------	-------	-------	-------	-------	-------	-------	0	0	0	0.0	0.990	0.990	1	6	
d	H(13)	- C(17)	-------	-------	-------	-------	-------	-------	-------	-------	0	0	0	0.0	0.990	0.990	1	6	
d	H(14)	- C(19)	-------	-------	-------	-------	-------	-------	-------	-------	0	0	0	0.0	0.980	0.980	1	5	
d	H(15)	- C(19)	-------	-------	-------	-------	-------	-------	-------	-------	0	0	0	0.0	0.980	0.980	1	5	
d	H(16)	- C(19)	-------	-------	-------	-------	-------	-------	-------	-------	0	0	0	0.0	0.980	0.980	1	5	
d	H(17)	- C(21)	-------	-------	-------	-------	-------	-------	-------	-------	0	0	0	0.0	1.000	1.000	1	4	
d	H(18)	- C(22)	-------	-------	-------	-------	-------	-------	-------	-------	0	0	0	0.0	0.990	0.990	1	6	
d	H(19)	- C(22)	-------	-------	-------	-------	-------	-------	-------	-------	0	0	0	0.0	0.990	0.990	1	6	
d	H(20)	- C(24)	-------	-------	-------	-------	-------	-------	-------	-------	0	0	0	0.0	0.980	0.980	1	5	
d	H(21)	- C(24)	-------	-------	-------	-------	-------	-------	-------	-------	0	0	0	0.0	0.980	0.980	1	5	
d	H(22)	- C(24)	-------	-------	-------	-------	-------	-------	-------	-------	0	0	0	0.0	0.980	0.980	1	5	

Analysis of the IntraMolecular Geometry in Terms of Unique Molecule(s)/Ions, with Bond Criterium: d(i-j) < R(i) + R(j) + Tol

-- Tol = 0.40 Ang. for Normal Bonds + 0.70 for (Earth)alkali-NonMetal Contacts and adjusted by -.40 Ang. for Metal-Metal Distances
-- The Bond Distance and Angle su's have been Incremented to Include the Effect of the Unit-cell su.
(Rel.Error in Dist. 0.0000 Ang. , Abs. Angle Error 0.000 Deg.)
-- Bonds below with '>' or '<' Substituted for '-' have Distances that Deviate from Expected Values(Based on the hybridisations).

Bond Lengths (Angstrom). - (Bonds are ordered on the first label, left to right and top to bottom) - su in last digit in ().

S(1) - C(17)	1.8098(19)	S(2) - C(22)	1.810(2)	O(1) - C(1)	1.217(2)	O(2) - C(8)	1.221(2)
O(3) - C(15)	1.229(2)	O(4) - C(18)	1.201(3)	O(5) - C(18)	1.324(2)	O(5) - C(19)	1.446(3)
O(6) - C(20)	1.228(2)	O(7) - C(23)	1.209(2)	O(8) - C(23)	1.322(2)	O(8) - C(24)	1.450(3)
N(1) - C(15)	1.339(2)	N(1) - C(16)	1.463(2)	N(2) - C(20)	1.346(2)	N(2) - C(21)	1.453(2)
C(1) > C(2)	1.494(3)	C(1) > C(14)	1.488(3)	C(2) - C(3)	1.393(3)	C(2) - C(7)	1.401(3)
C(3) - C(4)	1.386(3)	C(4) - C(5)	1.384(3)	C(5) - C(6)	1.396(3)	C(6) - C(7)	1.404(3)
C(6) > C(15)	1.504(3)	C(7) > C(8)	1.500(2)	C(8) > C(9)	1.492(3)	C(9) - C(10)	1.401(2)
C(9) - C(14)	1.403(2)	C(10) - C(11)	1.395(3)	C(10) > C(20)	1.511(3)	C(11) - C(12)	1.390(3)
C(12) - C(13)	1.380(3)	C(13) - C(14)	1.396(3)	C(16) - C(17)	1.524(3)	C(16) - C(18)	1.526(3)
C(21) - C(22)	1.527(2)	C(21) - C(23)	1.514(3)				

S(1) - H(1)	1.04	S(2) - H(2)	1.09	N(1) - H(3)	0.88	N(2) - H(4)	0.88
C(3) - H(5)	0.95	C(4) - H(6)	0.95	C(5) - H(7)	0.95	C(11) - H(8)	0.95
C(12) - H(9)	0.95	C(13) - H(10)	0.95	C(16) - H(11)	1.00	C(17) - H(12)	0.99
C(17) - H(13)	0.99	C(19) - H(14)	0.98	C(19) - H(15)	0.98	C(19) - H(16)	0.98
C(21) - H(17)	1.00	C(22) - H(18)	0.99	C(22) - H(19)	0.99	C(24) - H(20)	0.98
C(24) - H(21)	0.98	C(24) - H(22)	0.98				

Bond/Valence Angles (Degrees) - (Angles are ordered on the middle label, left to right and top to bottom) - su in last digit in ().

C(18) - O(5) - C(19)	115.67(17)	C(23) - O(8) - C(24)	115.67(16)	C(15) - N(1) - C(16)	121.67(16)
C(20) - N(2) - C(21)	120.32(15)	O(1) - C(1) - C(2)	120.49(18)	O(1) - C(1) - C(14)	121.44(18)
C(2) - C(1) - C(14)	118.07(15)	C(1) - C(2) - C(3)	118.43(16)	C(1) - C(2) - C(7)	120.84(16)
C(3) - C(2) - C(7)	120.73(17)	C(2) - C(3) - C(4)	120.04(18)	C(3) - C(4) - C(5)	119.45(19)
C(4) - C(5) - C(6)	121.52(18)	C(5) - C(6) - C(7)	119.07(17)	C(5) - C(6) - C(15)	115.26(16)
C(7) - C(6) - C(15)	125.67(16)	C(2) - C(7) - C(6)	119.07(16)	C(2) - C(7) - C(8)	120.04(16)
C(6) - C(7) - C(8)	120.58(16)	O(2) - C(8) - C(7)	120.44(17)	O(2) - C(8) - C(9)	121.65(16)
C(7) - C(8) - C(9)	117.89(14)	C(8) - C(9) - C(10)	120.08(15)	C(8) - C(9) - C(14)	120.62(16)
C(10) - C(9) - C(14)	119.30(17)	C(9) - C(10) - C(11)	119.42(16)	C(9) - C(10) - C(20)	122.98(17)
C(11) - C(10) - C(20)	117.43(16)	C(10) - C(11) - C(12)	120.59(17)	C(11) - C(12) - C(13)	120.21(18)
C(12) - C(13) - C(14)	119.85(17)	C(1) - C(14) - C(9)	120.72(17)	C(1) - C(14) - C(13)	118.84(16)
C(9) - C(14) - C(13)	120.39(17)	O(3) - C(15) - N(1)	123.37(17)	O(3) - C(15) - C(6)	119.81(17)
N(1) - C(15) - C(6)	116.49(17)	N(1) - C(16) - C(17)	108.89(15)	N(1) - C(16) - C(18)	109.65(14)
C(17) - C(16) - C(18)	109.82(15)	S(1) - C(17) - C(16)	114.02(13)	O(4) - C(18) - O(5)	124.98(18)
O(4) - C(18) - C(16)	124.08(18)	O(5) - C(18) - C(16)	110.93(16)	O(6) - C(20) - N(2)	123.63(16)
O(6) - C(20) - C(10)	120.28(16)	N(2) - C(20) - C(10)	116.00(16)	N(2) - C(21) - C(22)	113.14(15)
N(2) - C(21) - C(23)	108.67(15)	C(22) - C(21) - C(23)	111.03(15)	S(2) - C(22) - C(21)	114.25(14)
O(7) - C(23) - O(8)	125.02(17)	O(7) - C(23) - C(21)	124.31(16)	O(8) - C(23) - C(21)	110.66(15)

C(17) - S(1) - H(1)	101	C(22) - S(2) - H(2)	101	C(15) - N(1) - H(3)	119
C(16) - N(1) - H(3)	119	C(20) - N(2) - H(4)	120	C(21) - N(2) - H(4)	120
C(2) - C(3) - H(5)	120	C(4) - C(3) - H(5)	120	C(3) - C(4) - H(6)	120
C(5) - C(4) - H(6)	120	C(4) - C(5) - H(7)	119	C(6) - C(5) - H(7)	119
C(10) - C(11) - H(8)	120	C(12) - C(11) - H(8)	120	C(11) - C(12) - H(9)	120
C(13) - C(12) - H(9)	120	C(12) - C(13) - H(10)	120	C(14) - C(13) - H(10)	120
N(1) - C(16) - H(11)	109	C(17) - C(16) - H(11)	109	C(18) - C(16) - H(11)	109
S(1) - C(17) - H(12)	109	S(1) - C(17) - H(13)	109	C(16) - C(17) - H(12)	109
C(16) - C(17) - H(13)	109	H(12) - C(17) - H(13)	108	O(5) - C(19) - H(14)	109
O(5) - C(19) - H(15)	109	O(5) - C(19) - H(16)	109	H(14) - C(19) - H(15)	109
H(14) - C(19) - H(16)	109	H(15) - C(19) - H(16)	109	N(2) - C(21) - H(17)	108
C(22) - C(21) - H(17)	108	C(23) - C(21) - H(17)	108	S(2) - C(22) - H(18)	109
S(2) - C(22) - H(19)	109	C(21) - C(22) - H(18)	109	C(21) - C(22) - H(19)	109
H(18) - C(22) - H(19)	108	O(8) - C(24) - H(20)	109	O(8) - C(24) - H(21)	109
O(8) - C(24) - H(22)	109	H(20) - C(24) - H(21)	109	H(20) - C(24) - H(22)	109
H(21) - C(24) - H(22)	109				

Torsion/Dihedral Angles (Deg.) - Klyne & Prelog Convention (Dunitz, p241) - (Excl. Minor Disorder & Embedded Bond Angl. > 160. Deg.)

C(19) O(5) C(18) O(4)	-2.8(3)	C(19) O(5) C(18) C(16)	176.23(17)	C(24) O(8) C(23) O(7)	-2.8(3)
C(24) O(8) C(23) C(21)	176.02(16)	C(16) N(1) C(15) O(3)	6.6(3)	C(16) N(1) C(15) C(6)	179.86(15)
C(15) N(1) C(16) C(17)	144.75(16)	C(15) N(1) C(16) C(18)	-95.1(2)	C(21) N(2) C(20) O(6)	-7.6(3)
C(21) N(2) C(20) C(10)	175.97(15)	C(20) N(2) C(21) C(22)	67.0(2)	C(20) N(2) C(21) C(23)	-169.23(16)
O(1) C(1) C(2) C(3)	-4.6(3)	O(1) C(1) C(2) C(7)	175.51(19)	C(14) C(1) C(2) C(3)	176.33(17)
C(14) C(1) C(2) C(7)	-3.6(3)	O(1) C(1) C(14) C(9)	-168.63(19)	O(1) C(1) C(14) C(13)	8.6(3)
C(2) C(1) C(14) C(9)	10.4(3)	C(2) C(1) C(14) C(13)	-172.31(17)	C(1) C(2) C(3) C(4)	179.30(19)
C(7) C(2) C(3) C(4)	-0.8(3)	C(1) C(2) C(7) C(6)	177.63(17)	C(1) C(2) C(7) C(8)	-8.8(3)
C(3) C(2) C(7) C(6)	-2.3(3)	C(3) C(2) C(7) C(8)	171.36(18)	C(2) C(3) C(4) C(5)	2.2(3)
C(3) C(4) C(5) C(6)	-0.5(3)	C(4) C(5) C(6) C(7)	-2.6(3)	C(4) C(5) C(6) C(15)	177.53(18)
C(5) C(6) C(7) C(2)	3.9(3)	C(5) C(6) C(7) C(8)	-169.67(17)	C(15) C(6) C(7) C(2)	-176.25(18)
C(15) C(6) C(7) C(8)	10.2(3)	C(5) C(6) C(15) O(3)	54.6(2)	C(5) C(6) C(15) N(1)	-118.94(19)
C(7) C(6) C(15) O(3)	-125.3(2)	C(7) C(6) C(15) N(1)	61.2(2)	C(2) C(7) C(8) O(2)	-163.79(18)
C(2) C(7) C(8) C(9)	14.4(3)	C(6) C(7) C(8) O(2)	9.7(3)	C(6) C(7) C(8) C(9)	-172.07(17)
O(2) C(8) C(9) C(10)	-8.6(3)	O(2) C(8) C(9) C(14)	170.55(18)	C(7) C(8) C(9) C(10)	173.18(17)
C(7) C(8) C(9) C(14)	-7.6(3)	C(8) C(9) C(10) C(11)	-175.18(17)	C(8) C(9) C(10) C(20)	9.7(3)
C(14) C(9) C(10) C(11)	5.6(3)	C(14) C(9) C(10) C(20)	-169.47(17)	C(8) C(9) C(14) C(1)	-4.6(3)
C(8) C(9) C(14) C(13)	178.14(17)	C(10) C(9) C(14) C(1)	174.56(17)	C(10) C(9) C(14) C(13)	-2.7(3)
C(9) C(10) C(11) C(12)	-4.3(3)	C(20) C(10) C(11) C(12)	171.03(17)	C(9) C(10) C(20) O(6)	81.7(2)
C(9) C(10) C(20) N(2)	-101.8(2)	C(11) C(10) C(20) O(6)	-93.5(2)	C(11) C(10) C(20) N(2)	83.0(2)
C(10) C(11) C(12) C(13)	0.0(3)	C(11) C(12) C(13) C(14)	3.0(3)	C(12) C(13) C(14) C(1)	-178.96(18)
C(12) C(13) C(14) C(9)	-1.7(3)	N(1) C(16) C(17) S(1)	172.76(12)	C(18) C(16) C(17) S(1)	52.68(18)
N(1) C(16) C(18) O(4)	-51.3(3)	N(1) C(16) C(18) O(5)	129.67(16)	C(17) C(16) C(18) O(4)	68.3(2)
C(17) C(16) C(18) O(5)	-110.71(18)	N(2) C(21) C(22) S(2)	69.97(19)	C(23) C(21) C(22) S(2)	-52.52(19)
N(2) C(21) C(23) O(7)	0.3(3)	N(2) C(21) C(23) O(8)	-178.50(15)	C(22) C(21) C(23) O(7)	125.4(2)
C(22) C(21) C(23) O(8)	-53.5(2)				
H(1) S(1) C(17) C(16)	68	H(1) S(1) C(17) H(12)	-171	H(1) S(1) C(17) H(13)	-54

H(2)	S(2)	C(22)	C(21)	-65	H(2)	S(2)	C(22)	H(18)	57	H(2)	S(2)	C(22)	H(19)	174
C(18)	O(5)	C(19)	H(14)	-72	C(18)	O(5)	C(19)	H(15)	168	C(18)	O(5)	C(19)	H(16)	48
C(23)	O(8)	C(24)	H(20)	76	C(23)	O(8)	C(24)	H(21)	-44	C(23)	O(8)	C(24)	H(22)	-164
H(3)	N(1)	C(15)	O(3)	-173	H(3)	N(1)	C(15)	C(6)	0	C(15)	N(1)	C(16)	H(11)	25
H(3)	N(1)	C(16)	C(17)	-35	H(3)	N(1)	C(16)	C(18)	85	H(3)	N(1)	C(16)	H(11)	-155
H(4)	N(2)	C(20)	O(6)	172	H(4)	N(2)	C(20)	C(10)	-4	C(20)	N(2)	C(21)	H(17)	-52
H(4)	N(2)	C(21)	C(22)	-113	H(4)	N(2)	C(21)	C(23)	11	H(4)	N(2)	C(21)	H(17)	128
C(1)	C(2)	C(3)	H(5)	-1	C(7)	C(2)	C(3)	H(5)	179	C(2)	C(3)	C(4)	H(6)	-178
H(5)	C(3)	C(4)	C(5)	-178	H(5)	C(3)	C(4)	H(6)	2	C(3)	C(4)	C(5)	H(7)	180
H(6)	C(4)	C(5)	C(6)	180	H(6)	C(4)	C(5)	H(7)	0	H(7)	C(5)	C(6)	C(7)	177
H(7)	C(5)	C(6)	C(15)	-2	C(9)	C(10)	C(11)	H(8)	176	C(20)	C(10)	C(11)	H(8)	-9
C(10)	C(11)	C(12)	H(9)	-180	H(8)	C(11)	C(12)	C(13)	180	H(8)	C(11)	C(12)	H(9)	0
C(11)	C(12)	C(13)	H(10)	-177	H(9)	C(12)	C(13)	C(14)	-177	H(9)	C(12)	C(13)	H(10)	3
H(10)	C(13)	C(14)	C(1)	1	H(10)	C(13)	C(14)	C(9)	178	N(1)	C(16)	C(17)	H(12)	51
N(1)	C(16)	C(17)	H(13)	-66	C(18)	C(16)	C(17)	H(12)	-69	C(18)	C(16)	C(17)	H(13)	174
H(11)	C(16)	C(17)	S(1)	-68	H(11)	C(16)	C(17)	H(12)	171	H(11)	C(16)	C(17)	H(13)	54
H(11)	C(16)	C(18)	O(4)	-171	H(11)	C(16)	C(18)	O(5)	10	N(2)	C(21)	C(22)	H(18)	-52
N(2)	C(21)	C(22)	H(19)	-168	C(23)	C(21)	C(22)	H(18)	-174	C(23)	C(21)	C(22)	H(19)	69
H(17)	C(21)	C(22)	S(2)	-171	H(17)	C(21)	C(22)	H(18)	68	H(17)	C(21)	C(22)	H(19)	-49
H(17)	C(21)	C(23)	O(7)	-117	H(17)	C(21)	C(23)	O(8)	65					

```
====================================================================================================================================
Statistics of Bond Length per Bond Type (NOTE: A Indicates 10 Occurrences, B Indicates 11, Etc. and * more than 35)
====================================================================================================================================

Bond Type   Nr    B.P.  d(min)  d(max)   d(av) Sumrad Note  0.0       0.5       1.0       1.5       2.0       2.5       3.0 Angstrom
----------------------------------------------------------   I-I-I-I-I-I-I-I-I-I-I-I-I-I-I-I-I-I-I-I-I-I-I-I-I-I-I-I-I-I-I-I-I-I-I-I
 S  --  C    2  0.0020  1.8098  1.8100  1.8099   1.72        ....................................2..................................

 O  --  C3   6  0.0022  1.2010  1.2290  1.2175   1.36        ........................42.............................................

 O  --  C3   2  0.0020  1.3220  1.3240  1.3230   1.36        ..........................2............................................

 O  --  C4   2  0.0030  1.4460  1.4500  1.4480   1.36        .............................2.........................................

 N3 --  C3   2  0.0020  1.3390  1.3460  1.3425   1.36        ...........................2...........................................

 N3 --  C4   2  0.0020  1.4530  1.4630  1.4580   1.36        .............................2.........................................

 C3 --  C3  18  0.0028  1.3800  1.5110  1.4288   1.36        ............................C.6........................................

 C4 --  C4   2  0.0025  1.5240  1.5270  1.5255   1.36        ..............................11.......................................

 C4 --  C3   2  0.0030  1.5140  1.5260  1.5200   1.36        ..............................11.......................................

 S  --  H    2          1.0400  1.0900  1.0650   1.39        .....................11................................................

 N  --  H    2          0.8800  0.8800  0.8800   1.03        ..................2....................................................

 C  --  H   18          0.9500  1.0000  0.9744   1.03        ...................6C..................................................
```

Selected Bond Lengths (Angstrom) - see M.F.C. Ladd & R.A. Palmer, Structure Determination by X-Ray Crystallography (1985)

Formal single bonds

C4-C4 1.54	C4-C3 1.52	C4-C2 1.46	C4-N3 1.47	C4-N2 1.47	C4-O2 1.43	C3-C3 1.46	C3-C2 1.45	C3-N3 1.40	C3-N2 1.40
C3-O2 1.36	C2-C2 1.38	C2-N3 1.33	C2-N2 1.33	C2-O2 1.36	N3-N3 1.45	N3-N2 1.45	N3-O2 1.36	N2-N2 1.45	N2-O2 1.41

Formal double bonds

C3-C3 1.34	C3-C2 1.31	C3-N2 1.32	C3-O1 1.22	C2-C2 1.28	C2-N2 1.32	C2-O1 1.16	N3-O1 1.24	N2-N2 1.25	N2-O1 1.21

Formal triple bonds		Aromatic bonds		
C2-C2 1.20	C2-N1 1.16	C3-C3 1.40	C2-N2 1.34	N2-N2 1.35

The notation in the table indicates the connectivity of the atoms

For more detailed standard bond distance tabulations see: J. Chem. Soc. Perkin II, (1987), S1-S19;
J. Chem. Soc. Dalton Trans. (1989), S1 - S83 or International Tables C, (1992), 707-791.

```
=====================================================================================================================================
Automatic Search for Rings (3 to 24-Membered) and Planes Determined by 4 or More Connected Atoms (with Deviation < 0.10 Ang.)
-------------------------------------------------------------------------------------------------------------------------------------


Least-Squares Planes - P*X+Q*Y+R*Z=S   ::  First Line Orthogonal(XO,YO,ZO), Second Line Fractional(X,Y,Z)
===================================         Ring/Plan/Resd/Lspl N Indicates that the Ring/Plane/Residue Involves N Atoms
                                            Sigref - R.M.S-Error of the Contributing Atoms
  The Deviation D of an Atom with           SigpIn - Sqrt(Sum(j=1:N)(D(j)**2/(N-3))
                                            Chisq  - Chi-Squared = Sum(j=1:N)(D(j)**2)/Sigref**2
  Fractional Coordinates X,Y,Z may be       Pl.Hyp. - Result of the Chi.Sq. Test for Planarity (See Stout & Jensen, p424)

  Calculated via Substitution in
                                            **** - Atoms Deviating by More Than 1.5 Angstrom and Hydrogen Atoms are NOT Listed
  D = P*X + Q*Y + R*Z - S (2nd Line)        Note - Weights : UNIT
                                                 - Deviations from planes are in Angstrom Units
                                                 - The Plane determining Atoms have been Marked #
                                                 - DISTANCES TO PLANES ROUNDED TO 3 DECIMALS !!(Use Graphical Interface for more)
-------------------------------------------------------------------------------------------------------------------------------------
Nr  1         P             Q             R             S     Sigref  0.002      SigpIn  0.081     Chisq     5996.7    Pl.Hyp.    P<5
-------------------------------------------------------------------------------------------------------------------------------------
Ring     0.7165(5)     0.5631(6)    -0.4117(7)     2.703(11)  #C(1)     0.062(2)  #C(2)     0.009(2)  #C(7)    -0.078(2)  #C(8)     0.077(2)
A  6      3.593(3)    10.523(11)   -10.473(18)     2.703(11)  #C(9)    -0.007(2)  #C(14)   -0.063(2)   S(2)    -0.794(1)   O(1)     0.220(2)
                                                               O(2)     0.297(1)   O(3)     0.434(2)   O(6)     1.436(1)   N(1)    -1.472(2)
                                                               N(2)    -0.795(2)   C(3)     0.047(2)   C(4)     0.015(2)   C(5)    -0.100(2)
                                                               C(6)    -0.176(2)   C(10)   -0.012(2)   C(11)   -0.192(2)   C(12)   -0.275(2)
                                                               C(13)   -0.180(2)   C(15)   -0.357(2)   C(20)    0.277(2)   C(21)   -0.634(2)
                                                               C(22)    0.124(2)
-------------------------------------------------------------------------------------------------------------------------------------
Nr  2         P             Q             R             S     Sigref  0.002      SigpIn  0.019     Chisq      302.0    Pl.Hyp.    P<5
-------------------------------------------------------------------------------------------------------------------------------------
Ring     0.7363(6)     0.5851(7)    -0.3399(8)     3.728(11)  #C(2)    -0.002(2)  #C(3)    -0.014(2)  #C(4)     0.013(2)  #C(5)     0.005(2)
A  6      3.693(3)    10.933(13)    -8.645(19)     3.728(11)  #C(6)    -0.020(2)  #C(7)     0.019(2)   S(2)    -0.363(1)   O(1)     0.049(2)
                                                               O(2)     0.539(1)   O(3)     0.757(2)   O(8)    -1.327(1)   N(1)    -1.158(2)
                                                               N(2)    -0.486(2)   C(1)    -0.015(2)   C(8)     0.226(2)   C(9)     0.078(2)
                                                               C(10)    0.120(2)   C(11)   -0.121(2)   C(12)   -0.312(2)   C(13)   -0.264(2)
                                                               C(14)   -0.087(2)   C(15)   -0.085(2)   C(16)   -1.337(2)   C(20)    0.521(2)
                                                               C(21)   -0.214(2)   C(22)    0.573(2)
-------------------------------------------------------------------------------------------------------------------------------------
Nr  3         P             Q             R             S     Sigref  0.002      SigpIn  0.028     Chisq      676.6    Pl.Hyp.    P<5
-------------------------------------------------------------------------------------------------------------------------------------
Ring     0.6457(6)     0.6009(6)    -0.4711(7)     2.200(12)  #C(9)    -0.025(2)  #C(10)    0.031(2)  #C(11)   -0.011(2)  #C(12)   -0.016(2)
A  6      3.238(3)    11.230(12)   -11.982(17)     2.200(12)  #C(13)    0.021(2)  #C(14)   -0.001(2)   S(2)    -0.728(1)   O(1)     0.289(2)
                                                               O(2)     0.059(1)   O(3)    -0.126(2)   O(6)     1.374(1)   N(2)    -0.857(2)
                                                               C(1)     0.063(2)   C(2)    -0.137(2)   C(3)    -0.157(2)   C(4)    -0.326(2)
                                                               C(5)    -0.520(2)   C(6)    -0.541(2)   C(7)    -0.305(2)   C(8)    -0.090(2)
                                                               C(15)   -0.820(2)   C(20)    0.243(2)   C(21)   -0.777(2)   C(22)    0.050(2)
-------------------------------------------------------------------------------------------------------------------------------------
Nr  4         P             Q             R             S     Sigref  0.002      SigpIn  0.076     Chisq    11352.5    Pl.Hyp.    P<5
-------------------------------------------------------------------------------------------------------------------------------------
Ring     0.7327(3)     0.5659(3)    -0.3781(5)      3.153(8)  #C(1)     0.017(2)  #C(2)     0.007(2)  #C(3)     0.035(2)  #C(4)     0.041(2)
```

```
==========================================================================================================================================
A 10    3.6741(13)      10.575(6)     -9.618(14)       3.153(8)  #C(5)     -0.025(2)  #C(6)      -0.090(2)  #C(7)     -0.031(2)  #C(8)      0.135(2)
                                                                 #C(9)      0.009(2)  #C(14)     -0.097(2)  S(2)      -0.665(1)  O(1)       0.132(2)
                                                                  O(2)      0.397(1)  O(3)        0.605(2)  N(1)      -1.318(2)  N(2)      -0.693(1)
                                                                  C(10)     0.013(2)  C(11)      -0.207(2)  C(12)     -0.339(2)  C(13)     -0.253(2)
                                                                  C(15)    -0.216(2)  C(20)       0.352(2)  C(21)     -0.482(2)  C(22)      0.278(2)
------------------------------------------------------------------------------------------------------------------------------------------
Nr  5         P               Q              R              S    Sigref  0.002      Sigpln   0.086       Chisq    15051.6    Pl.Hyp.       P<5
------------------------------------------------------------------------------------------------------------------------------------------
Ring    0.6799(3)       0.5862(3)      -0.4407(5)      2.440(8)  #C(1)      0.112(2)  #C(2)      -0.019(2)  #C(7)     -0.144(2)  #C(8)      0.048(2)
A 10    3.4093(14)      10.954(6)     -11.209(13)      2.440(8)  #C(9)      0.042(2)  #C(10)      0.073(2)  #C(11)    -0.034(2)  #C(12)    -0.081(2)
                                                                 #C(13)    -0.021(2)  #C(14)      0.023(2)  S(2)      -0.679(1)  O(1)       0.301(2)
                                                                  O(2)      0.235(1)  O(3)        0.199(2)  O(6)       1.474(1)  N(2)      -0.758(2)
                                                                  C(3)     -0.015(2)  C(4)       -0.119(2)  C(5)      -0.272(2)  C(6)      -0.314(2)
                                                                  C(15)    -0.542(2)  C(20)       0.327(2)  C(21)     -0.634(2)  C(22)      0.165(2)
------------------------------------------------------------------------------------------------------------------------------------------
Nr  6         P               Q              R              S    Sigref  0.002      Sigpln   0.107       Chisq    35023.9    Pl.Hyp.       P<5
------------------------------------------------------------------------------------------------------------------------------------------
Ring    0.7059(2)       0.5785(2)      -0.4088(4)      2.768(7)  #C(1)      0.116(2)  #C(2)       0.045(2)  #C(3)      0.057(2)  #C(4)      0.007(2)
A 14    3.5398(9)       10.810(4)     -10.399(11)      2.768(7)  #C(5)     -0.101(2)  #C(6)      -0.151(2)  #C(7)     -0.035(2)  #C(8)      0.147(2)
                                                                 #C(9)      0.083(2)  #C(10)      0.104(2)  #C(11)    -0.059(2)  #C(12)    -0.150(2)
                                                                 #C(13)    -0.080(2)  #C(14)      0.019(2)  S(2)      -0.601(1)  O(1)       0.267(2)
                                                                  O(2)      0.374(1)  O(3)        0.454(2)  N(1)      -1.430(2)  N(2)      -0.662(2)
                                                                  C(15)    -0.327(2)  C(20)       0.402(2)  C(21)     -0.492(2)  C(22)      0.290(2)
------------------------------------------------------------------------------------------------------------------------------------------
Nr  7         P               Q              R              S    Sigref  0.002      Sigpln   0.847       Chisq   999999.9    Pl.Hyp.
------------------------------------------------------------------------------------------------------------------------------------------
Resd    0.8287(1)       0.5544(1)      -0.0774(1)    6.8520(19)  #S(1)     -0.582(1)  #S(2)       0.267(1)  #O(1)     -0.757(2)  #O(2)      1.057(1)
A 36    4.1556(3)     10.3594(19)       -1.969(3)    6.8520(19)  #O(3)      1.855(2)  #O(4)      -1.644(2)  #O(5)      0.349(2)  #O(6)      2.005(1)
                                                                 #O(7)     -1.582(1)  #O(8)       0.095(1)  #N(1)     -0.111(2)  #N(2)      0.026(2)
                                                                 #C(1)     -0.512(2)  #C(2)      -0.160(2)  #C(3)     -0.214(2)  #C(4)      0.120(2)
                                                                 #C(5)      0.463(2)  #C(6)       0.489(2)  #C(7)      0.214(2)  #C(8)      0.456(2)
                                                                 #C(9)     -0.023(2)  #C(10)      0.049(2)  #C(11)    -0.494(2)  #C(12)    -1.022(2)
                                                                 #C(13)    -1.014(2)  #C(14)     -0.532(2)  #C(15)     0.824(2)  #C(16)     0.095(2)
                                                                 #C(17)    -0.572(2)  #C(18)     -0.518(2)  #C(19)    -0.154(3)  #C(20)     0.788(2)
                                                                 #C(21)     0.643(2)  #C(22)      1.366(2)  #C(23)    -0.420(2)  #C(24)    -0.849(3)
------------------------------------------------------------------------------------------------------------------------------------------
Nr  8         P               Q              R              S    Sigref  0.002      Sigpln   0.032       Chisq      548.6    Pl.Hyp.       P<5
------------------------------------------------------------------------------------------------------------------------------------------
Plan    0.5602(8)      -0.3550(9)      -0.7484(5)   -12.329(14)  #O(4)      0.003(2)  #O(5)       0.030(2)  #C(16)    -0.020(2)  #C(18)     0.012(2)
A  5      2.809(4)     -6.634(17)     -19.037(14)   -12.329(14)  #C(19)    -0.025(3)  O(2)        1.241(1)  O(6)      -0.027(1)  N(1)       1.024(2)
                                                                  C(10)     0.565(2)  C(11)       0.307(2)  C(12)      1.199(2)  C(17)     -1.375(2)
                                                                  C(20)    -0.338(2)
------------------------------------------------------------------------------------------------------------------------------------------
Nr  9         P               Q              R              S    Sigref  0.002      Sigpln   0.040       Chisq     1538.8    Pl.Hyp.       P<5
------------------------------------------------------------------------------------------------------------------------------------------
Plan   -0.7111(6)       0.6914(7)      -0.1273(5)     4.664(13)  #O(7)     -0.019(1)  #O(8)      -0.027(1)  #N(2)      0.033(2)  #C(21)    -0.004(2)
A  6     -3.566(3)     12.921(12)      -3.238(14)     4.664(13)  #C(23)    -0.026(2)  #C(24)      0.044(3)  O(6)      -0.239(1)  C(9)      -1.386(2)
                                                                  C(10)    -0.195(2)  C(11)       1.027(2)  C(12)      1.073(2)  C(13)     -0.094(2)
```

					C(14)	-1.327(2)	C(20)	-0.160(2)	C(22)	1.152(2)		
Nr 10	P	Q	R	S	Sigref	0.002	Sigpln	0.014	Chisq	67.3	Pl.Hyp.	P<5
Plan	-0.185(2)	0.9708(5)	-0.1537(7)	3.93(2)	#N(1)	0.001(1)	#C(6)	-0.001(2)	#C(15)	0.001(2)	#C(16)	-0.001(2)
A 4	-0.925(12)	18.141(10)	-3.909(18)	3.93(2)	S(1)	0.756(1)	O(3)	-0.120(2)	C(2)	0.818(2)	C(3)	-0.311(2)
					C(4)	-1.265(2)	C(5)	-1.106(2)	C(7)	0.998(2)	C(17)	0.832(2)
					C(18)	-1.433(2)						
Nr 11	P	Q	R	S	Sigref	0.002	Sigpln	0.046	Chisq	692.0	Pl.Hyp.	P<5
Plan	-0.605(2)	0.7670(16)	-0.2138(8)	4.49(2)	#N(2)	0.026(2)	#C(10)	-0.022(2)	#C(20)	0.020(2)	#C(21)	-0.024(2)
A 4	-3.034(11)	14.33(3)	-5.44(2)	4.49(2)	O(6)	0.109(1)	O(7)	-0.409(1)	O(8)	-0.293(1)	C(9)	-1.191(2)
					C(11)	1.188(2)	C(12)	1.257(2)	C(13)	0.126(2)	C(14)	-1.104(2)
					C(22)	1.233(2)	C(23)	-0.272(2)	C(24)	-0.434(2)		
Nr 12	P	Q	R	S	Sigref	0.002	Sigpln	0.035	Chisq	2379.7	Pl.Hyp.	P<5
Plan	0.7309(3)	0.6016(4)	-0.3223(4)	4.007(6)	#O(1)	0.034(2)	#C(1)	-0.005(2)	#C(2)	0.007(2)	#C(3)	-0.036(2)
A 10	3.6651(14)	11.243(8)	-8.199(10)	4.007(6)	#C(4)	-0.013(2)	#C(5)	0.007(2)	#C(6)	0.014(2)	#C(7)	0.056(2)
					#C(14)	-0.043(2)	#C(15)	-0.022(2)	S(2)	-0.132(1)	O(2)	0.633(1)
					O(3)	0.821(2)	O(8)	-1.100(1)	N(1)	-1.075(2)	N(2)	-0.331(2)
					C(8)	0.297(2)	C(9)	0.151(2)	C(10)	0.223(2)	C(11)	-0.015(2)
					C(12)	-0.234(2)	C(13)	-0.217(2)	C(16)	-1.226(2)	C(20)	0.656(2)
					C(21)	-0.029(2)	C(22)	0.784(2)	C(23)	-1.328(2)		
Nr 13	P	Q	R	S	Sigref	0.002	Sigpln	0.011	Chisq	43.3	Pl.Hyp.	P<5
Plan	0.6827(7)	0.4741(9)	-0.5561(8)	0.044(15)	#O(2)	0.004(1)	#C(7)	0.003(2)	#C(8)	-0.010(2)	#C(9)	0.003(2)
A 4	3.424(4)	8.859(17)	-14.14(2)	0.044(15)	O(1)	0.834(2)	O(3)	0.090(2)	O(6)	0.950(1)	N(2)	-1.318(2)
					C(1)	0.473(2)	C(2)	0.327(2)	C(3)	0.518(2)	C(4)	0.405(2)
					C(5)	0.057(2)	C(6)	-0.175(2)	C(10)	-0.152(2)	C(11)	-0.240(2)
					C(12)	-0.085(2)	C(13)	0.158(2)	C(14)	0.186(2)	C(15)	-0.600(2)
					C(20)	-0.122(2)	C(21)	-1.406(2)	C(22)	-0.777(2)		
Nr 14	P	Q	R	S	Sigref	0.002	Sigpln	0.043	Chisq	2685.3	Pl.Hyp.	P<5
Plan	0.6541(4)	0.6071(4)	-0.4512(5)	2.494(9)	#C(1)	0.040(2)	#C(8)	-0.048(2)	#C(9)	-0.004(2)	#C(10)	0.063(2)
A 8	3.2799(19)	11.345(8)	-11.478(13)	2.494(9)	#C(11)	0.002(2)	#C(12)	-0.034(2)	#C(13)	-0.008(2)	#C(14)	-0.011(2)
					S(2)	-0.617(1)	O(1)	0.240(2)	O(2)	0.128(1)	O(3)	-0.028(2)
					O(6)	1.449(1)	N(2)	-0.776(2)	C(2)	-0.139(2)	C(3)	-0.170(2)
					C(4)	-0.321(2)	C(5)	-0.485(2)	C(6)	-0.493(2)	C(7)	-0.276(2)
					C(15)	-0.739(2)	C(20)	0.307(2)	C(21)	-0.664(2)	C(22)	0.170(2)
Nr 15	P	Q	R	S	Sigref	0.002	Sigpln	0.047	Chisq	2118.6	Pl.Hyp.	P<5
Plan	0.6508(3)	0.5608(10)	-0.5118(9)	1.31(2)	#O(2)	0.039(1)	#C(8)	-0.051(2)	#C(9)	0.016(2)	#C(10)	0.003(2)
A 6	3.2637(16)	10.480(18)	-13.02(2)	1.31(2)	#C(11)	-0.036(2)	#C(12)	0.029(2)	S(2)	-1.043(1)	O(1)	0.534(2)

					O(3)	-0.092(2)	O(6)	1.246(1)	N(2)	-1.004(2)	C(1)	0.248(2)
					C(2)	0.046(2)	C(3)	0.097(2)	C(4)	-0.072(2)	C(5)	-0.334(2)
					C(6)	-0.425(2)	C(7)	-0.191(2)	C(13)	0.134(2)	C(14)	0.110(2)
					C(15)	-0.772(2)	C(20)	0.137(2)	C(21)	-0.997(2)	C(22)	-0.232(2)

Nr 16	P	Q	R	S	Sigref	0.002	Sigpln	0.030	Chisq	268.6	Pl.Hyp.	P<5
Plan	0.6139(9)	0.5458(9)	-0.5704(8)	0.378(17)	#C(9)	0.009(2)	#C(10)	-0.026(2)	#C(11)	0.009(2)	#C(20)	0.008(2)
A 4	3.078(4)	10.199(16)	-14.51(2)	0.378(17)	S(2)	-1.274(1)	O(1)	0.725(2)	O(2)	-0.130(1)	O(3)	-0.384(2)
					O(6)	1.077(1)	N(2)	-1.172(2)	C(1)	0.360(2)	C(2)	0.082(2)
					C(3)	0.157(2)	C(4)	-0.081(2)	C(5)	-0.435(2)	C(6)	-0.551(2)
					C(7)	-0.248(2)	C(8)	-0.135(2)	C(12)	0.167(2)	C(13)	0.293(2)
					C(14)	0.198(2)	C(15)	-0.999(2)	C(21)	-1.263(2)	C(22)	-0.515(2)

Nr 17	P	Q	R	S	Sigref	0.002	Sigpln	0.021	Chisq	162.6	Pl.Hyp.	P<5
Plan	-0.6444(7)	0.7299(6)	-0.2279(9)	3.805(18)	#O(6)	0.007(1)	#N(2)	0.006(2)	#C(10)	0.005(2)	#C(20)	-0.018(2)
A 4	-3.232(4)	13.640(12)	-5.80(2)	3.805(18)	O(7)	-0.384(1)	O(8)	-0.385(1)	C(9)	-1.139(2)	C(11)	1.248(2)
					C(12)	1.370(2)	C(13)	0.258(2)	C(14)	-1.001(2)	C(21)	-0.101(2)
					C(22)	1.105(2)	C(23)	-0.314(2)	C(24)	-0.498(2)		

Nr 18	P	Q	R	S	Sigref	0.002	Sigpln	0.012	Chisq	115.0	Pl.Hyp.	P<5
Plan	-0.7141(6)	0.6913(7)	-0.1102(6)	4.929(13)	#O(7)	-0.001(1)	#O(8)	0.009(1)	#N(2)	0.009(1)	#C(21)	-0.009(2)
A 5	-3.581(3)	12.919(12)	-2.804(14)	4.929(13)	#C(23)	-0.008(2)	O(6)	-0.289(1)	C(9)	-1.461(2)	C(10)	-0.260(2)
					C(11)	0.954(2)	C(12)	0.980(2)	C(13)	-0.198(2)	C(14)	-1.422(2)
					C(20)	-0.206(2)	C(22)	1.148(2)	C(24)	0.102(3)		

(Acute) Angles (Degrees) Between Planes (Numbers I,J from List Above)

1, 2=	4.45(9)	1, 3=	5.72(9)	1, 4=	2.14(7)	1, 5=	2.99(7)	1, 6=	1.08(7)	1, 7=	20.32(6)	1, 8=	59.36(10)
1, 9=	86.11(9)	1,10=	61.46(16)	1,11=	85.05(17)	1,12=	5.64(7)	1,13=	9.92(10)	1,14=	4.93(7)	1,15=	6.86(10)
1,16=	10.89(10)	1,17=	87.53(10)	1,18=	85.59(9)	2, 3=	9.19(9)	2, 4=	2.46(8)	2, 5=	6.62(8)	2, 6=	4.34(7)
2, 7=	16.09(7)	2, 8=	62.67(10)	2, 9=	85.65(9)	2,10=	61.03(17)	2,11=	85.65(17)	2,12=	1.42(8)	2,13=	14.30(10)
2,14=	8.04(8)	2,15=	11.11(10)	2,16=	15.17(11)	2,17=	88.28(10)	2,18=	85.19(9)	3, 4=	7.57(7)	3, 5=	2.75(7)
3, 6=	5.13(7)	3, 7=	25.22(6)	3, 8=	59.94(10)	3, 9=	89.07(9)	3,10=	57.55(16)	3,11=	80.16(17)	3,12=	9.83(7)
3,13=	9.01(10)	3,14=	1.28(8)	3,15=	3.29(10)	3,16=	6.77(11)	3,17=	82.54(10)	3,18=	89.64(9)	4, 5=	4.83(5)
4, 6=	2.44(5)	4, 7=	18.17(4)	4, 8=	60.49(8)	4, 9=	85.32(7)	4,10=	61.82(16)	4,11=	85.90(16)	4,12=	3.80(5)
4,13=	11.85(9)	4,14=	6.59(6)	4,15=	9.00(9)	4,16=	13.03(9)	4,17=	88.45(9)	4,18=	84.82(7)	5, 6=	2.40(5)
5, 7=	22.71(4)	5, 8=	59.83(8)	5, 9=	88.74(7)	5,10=	59.25(16)	5,11=	82.39(16)	5,12=	7.44(5)	5,13=	9.23(9)
5,14=	2.00(6)	5,15=	4.64(9)	5,16=	8.66(9)	5,17=	84.83(9)	5,18=	88.19(7)	6, 7=	20.40(3)	6, 8=	60.26(8)
6, 9=	87.14(7)	6,10=	60.39(15)	6,11=	84.03(16)	6,12=	5.33(5)	6,13=	10.44(9)	6,14=	4.18(5)	6,15=	6.77(8)
6,16=	10.83(9)	6,17=	86.53(8)	6,18=	86.61(7)	7, 8=	71.01(8)	7, 9=	78.69(6)	7,10=	66.60(15)	7,11=	86.58(16)
7,12=	15.40(4)	7,13=	29.36(8)	7,14=	24.01(4)	7,15=	27.15(8)	7,16=	31.20(9)	7,17=	83.58(8)	7,18=	78.47(6)
8, 9=	56.73(10)	8,10=	70.55(17)	8,11=	63.18(17)	8,12=	64.08(8)	8,13=	50.93(11)	8,14=	60.75(9)	8,15=	56.73(11)
8,16=	54.76(11)	8,17=	63.28(11)	8,18=	55.74(10)	9,10=	34.72(16)	9,11=	8.97(17)	9,12=	86.40(7)	9,13=	85.02(10)
9,14=	89.31(8)	9,15=	89.43(10)	9,16=	89.23(10)	9,17=	7.26(10)	9,18=	0.99(9)	10,11=	27.3(2)	10,12=	60.08(16)
10,13=	65.18(17)	10,14=	57.45(16)	10,15=	59.80(17)	10,16=	59.72(17)	10,17=	30.41(17)	10,18=	34.94(16)	11,12=	84.95(16)

11,13=	86.02(18)	11,14=	80.42(16)	11,15=	81.62(18)	11,16=	80.27(18)	11,17=	3.20(18)	11,18=	9.66(17)	12,13=	15.55(9)				
12,14=	8.61(6)	12,15=	12.04(9)	12,16=	16.09(9)	12,17=	87.62(9)	12,18=	85.96(7)	13,14=	9.85(9)	13,15=	5.87(11)				
13,16=	5.75(12)	13,17=	88.12(11)	13,18=	84.35(10)	14,15=	4.37(9)	14,16=	8.02(10)	14,17=	82.85(9)	14,18=	89.86(8)				
15,16=	4.06(11)	15,17=	83.88(11)	15,18=	88.82(10)	16,17=	82.37(11)	16,18=	89.90(10)	17,18=	8.15(10)						

(Acute) Angles (Degrees) Between Axes, Lines, and Bonds with L.S.-Planes

Bond	/ Plane NM --> M	1	2	3	4	5	6	7	8	9	10
Axes 0 --->a	/ 0M =	45.77(6),	47.42(7),	40.22(6),	47.11(4),	42.83(4),	44.90(3),	55.96(1),	34.07(7),	45.33(6),	10.63(15),
	1M =	37.23(16),	46.96(4),	43.05(8),	40.85(4),	40.60(8),	37.87(8),	40.12(8),	45.57(6),		
b	/ 0M =	34.27(6),	35.81(7),	36.94(6),	34.47(4),	35.89(4),	35.34(3),	33.67(1),	20.79(7),	43.74(6),	76.11(15),
	1M =	50.08(16),	36.99(4),	28.30(8),	37.38(4),	34.11(8),	33.08(8),	46.88(8),	43.74(6),		
c	/ 0M =	24.31(6),	19.87(7),	28.10(6),	22.22(4),	26.15(4),	24.13(3),	4.44(1),	48.45(7),	7.31(6),	8.84(15),
	1M =	12.34(16),	18.80(4),	33.78(8),	26.82(4),	30.78(8),	34.78(8),	13.17(8),	6.33(6),		
a*	/ 0M =	45.77(6),	47.42(7),	40.22(6),	47.11(4),	42.83(4),	44.90(3),	55.96(1),	34.07(7),	45.33(6),	10.63(15),
	1M =	37.23(16),	46.96(4),	43.05(8),	40.85(4),	40.60(8),	37.87(8),	40.12(8),	45.57(6),		
b*	/ 0M =	34.27(6),	35.81(7),	36.94(6),	34.47(4),	35.89(4),	35.34(3),	33.67(1),	20.79(7),	43.74(6),	76.11(15),
	1M =	50.08(16),	36.99(4),	28.30(8),	37.38(4),	34.11(8),	33.08(8),	46.88(8),	43.74(6),		
c*	/ 0M =	24.31(6),	19.87(7),	28.10(6),	22.22(4),	26.15(4),	24.13(3),	4.44(1),	48.45(7),	7.31(6),	8.84(15),
	1M =	12.34(16),	18.80(4),	33.78(8),	26.82(4),	30.78(8),	34.78(8),	13.17(8),	6.33(6),		
S(1) -C(17)	/ 0M =	19.73(9),	15.28(9),	23.13(9),	17.68(7),	21.35(7),	19.46(7),	0.32(6),	51.21(10),	3.03(9),	2.41(16),
	1M =	7.41(17),	14.14(7),	29.44(10),	21.86(7),	25.97(10),	29.88(10),	8.49(10),	2.06(9),		
S(2) -C(22)	/ 0M =	30.48(9),	31.13(9),	25.45(9),	31.38(7),	27.77(7),	29.48(7),	37.41(6),	41.62(10),	63.19(9),	28.91(16),
	1M =	54.51(17),	30.40(7),	30.51(10),	25.79(8),	26.64(10),	24.83(11),	57.05(10),	63.60(9),		
O(1) -C(1)	/ 0M =	7.45(11),	3.01(12),	10.70(11),	5.43(10),	8.97(10),	7.13(10),	11.62(9),	46.79(12),	1.71(11),	5.70(18),
	1M =	4.33(18),	1.83(10),	17.25(12),	9.43(10),	13.58(12),	17.45(13),	6.02(12),	0.81(11),		
O(2) -C(8)	/ 0M =	10.39(11),	14.84(11),	7.00(11),	12.42(10),	8.80(10),	10.69(10),	29.45(9),	33.74(12),	3.95(11),	13.31(18),
	1M =	3.81(18),	16.00(10),	0.62(12),	8.27(10),	4.18(12),	0.26(13),	6.26(12),	3.22(11),		
O(3) -C(15)	/ 0M =	40.06(11),	43.20(11),	34.38(11),	41.94(10),	37.13(10),	39.50(10),	57.07(9),	16.90(12),	38.93(11),	5.61(18),
	1M =	32.74(18),	43.32(10),	34.16(12),	35.36(10),	33.62(12),	30.05(13),	35.93(12),	38.80(11),		
O(4) -C(18)	/ 0M =	58.36(16),	62.38(16),	56.46(16),	59.98(15),	57.67(15),	59.00(15),	69.65(14),	0.43(16),	5.79(16),	39.9(2),
	1M =	12.6(2),	63.77(15),	48.58(16),	57.66(15),	53.32(16),	49.75(17),	9.47(16),	5.76(16),		
O(5) -C(18)	/ 0M =	21.80(11),	25.45(11),	16.31(11),	23.83(9),	19.00(9),	21.40(9),	40.91(9),	0.76(11),	38.80(11),	11.55(17),
	1M =	35.92(18),	25.85(9),	14.90(12),	17.42(10),	15.07(12),	11.27(12),	38.93(12),	38.26(11),		
-C(19)	/ 0M =	34.23(13),	32.44(14),	39.70(13),	32.89(13),	37.15(12),	35.11(12),	20.35(12),	2.15(14),	48.91(13),	64.53(19),
	1M =	57.7(2),	32.74(12),	35.30(14),	39.14(13),	38.94(14),	40.97(15),	56.08(14),	48.11(13),		
O(6) -C(20)	/ 0M =	70.71(11),	75.13(11),	67.13(11),	72.68(10),	69.12(10),	71.04(10),	82.36(9),	14.68(12),	3.67(11),	31.04(18),
	1M =	4.18(18),	76.34(10),	60.84(12),	68.41(10),	64.49(12),	60.50(13),	1.17(12),	3.89(11),		
O(7) -C(23)	/ 0M =	59.08(11),	63.42(12),	56.15(11),	60.96(10),	57.82(10),	59.53(10),	74.05(10),	3.32(12),	0.33(11),	33.72(18),
	1M =	6.50(18),	64.71(10),	49.16(12),	57.42(10),	53.25(12),	49.39(13),	3.33(12),	0.35(11),		
O(8) -C(23)	/ 0M =	4.27(11),	8.70(11),	1.19(11),	6.26(10),	2.85(9),	4.63(9),	22.90(9),	40.64(11),	0.06(11),	13.57(17),
	1M =	0.89(18),	9.90(9),	5.59(12),	2.45(10),	1.74(12),	5.58(12),	3.09(12),	0.74(11),		
-C(24)	/ 0M =	60.05(13),	55.61(14),	62.85(13),	58.04(12),	61.42(12),	59.70(12),	40.63(12),	51.30(14),	2.80(13),	18.93(19),
	1M =	5.6(2),	54.42(12),	69.84(14),	61.64(13),	65.94(14),	69.56(15),	4.46(14),	3.68(13),		
N(1) -C(15)	/ 0M =	56.37(10),	53.26(11),	55.04(11),	55.37(9),	55.57(9),	55.41(9),	44.26(9),	64.02(11),	25.21(11),	0.01(17),

```
=====================================================================================================================
              1M =  16.63(18),  51.85(9), 63.91(12), 54.40(10), 58.05(12), 59.13(12), 17.95(12), 26.06(11),
    -C(16)  / OM =  11.50(10),  7.04(10), 15.08(10),   9.43(9),  13.21(9),  11.25(8),   8.07(8), 45.56(11),  5.71(10),  0.06(17),
              1M =   8.93(18),   5.93(9), 21.13(11),  13.80(9), 17.84(11), 21.79(12), 10.44(11),  4.78(10),
N(2)  -C(20)  / OM =  52.77(10), 48.44(11), 54.78(11),  50.90(9),  53.70(9),  52.25(9),  34.51(9), 59.78(11),  8.22(10), 10.75(17),
              1M =   0.26(18),  47.15(9), 62.68(12), 53.63(10), 58.00(12), 61.29(12),  1.03(11),  9.15(11),
    -C(21)  / OM =   6.36(10), 10.80(10),  3.17(10),   8.37(9),   4.88(9),   6.70(8),  25.16(8), 38.24(11),  1.45(10), 13.46(17),
              1M =   1.95(18),  11.99(9),  3.47(11),   4.44(9),  0.28(11),  3.59(12),  4.24(11),  0.67(10),
C(1)  -C(2)   / OM =   2.01(13),  0.51(13),  7.71(13),  0.37(12),  5.00(12),  2.74(12), 13.64(12),  6.30(14), 61.15(13), 42.66(19),
              1M =  63.13(19),  0.42(12),  5.61(14),  6.91(12),  7.76(14), 10.72(14), 65.27(14), 60.22(13),
    -C(14)  / OM =   4.81(13),  2.76(13),  2.47(13),  4.38(12),  3.40(12),  3.76(12),  0.77(12), 58.11(14), 56.73(13), 51.28(19),
              1M =    53.9(2),  1.47(12), 11.13(14),  1.98(12),  5.32(14),  6.27(14), 52.45(14), 57.59(13),
C(2)  -C(3)   / OM =   1.55(14),  0.48(14),  0.79(14),  1.13(13),  0.14(13),  0.50(13),  2.24(12), 55.29(14), 56.74(14),    54.2(2),
              1M =    54.7(2),  1.76(13),  7.89(15),  1.28(13),  2.07(15),  3.05(15), 53.05(15), 57.54(14),
    -C(7)   / OM =   3.59(14),  0.86(14),  6.87(14),  1.56(13),  5.13(13),  3.27(13), 15.49(12), 44.13(14),  2.27(14),     7.4(2),
              1M =     4.3(2),  2.03(13), 13.38(15),  5.60(13),  9.74(15), 13.62(15),  6.17(14),  1.39(14),
C(3)  -C(4)   / OM =   1.33(14),  1.09(14),  7.02(14),  0.28(13),  4.31(13),  2.08(13), 13.94(12),  8.00(14), 62.83(14),    43.5(2),
              1M =    64.6(2),  0.96(13),  4.68(15),  6.24(13),  6.97(15),  9.85(15), 66.82(15), 61.91(14),
C(4)  -C(5)   / OM =   4.78(14),  0.33(14),  8.08(14),  2.75(13),  6.33(13),  4.47(13), 14.33(12), 44.71(15),  2.43(14),     6.6(2),
              1M =     4.6(2),  0.83(13), 14.56(15),  6.81(13), 10.94(15), 14.83(15),  6.46(15),  1.55(14),
C(5)  -C(6)   / OM =   3.11(14),  1.01(14),  0.85(14),  2.65(13),  1.74(13),  2.06(13),  1.10(12), 57.10(14), 55.91(14),    52.4(2),
              1M =    53.5(2),  0.29(13),  9.55(15),  0.34(13),  3.73(15),  4.75(15), 51.94(15), 56.74(14),
C(6)  -C(7)   / OM =   3.99(14),  1.59(14),  9.68(14),  2.39(13),  6.97(13),  4.75(13), 11.29(12),  6.85(14), 62.47(14),    45.3(2),
              1M =    65.2(2),  1.71(13),  7.26(15),  8.90(13),  9.61(14), 12.46(15), 67.10(14), 61.51(14),
    -C(15)  / OM =   6.91(13),  2.46(13), 10.69(13),  4.81(12),  8.72(12),  6.71(12), 12.86(11), 41.00(14),  8.52(13),  0.05(19),
              1M =  11.06(19),  1.38(12), 16.44(14),  9.41(12), 13.36(14), 17.36(14), 12.79(14),  7.61(13),
C(7)  -C(8)   / OM =   5.93(10),  7.95(10),  8.24(10),   6.35(9),   7.34(9),   6.98(8),   9.28(8), 48.73(11), 55.39(10), 60.32(17),
              1M =  55.42(17),   9.22(8),  0.47(11),   8.74(9),  5.36(11),  4.30(11), 53.20(11), 56.04(10),
C(8)  -C(9)   / OM =   3.20(13),  5.70(13),  2.51(13),  4.84(12),  0.21(12),  2.46(12), 18.72(12),  8.93(14), 61.75(13), 39.30(19),
              1M =    62.0(2),  5.61(12),  0.48(14),  1.70(12),  2.57(14),  5.55(14), 64.59(14), 60.91(13),
C(9)  -C(10)  / OM =   0.21(10),  1.70(11),  2.27(10),   0.15(9),   1.28(9),   0.85(9),   2.95(8), 53.29(11), 58.22(10), 56.15(17),
              1M =  56.56(18),   2.97(9),  6.34(11),   2.73(9),  0.55(11),  1.43(12), 54.80(11), 59.00(10),
    -C(14)  / OM =   2.31(10),  6.75(11),  0.98(10),   4.33(9),   0.77(9),   2.62(9),  21.30(8), 40.33(11),  2.41(10), 10.45(17),
              1M =   3.54(18),   7.92(9),  7.49(11),   0.29(9),  3.84(11),  7.73(12),  5.67(11),  1.59(10),
C(10) -C(11)  / OM =   7.41(14),  9.94(14),  1.70(14),  9.06(13),  4.42(13),  6.68(13), 22.92(12), 10.66(14), 61.19(14),    36.1(2),
              1M =    60.1(2),  9.85(13),  3.61(15),  2.51(13),  1.61(15),  1.41(15), 62.96(15), 60.43(14),
    -C(20)  / OM =  11.00(13), 15.41(13),  8.06(13), 12.96(12),  9.66(12), 11.40(12), 29.26(11), 36.69(14),  1.35(13), 18.36(19),
              1M =   1.57(19), 16.64(12),  1.11(14),  9.31(12),  5.10(14),  1.29(14),  0.88(14),  2.07(13),
C(11) -C(12)  / OM =   3.43(14),  7.87(14),  0.20(14),  5.44(13),  1.93(13),  3.76(13), 22.34(12), 39.94(14),  1.89(14),    11.5(2),
              1M =     2.9(2),  9.06(13),  6.38(15),  1.46(13),  2.68(15),  6.56(15),  5.02(15),  1.09(14),
C(12) -C(13)  / OM =   3.96(14),  1.98(14),  1.52(14),  3.56(13),  2.50(13),  2.90(13),  0.33(12), 56.82(15), 57.78(14),    52.5(2),
              1M =    55.1(2),  0.71(13), 10.15(15),  1.06(13),  4.35(15),  5.24(15), 53.64(15), 58.63(14),
C(13) -C(14)  / OM =   4.79(14),  7.28(14),  0.91(14),  6.43(13),  1.81(13),  4.06(13), 20.21(12),  9.96(14), 62.02(14),    38.4(2),
              1M =    61.7(2),  7.18(13),  1.14(15),  0.11(13),  0.96(15),  3.93(15), 64.44(15), 61.21(14),
C(16) -C(17)  / OM =  27.66(13), 26.35(13), 24.27(13), 27.63(12), 25.74(12), 26.58(12), 25.95(11), 62.74(14), 58.24(13), 33.12(19),
              1M =  50.23(19), 25.18(12), 32.06(14), 24.06(12), 26.65(14), 26.55(14), 51.08(14), 59.18(13),
    -C(18)  / OM =  35.93(13), 34.62(13), 41.15(13), 34.80(12), 38.74(12), 36.88(12), 23.68(11),  1.22(14), 49.22(13), 69.78(19),
              1M =  58.18(19), 35.08(12), 35.77(14), 40.74(12), 39.97(14), 41.52(14), 56.10(14), 48.53(13),
C(21) -C(22)  / OM =  29.74(10), 31.03(10), 32.76(10),  29.81(8),  31.53(8),  30.82(8),  28.27(8), 23.56(11), 49.22(10), 80.16(17),
```

```
             1M =   55.39(17),  32.16(8), 24.34(11),  33.11(9), 30.07(11), 29.35(11), 52.19(11), 49.22(10),
    -C(23)  / OM =   64.64(13), 60.20(13), 68.15(13), 62.54(12), 66.40(12), 64.45(12), 44.60(11), 45.40(14),  0.81(13), 24.44(19),
             1M =    9.46(19), 59.12(12), 73.89(14), 66.89(12), 71.03(14), 74.92(14),  8.06(14),  0.02(13),
```

Ring Puckering Analysis (Cremer & Pople) - (e.s.d. following Norrestam, Acta Cryst. (1981), A37, 764-765)

	Symmetrical Forms	References
6-Membered Rings :	C : Chair - Th = 0.0	J.C.A. Boeyens, J.Cryst.Mol.Struct. 8,(1978),317-320
	H : Half-Chair- Th = 50.8; Phi = k X 60 + 30	
	E : Envelope - Th = 54.7; Phi = k X 60	
	S : Screw-Boat- Th = 67.5; Phi = k X 60 + 30	
	B : Boat - Th = 90.0; Phi = k X 60	
	T : Twist-Boat- Th = 90.0; Phi = k X 60 + 30	

Definitions (All Values Rounded on Esd)

Dev - Deviation of Atom I from Cremer&Pople Plane (Defined Differently from Least-Squares Plane)
Cs(I),C2(I) - Mirror Plane and 2-Axis Asym. Par. for Atom I (See Duax et al., Topics in Stereochemistry,V-9, (1976) pp.271-383)
Cs(I-J),C2(I-J)- Asymmetry Parameters for Bond I-J
Tors(I-J) - Torsion Angle for Bond I-J

Descriptors for Torsion Angles

Torsion Angle Range	Full Descriptor	Short Descriptor
0 TO 30 Deg	+ Syn-Periplanar	+sp
30 to 90	+ Syn-Clinal	+sc
90 to 150	+ Anti-Clinal	+ac
150 to 180	+ Anti-Periplanar	+ap
0 to -30	- Syn-Periplanar	-sp
-30 to -90	- Syn-Clinal	-sc
-90 to -150	- Anti-Clinal	-ac
-150 to -180	- Anti-Periplanar	-ap

Descriptors for Ring Substituents (J.Appl.Cryst.,1983,16,431)

Angle Range of Subst.	Full Descriptor	Short Descriptor
0 TO 30 Deg.	Axial	ax
30 to 60	Bisectional	bi
60 to 90	Equatorial	eq

*** NOTE *** - For Ring Puckering Comparisons: Make Sure that the Absolute Configuration, Pivot Atom and Cyclic Sense Agree.
- The "RING AT1 AT2 AT3 ... ATn" Instruction Gives the User Explicit Choice of Pivot Atom (AT1) and Sense (AT2).
- Use TRNS Instructions to Obtain the Required Absolute Configuration.
- The Values of Theta and Phi [= Phi(2)] Depend on the Abs. Conf. and the Choice of the First and Second Ring Atom.
- Alternatively, Appropriate Phase Shifts may be Applied to the Same Effect (see Below)

For Correct Usage of C&P Puckering Parameters see also: D. Cremer, Acta Cryst. (1984). B40, 498-500.

6-Membered Ring (1) C(1) --> C(2) --> C(7) --> C(8) --> C(9) --> C(14) -->

	C(1)	C(2)	C(7)	C(8)	C(9)	C(14)
	sp2	sp2	sp2	sp2	sp2	sp2
Dev. (Ang)	0.062(2)	0.009(2)	-0.078(2)	0.0769(19)	-0.0063(19)	-0.063(2)
Cs(I)-Asym-Par (Deg)	9.5(2)	17.5(2)	8.0(2)	9.5(2)	17.5(2)	8.0(2)
C2(I)-Asym-Par (Deg)	15.3(2)	4.2(2)	16.1(2)	15.3(2)	4.2(2)	16.1(2)
Ring Bond Angle(Deg)	118.07(15)	120.84(16)	120.04(16)	117.89(14)	120.62(16)	120.72(17)

Tors(I-J) (Deg)	-3.6(3)	-8.8(3)	14.4(3)	-7.6(3)	-4.6(3)	10.4(3)
Cs(I-J)-Asym-Par (Deg)	7.0(3)	7.9(3)	13.0(3)	7.0(3)	7.9(3)	13.0(3)
C2(I-J)-Asym-Par (Deg)	19.1(3)	18.0(3)	1.1(3)	19.1(3)	18.0(3)	1.1(3)
Ring Bond Distance (Ang)	1.494(3)	1.401(3)	1.500(2)	1.492(3)	1.403(2)	1.488(3)

Weighted Average Ring Bond Distance = 1.4596(10,202) Ang. - NOTE: 1st esd. Internal, 2nd esd External.
Weighted Average Abs. Torsion Angl. = 8.23(12,161) Deg. see: e.g. Domenicano et al., Acta Cryst.(1975), B31, 221-234.

Cremer & Pople Puckering Parameters [D. Cremer & J.A. Pople, J.Amer.Chem.Soc., 97, (1975), 1354-1358]

Q(2) = 0.140(2) Ang., Phi(2) = 329.1(8) Deg
Q(3) = -0.019(2) Ang.

Puckering Amplitude (Q) = 0.141(2) Ang, Theta = 97.5(8) Deg, Phi = 329.1(8) Deg

* NOTE * - A Change of the Absolute Configuration Transforms Theta into 180 - Theta and Phi into 180 + Phi.
- A Cyclic Forward Shift of the Pivot Atom from At1 to At2 Transforms Theta into 180 - Theta and Phi into Phi + 120.
- A Change of the Sense Transforms Theta into 180 - Theta and Phi into 180 - Phi, and Vice Versa.

Conformational Analysis (G.G. Evans & J.A. Boeyens, Acta Cryst. (1989), B45, 581-590)

Coefficients of Primitive and Normalised Forms

	M	Primitive	Coefficient	Angular Value
CosForm	2	0.004	0.028	20.0
SinForm		0.136	0.856	22.0
	3		0.116	-1.0

Centroid Cg(1)	x ,y ,z	0.69218(16)	0.45847(4)	0.44001(3)
	XO,YO,ZO	3.4711(8)	8.5675(7)	11.1923(7)

6-Membered Ring (2) C(2) --> C(3) --> C(4) --> C(5) --> C(6) --> C(7) -->

	sp2	sp2	sp2	sp2	sp2	sp2
Dev. (Ang)	-0.002(2)	-0.014(2)	0.013(2)	0.004(2)	-0.020(2)	0.019(2)
Cs(I)-Asym-Par (Deg)	4.3(2)	2.0(2)	2.4(2)	4.3(2)	2.0(2)	2.4(2)
C2(I)-Asym-Par (Deg)	1.8(2)	4.3(2)	4.0(2)	1.8(2)	4.3(2)	4.0(2)
Ring Bond Angle(Deg)	120.73(17)	120.04(18)	119.45(19)	121.52(18)	119.07(17)	119.07(16)

Tors(I-J) (Deg)	-0.8(3)	2.2(3)	-0.5(3)	-2.6(3)	3.9(3)	-2.3(3)
Cs(I-J)-Asym-Par (Deg)	2.4(3)	3.6(3)	2.2(3)	2.4(3)	3.6(3)	2.2(3)
C2(I-J)-Asym-Par (Deg)	4.5(3)	0.3(3)	4.8(3)	4.5(3)	0.3(3)	4.8(3)
Ring Bond Distance (Ang)	1.393(3)	1.386(3)	1.384(3)	1.396(3)	1.404(3)	1.401(3)

Weighted Average Ring Bond Distance = 1.3940(12, 32) Ang. - NOTE: 1st esd. Internal, 2nd esd External.
Weighted Average Abs. Torsion Angl. = 2.05(12, 51) Deg. see: e.g. Domenicano et al., Acta Cryst.(1975), B31, 221-234.

No C & P - Puckering Analysis since <Tau> = 2.0 < 5.0 Deg.

Centroid Cg(2) : x ,y ,z	1.00902(17)	0.35716(4)	0.45143(3)
XO,YO,ZO	5.0599(9)	6.6744(7)	11.4829(8)

6-Membered Ring (3) C(9) --> C(10) --> C(11) --> C(12) --> C(13) --> C(14) -->

	sp2	sp2	sp2	sp2	sp2	sp2
Dev. (Ang)	-0.0249(19)	0.031(2)	-0.011(2)	-0.015(2)	0.021(2)	-0.001(2)
Cs(I)-Asym-Par (Deg)	4.2(2)	1.9(2)	6.1(2)	4.2(2)	1.9(2)	6.1(2)
C2(I)-Asym-Par (Deg)	5.3(2)	6.5(2)	3.0(2)	5.3(2)	6.5(2)	3.0(2)
Ring Bond Angle(Deg)	119.30(17)	119.42(16)	120.59(17)	120.21(18)	119.85(17)	120.39(17)

Tors(I-J) (Deg)	5.6(3)	-4.3(3)	0.0(3)	3.0(3)	-1.7(3)	-2.7(3)
Cs(I-J)-Asym-Par (Deg)	5.1(3)	4.0(3)	2.9(3)	5.1(3)	4.0(3)	2.9(3)
C2(I-J)-Asym-Par (Deg)	1.7(3)	5.7(3)	7.3(3)	1.7(3)	5.7(3)	7.3(3)
Ring Bond Distance (Ang)	1.401(2)	1.395(3)	1.390(3)	1.380(3)	1.396(3)	1.403(2)

Weighted Average Ring Bond Distance = 1.3965(10, 33) Ang. - NOTE: 1st esd. Internal, 2nd esd External.
Weighted Average Abs. Torsion Angl. = 2.88(12, 80) Deg. see: e.g. Domenicano et al., Acta Cryst.(1975), B31, 221-234.

No C & P - Puckering Analysis since <Tau> = 2.9 < 5.0 Deg.

Centroid Cg(3) : x ,y ,z	0.35035(16)	0.55380(4)	0.43008(3)
X0,Y0,Z0	1.7569(8)	10.3489(7)	10.9398(7)

10-Membered Ring (4) C(1) --> C(2) --> C(3) --> C(4) --> C(5) --> C(6) --> C(7) --> C(8) -->

	C(1)	C(2)	C(3)	C(4)	C(5)	C(6)	C(7)	C(8)
	sp2	sp2	sp2	sp2	sp2	sp2	sp2	sp2
Dev. (Ang)	0.008(2)	0.0041(19)	0.031(2)	0.043(2)	-0.018(2)	-0.0811(19)	-0.0279(19)	0.1391(18)
Cs(I)-Asym-Par (Deg)	158.04(16)	220.70(11)	159.10(16)	107.29(16)	106.41(15)	158.04(16)	220.70(11)	159.10(16)
C2(I)-Asym-Par (Deg)	154.14(16)	4.97(18)	153.04(16)	192.93(13)	193.42(13)	154.14(16)	4.97(18)	153.04(16)
Ring Bond Angle(Deg)	118.07(15)	118.43(16)	120.04(18)	119.45(19)	121.52(18)	119.07(17)	120.58(16)	117.89(14)
Tors(I-J) (Deg)	176.33(17)	179.30(19)	2.2(3)	-0.5(3)	-2.6(3)	-169.67(17)	-172.07(17)	-7.6(3)
Cs(I-J)-Asym-Par (Deg)	129.00(18)	125.94(17)	172.29(17)	5.45(13)	170.87(17)	129.00(18)	125.94(17)	172.29(17)
C2(I-J)-Asym-Par (Deg)	119.73(18)	119.07(17)	176.65(17)	246.73(12)	177.94(17)	119.73(18)	119.07(17)	176.65(17)
Ring Bond Distance (Ang)	1.494(3)	1.393(3)	1.386(3)	1.384(3)	1.396(3)	1.404(3)	1.500(2)	1.492(3)

(Continued) C(9) --> C(14) -->

	C(9)	C(14)
	sp2	sp2
Dev. (Ang)	0.0074(18)	-0.105(2)
Cs(I)-Asym-Par (Deg)	107.29(16)	106.41(15)
C2(I)-Asym-Par (Deg)	192.93(13)	193.42(13)
Ring Bond Angle(Deg)	120.62(16)	120.72(17)
Tors(I-J) (Deg)	-4.6(3)	10.4(3)
Cs(I-J)-Asym-Par (Deg)	5.45(13)	170.87(17)
C2(I-J)-Asym-Par (Deg)	246.73(12)	177.94(17)
Ring Bond Distance (Ang)	1.403(2)	1.488(3)

Weighted Average Ring Bond Distance = 1.4375(8,165) Ang. - NOTE: 1st esd. Internal, 2nd esd External.
Weighted Average Abs. Torsion Angl. = 117.08(7,999) Deg. see: e.g. Domenicano et al., Acta Cryst.(1975), B31, 221-234.

Cremer & Pople Puckering Parameters [D. Cremer & J.A. Pople, J.Amer.Chem.Soc., 97, (1975), 1354-1358]

Q(2) = 0.152(2) Ang., Phi(2) = 209.0(7) Deg
Q(3) = 0.121(2) Ang., Phi(3) = 325.0(9) Deg
Q(4) = 0.057(2) Ang., Phi(4) = 26(2) Deg
Q(5) = 0.000(2) Ang.

Total Puckering Amplitude Q = 0.2021(19) Ang.

Conformational Analysis (G.G. Evans & J.A. Boeyens, Acta Cryst. (1989), B45, 581-590)

Coefficients of Primitive and Normalised Forms

	M	Primitive	Coefficient	Angular Value
CosForm	2	0.094	0.282	24.0
SinForm		0.060	0.180	22.0
CosForm	3	0.115	0.345	36.0
SinForm		0.007	0.021	38.0
CosForm	4	0.026	0.077	4.0
SinForm		0.032	0.096	2.0
	5		0.000	1.0

10-Membered Ring (5) C(1) --> C(2) --> C(7) --> C(8) --> C(9) --> C(10) --> C(11) --> C(12) -->

	sp2	sp2	sp2	sp2	sp2	sp2	sp2	sp2
Dev. (Ang)	0.114(2)	-0.0202(19)	-0.1493(19)	0.0418(18)	0.0403(18)	0.0713(19)	-0.033(2)	-0.076(2)
Cs(I)-Asym-Par (Deg)	160.44(15)	104.68(15)	191.38(13)	161.09(15)	157.21(11)	160.44(15)	104.68(15)	191.38(13)
C2(I)-Asym-Par (Deg)	152.80(15)	195.27(13)	111.64(16)	152.11(15)	156.12(11)	152.80(15)	195.27(13)	111.64(16)
Ring Bond Angle(Deg)	118.07(15)	120.84(16)	120.04(16)	117.89(14)	120.08(15)	119.42(16)	120.59(17)	120.21(18)
Tors(I-J) (Deg)	-3.6(3)	-8.8(3)	14.4(3)	173.18(17)	-175.18(17)	-4.3(3)	0.0(3)	3.0(3)
Cs(I-J)-Asym-Par (Deg)	174.36(17)	177.15(12)	174.79(17)	116.94(17)	124.59(17)	174.36(17)	177.15(12)	174.79(17)
C2(I-J)-Asym-Par (Deg)	175.91(17)	173.03(12)	175.22(17)	129.14(17)	125.10(17)	175.91(17)	173.03(12)	175.22(17)
Ring Bond Distance (Ang)	1.494(3)	1.401(3)	1.500(2)	1.492(3)	1.401(2)	1.395(3)	1.390(3)	1.380(3)

(Continued) C(13) --> C(14) -->

	sp2	sp2
Dev. (Ang)	-0.015(2)	0.025(2)
Cs(I)-Asym-Par (Deg)	161.09(15)	157.21(11)
C2(I)-Asym-Par (Deg)	152.11(15)	156.12(11)
Ring Bond Angle(Deg)	119.85(17)	118.84(16)
Tors(I-J) (Deg)	-178.96(18)	-172.31(17)
Cs(I-J)-Asym-Par (Deg)	116.94(17)	124.59(17)
C2(I-J)-Asym-Par (Deg)	129.14(17)	125.10(17)
Ring Bond Distance (Ang)	1.396(3)	1.488(3)

Weighted Average Ring Bond Distance = 1.4371(8,166) Ang. - NOTE: 1st esd. Internal, 2nd esd External.
Weighted Average Abs. Torsion Angl. = 118.80(7,999) Deg. see: e.g. Domenicano et al., Acta Cryst.(1975), B31, 221-234.

Cremer & Pople Puckering Parameters [D. Cremer & J.A. Pople, J.Amer.Chem.Soc., 97, (1975), 1354-1358]

Q(2) = 0.1951(19) Ang., Phi(2) = 36.7(6) Deg
Q(3) = 0.0685(19) Ang., Phi(3) = 346.2(16) Deg
Q(4) = 0.0916(19) Ang., Phi(4) = 303.9(12) Deg
Q(5) = -0.0269(19) Ang.

Total Puckering Amplitude Q = 0.2274(19) Ang.

Conformational Analysis (G.G. Evans & J.A. Boeyens, Acta Cryst. (1989), B45, 581-590)

Coefficients of Primitive and Normalised Forms

	M	Primitive	Coefficient	Angular Value
CosForm	2	0.188	0.490	4.0
SinForm		0.008	0.020	6.0
CosForm	3	0.016	0.042	40.0
SinForm		0.053	0.138	38.0
CosForm	4	0.011	0.028	32.0
SinForm		0.081	0.212	34.0
	5		0.070	-1.0

14-Membered Ring (6)	C(1) -->	C(2) -->	C(3) -->	C(4) -->	C(5) -->	C(6) -->	C(7) -->	C(8) -->
	sp2	sp2	sp2	sp2	sp2	sp2	sp2	sp2
Dev. (Ang)	0.116(2)	0.0457(19)	0.059(2)	0.010(2)	-0.098(2)	-0.1489(19)	-0.0340(19)	0.1474(18)
Cs(I)-Asym-Par (Deg)	130.39(9)	188.04(11)	209.35(12)	160.16(11)	208.60(10)	160.57(13)	228.18(11)	130.39(9)
C2(I)-Asym-Par (Deg)	229.64(9)	185.41(11)	160.96(13)	209.96(10)	161.93(11)	209.65(12)	132.93(13)	229.64(9)
Ring Bond Angle(Deg)	118.07(15)	118.43(16)	120.04(18)	119.45(19)	121.52(18)	119.07(17)	120.58(16)	117.89(14)
Tors(I-J) (Deg)	176.33(17)	179.30(19)	2.2(3)	-0.5(3)	-2.6(3)	-169.67(17)	-172.07(17)	173.18(17)
Cs(I-J)-Asym-Par (Deg)	174.24(11)	173.50(14)	246.32(12)	144.64(10)	202.19(12)	173.51(14)	103.99(14)	174.24(11)
C2(I-J)-Asym-Par (Deg)	175.04(12)	174.10(14)	143.79(14)	245.84(10)	201.18(12)	176.01(14)	225.34(11)	175.04(12)
Ring Bond Distance (Ang)	1.494(3)	1.393(3)	1.386(3)	1.384(3)	1.396(3)	1.404(3)	1.500(2)	1.492(3)

(Continued)	C(9) -->	C(10) -->	C(11) -->	C(12) -->	C(13) -->	C(14) -->
	sp2	sp2	sp2	sp2	sp2	sp2
Dev. (Ang)	0.0820(18)	0.1018(19)	-0.062(2)	-0.153(2)	-0.083(2)	0.017(2)
Cs(I)-Asym-Par (Deg)	188.04(11)	209.35(12)	160.16(11)	208.60(10)	160.57(13)	228.18(11)
C2(I)-Asym-Par (Deg)	185.41(11)	160.96(13)	209.96(10)	161.93(11)	209.65(12)	132.93(13)
Ring Bond Angle(Deg)	120.08(15)	119.42(16)	120.59(17)	120.21(18)	119.85(17)	118.84(16)
Tors(I-J) (Deg)	-175.18(17)	-4.3(3)	0.0(3)	3.0(3)	-178.96(18)	-172.31(17)
Cs(I-J)-Asym-Par (Deg)	173.50(14)	246.32(12)	144.64(10)	202.19(12)	173.51(14)	103.99(14)
C2(I-J)-Asym-Par (Deg)	174.10(14)	143.79(14)	245.84(10)	201.18(12)	176.01(14)	225.34(11)
Ring Bond Distance (Ang)	1.401(2)	1.395(3)	1.390(3)	1.380(3)	1.396(3)	1.488(3)

Weighted Average Ring Bond Distance = 1.4258(7,133) Ang. - NOTE: 1st esd. Internal, 2nd esd External.
Weighted Average Abs. Torsion Angl. = 139.92(5,999) Deg. see: e.g. Domenicano et al., Acta Cryst.(1975), B31, 221-234.

Cremer & Pople Puckering Parameters [D. Cremer & J.A. Pople, J.Amer.Chem.Soc., 97, (1975), 1354-1358]

Q(2)	=	0.322(2) Ang.,	Phi(2) =	316.9(3) Deg
Q(3)	=	0.090(2) Ang.,	Phi(3) =	104.4(12) Deg
Q(4)	=	0.082(2) Ang.,	Phi(4) =	58.4(14) Deg
Q(5)	=	0.0369(19) Ang.,	Phi(5) =	252(3) Deg
Q(6)	=	0.0731(19) Ang.,	Phi(6) =	15.1(16) Deg
Q(7)	=	-0.011(2) Ang.		

Total Puckering Amplitude Q = 0.3539(19) Ang.

Conformational Analysis (G.G. Evans & J.A. Boeyens, Acta Cryst. (1989), B45, 581-590)

Coefficients of Primitive and Normalised Forms

	M	Primitive	Coefficient	Angular Value
CosForm	2	0.114	0.185	48.0
SinForm		0.209	0.339	50.0
CosForm	3	0.080	0.129	16.0
SinForm		0.011	0.018	18.0
CosForm	4	0.038	0.061	8.0
SinForm		0.045	0.072	10.0
CosForm	5	0.022	0.036	40.0
SinForm		0.015	0.024	38.0
CosForm	6	0.013	0.021	4.0
SinForm		0.061	0.098	2.0
	7		0.017	-1.0

Analysis of Short Ring-Interactions with Cg-Cg Distances < 6.0 Angstrom and Beta < 60.0Deg.

- Cg(I) = Plane number I (= ring number in () above)
- Alpha = Dihedral Angle between Planes I and J (Deg)
- Beta = Angle Cg(I)-->Cg(J) or Cg(I)-->Me vector and normal to plane I (Deg)
- Gamma = Angle Cg(I)-->Cg(J) vector and normal to plane J (Deg)
- Cg-Cg = Distance between ring Centroids (Ang.)
- CgI_Perp = Perpendicular distance of Cg(I) on ring J (Ang.)
- CgJ_Perp = Perpendicular distance of Cg(J) on ring I (Ang.)
- Slippage = Distance between Cg(I) and Perpendicular Projection of Cg(J) on Ring I (Ang).
- P,Q,R,S = J-Plane Parameters for Carth. Coord. (Xo, Yo, Zo)

Cg(I)	Res(I)		Cg(J)	[ARU(J)]	Cg-Cg	Transformed J-Plane P,	Q,	R,	S	Alpha	Beta	Gamma	CgI_Perp	CgJ_Perp	Slippage
Cg(1)	[1]	->	Cg(1)	[1455.01]	5.0147(12)	0.7165	0.5631	-0.4117	-0.8900	0.00(9)	44.2	44.2	3.5932(8)	-3.5931(8)	3.498
Cg(1)	[1]	->	Cg(1)	[1655.01]	5.0147(12)	0.7165	0.5631	-0.4117	6.2963	0.00(9)	44.2	44.2	-3.5931(8)	3.5932(8)	3.498
Cg(1)	[1]	->	Cg(2)	[1455.01]	3.9249(12)	0.7363	0.5851	-0.3399	0.0355	4.45(9)	22.0	18.2	3.7289(8)	-3.6403(8)	
Cg(1)	[1]	->	Cg(3)	[1655.01]	3.7591(11)	0.6457	0.6009	-0.4711	5.4384	5.72(9)	22.5	27.9	-3.3208(8)	3.4721(8)	
Cg(2)	[1]	->	Cg(1)	[1655.01]	3.9250(12)	0.7165	0.5631	-0.4117	6.2963	4.45(9)	18.2	22.0	-3.6404(8)	3.7290(8)	
Cg(2)	[1]	->	Cg(2)	[1455.01]	5.0147(12)	0.7363	0.5851	-0.3399	0.0355	0.00(9)	42.6	42.6	3.6925(8)	-3.6925(8)	3.393
Cg(2)	[1]	->	Cg(2)	[1655.01]	5.0148(12)	0.7363	0.5851	-0.3399	7.4205	0.00(9)	42.6	42.6	-3.6925(8)	3.6925(8)	3.393
Cg(2)	[1]	->	Cg(2)	[3456.01]	5.3322(11)	0.7363	-0.5851	0.3399	5.0603	85.16(9)	19.4	75.5	-1.3368(8)	-5.0293(8)	
Cg(2)	[1]	->	Cg(3)	[1655.01]	4.0900(11)	0.6457	0.6009	-0.4711	5.4384	9.19(9)	28.5	29.2	-3.5693(8)	3.5949(8)	
Cg(3)	[1]	->	Cg(1)	[1455.01]	3.7590(11)	0.7165	0.5631	-0.4117	-0.8900	5.72(9)	27.9	22.5	3.4720(8)	-3.3207(8)	
Cg(3)	[1]	->	Cg(2)	[1455.01]	4.0899(11)	0.7363	0.5851	-0.3399	0.0355	9.19(9)	29.2	28.5	3.5948(8)	-3.5693(8)	
Cg(3)	[1]	->	Cg(3)	[1455.01]	5.0147(12)	0.6457	0.6009	-0.4711	-1.0380	0.00(9)	49.8	49.8	3.2382(8)	-3.2382(8)	3.829
Cg(3)	[1]	->	Cg(3)	[1655.01]	5.0147(12)	0.6457	0.6009	-0.4711	5.4384	0.00(9)	49.8	49.8	-3.2383(8)	3.2383(8)	3.829
				Min or Max	3.759					0.0	18.2	75.5	-3.693	-5.029	

[1455] = -1+X,Y,Z
[1655] = 1+X,Y,Z
[3456] = -1/2+X,1/2-Y,1-Z

```
==========================================================================================================================

Analysis of Y-X...Cg(Pi-Ring) Interactions (X..Cg < 4.0 Ang. - Gamma <  30.0 Deg)
==========================================================================================================================

   Y--X(I)    Res(I)   Cg(J)  [   ARU(J)]      X..Cg  Transformed J-Plane P, Q, R, S  X-Perp Gamma      Y-X..Cg        Y..Cg Y-X,Pi

C(8)   -O(2)    [ 1] -> Cg(3)  [  1655.01]  3.5830(16)   0.6457 0.6009-0.4711   5.4384 -3.180  27.45    76.17(11)     3.498(2)   7.00
                                            ----------                                  ----------------------------------------------
                                 Min or Max  3.583                                      -3.180   27.4        76.17        3.498   7.00

[  1655] = 1+X,Y,Z

The Cg(I) refer to the Ring Centre-of-Gravity numbers given in () in the Ring-Analysis above
```

Cg(I)	x	y	z	Xo	Yo	Zo
Cg(1)	0.69218(16)	0.45847(4)	0.44001(3)	3.4711(8)	8.5675(7)	11.1923(7)
Cg(2)	1.00902(17)	0.35716(4)	0.45143(3)	5.0599(9)	6.6744(7)	11.4829(8)
Cg(3)	0.35035(16)	0.55380(4)	0.43008(3)	1.7569(8)	10.3489(7)	10.9398(7)

Analysis of Short Intra- and Inter-molecular Contacts , d(I-J) < R(I) + R(J) + Tolr, With Tolr = 0.2 Ang. (X - I...J) > 100. Deg.

Contact Radii :	C	H	N	O	S
(Angstrom)	1.70	1.20	1.55	1.52	1.80

Default Contact Radii are those given by A.Bondi, J.Phys.Chem. (1964),68,441. (or Coval. Rad. + 0.8 Ang. when not given)

* WARNING * : no Far-Reaching Conclusions should be drawn based on the Default Radii Assigned to Metals

Short "INTRA" Distances between two Atoms that are Separated by less than 4 Bonds are NOT Listed (Except for Potential D/A Contacts)

At(I)[1555.01]		At(J)	[ARU(J)]	D(I-J)	SumRad	Del	Type	X(I)	Y(I)	Z(I)	X(J)	Y(J)	Z(J)	X	X - I...J
S(1)	....	O(5)	[1455.01]	3.4332(16)	3.32	0.11		0.7261	0.4551	0.7425	0.2260	0.3381	0.7092	H(1)	172
S(1)	....	O(5)	[ ]	3.4321(16)	3.32	0.11	Intra	0.7261	0.4551	0.7425	1.2260	0.3381	0.7092		
S(1)	....	C(18)	[ ]	3.104(2)<<	3.50	-0.40	Intra	0.7261	0.4551	0.7425	1.0102	0.3353	0.6792	H(1)	100
S(1)	....	H(11)	[ ]	3.03	3.00	0.03	Intra	0.7261	0.4551	0.7425	1.1285	0.4370	0.6548		
S(1)	....	H(20)	[ ]	3.11	3.00	0.11	Intra	0.7261	0.4551	0.7425	0.3137	0.5752	0.7657	C(17)	105
S(2)	....	O(8)	[ ]	3.4342(14)	3.32	0.11	Intra	0.1101	0.7609	0.6200	0.3970	0.6461	0.7090	H(2)	120
S(2)	....	N(2)	[ ]	3.3273(16) <	3.35	-0.02	Intra	0.1101	0.7609	0.6200	0.3927	0.6148	0.5702		
S(2)	....	C(22)	[1455.01]	3.354(2) <	3.50	-0.15		0.1101	0.7609	0.6200	-0.5484	0.7297	0.6181	C(22)	151.08(7)
S(2)	....	C(23)	[ ]	3.1302(18)<<	3.50	-0.37	Intra	0.1101	0.7609	0.6200	0.3275	0.6158	0.6640		
S(2)	....	H(17)	[1455.01]	3.19	3.00	0.19		0.1101	0.7609	0.6200	-0.3263	0.6368	0.6237	C(22)	115
S(2)	....	H(18)	[1455.01]	3.01	3.00	0.01		0.1101	0.7609	0.6200	-0.4621	0.7485	0.5860	C(22)	151
S(2)	....	H(19)	[1455.01]	2.92 <	3.00	-0.08		0.1101	0.7609	0.6200	-0.4520	0.7496	0.6488	C(22)	154
S(2)	....	C(11)	[3466.01]	3.556(2)	3.50	0.06		0.1101	0.7609	0.6200	-0.2843	0.8873	0.5502	C(22)	136.67(7)
S(2)	....	H(9)	[3566.01]	3.19	3.00	0.19		0.1101	0.7609	0.6200	0.4930	0.8971	0.6168	H(2)	117
S(2)	....	H(14)	[4656.01]	3.09	3.00	0.09		0.1101	0.7609	0.6200	-0.1457	0.7737	0.7302	C(22)	116
														H(2)	132
O(1)	....	C(3)	[ ]	2.782(3)<<	3.22	-0.44	Intra	0.5950	0.4146	0.3417	0.9540	0.3342	0.4005		
O(1)	....	C(13)	[ ]	2.807(3)<<	3.22	-0.41	Intra	0.5950	0.4146	0.3417	0.2758	0.5254	0.3816		
O(1)	....	H(5)	[ ]	2.48<<	2.72	-0.24	Intra	0.5950	0.4146	0.3417	0.9134	0.3183	0.3659		
O(1)	....	H(10)	[ ]	2.52<<	2.72	-0.20	Intra	0.5950	0.4146	0.3417	0.2289	0.5068	0.3480		
O(1)	....	C(24)	[2564.01]	2.889(3)<<	3.22	-0.33		0.5950	0.4146	0.3417	0.2498	0.3774	0.2549	C(1)	151.47(17)
O(1)	....	H(20)	[2564.01]	2.82	2.72	0.10		0.5950	0.4146	0.3417	0.1862	0.4248	0.2657	C(1)	137
O(1)	....	H(21)	[2564.01]	2.62 <	2.72	-0.10		0.5950	0.4146	0.3417	0.4403	0.3800	0.2465	C(1)	171
O(1)	....	H(22)	[2564.01]	2.73	2.72	0.01		0.5950	0.4146	0.3417	0.2222	0.3433	0.2837	C(1)	138
O(2)	....	O(6)	[ ]	2.9539(18) <	3.04	-0.09	Intra	0.8653	0.5171	0.5299	0.7103	0.6690	0.5207	C(8)	104.90(11)
O(2)	....	N(1)	[ ]	2.8719(19) <	3.07	-0.20	Intra	0.8653	0.5171	0.5299	0.9272	0.3918	0.5941		
O(2)	....	N(2)	[ ]	3.162(2)	3.07	0.09	Intra	0.8653	0.5171	0.5299	0.3927	0.6148	0.5702	C(8)	103.59(12)
O(2)	....	C(6)	[ ]	2.844(2)<<	3.22	-0.38	Intra	0.8653	0.5171	0.5299	1.0606	0.3789	0.5033		
O(2)	....	C(10)	[ ]	2.845(2)<<	3.22	-0.38	Intra	0.8653	0.5171	0.5299	0.4304	0.5844	0.4778		
O(2)	....	C(10)	[1655.01]	3.372(2)	3.22	0.15		0.8653	0.5171	0.5299	1.4304	0.5844	0.4778		
O(2)	....	C(11)	[1655.01]	3.231(2)	3.22	0.01		0.8653	0.5171	0.5299	1.2157	0.6127	0.4498		
O(2)	....	C(15)	[ ]	2.749(2)<<	3.22	-0.47	Intra	0.8653	0.5171	0.5299	1.1287	0.3946	0.5597		
O(2)	....	C(20)	[ ]	2.648(2)<<	3.22	-0.57	Intra	0.8653	0.5171	0.5299	0.5274	0.6258	0.5251		

O(2)	....	H(3)	[ ]	2.92	2.72	0.20	Intra	0.8653	0.5171	0.5299	0.7661	0.3812	0.5828		
O(2)	....	H(4)	[1655.01]	2.55 <	2.72	-0.17		0.8653	0.5171	0.5299	1.2507	0.5871	0.5702	C(8)	147
O(3)	....	N(1)	[1655.01]	2.890(2) <	3.07	-0.18		1.3628	0.4027	0.5726	1.9272	0.3917	0.5941	C(15)	167.90(15)
O(3)	....	C(5)	[ ]	2.918(3)<<	3.22	-0.30	Intra	1.3628	0.4027	0.5726	1.1909	0.3199	0.4814		
O(3)	....	C(8)	[1655.01]	3.339(2)	3.22	0.12		1.3628	0.4027	0.5726	1.7736	0.4884	0.4907	C(15)	118.77(13)
O(3)	....	C(16)	[ ]	2.795(2)<<	3.22	-0.42	Intra	1.3628	0.4027	0.5726	0.9681	0.4058	0.6501		
O(3)	....	C(17)	[1655.01]	3.198(2) <	3.22	-0.02		1.3628	0.4027	0.5726	1.7230	0.4439	0.6719	C(15)	141.36(13)
O(3)	....	H(3)	[1655.01]	2.08<<	2.72	-0.64		1.3628	0.4027	0.5726	1.7661	0.3812	0.5828	C(15)	160
O(3)	....	H(7)	[ ]	2.71 <	2.72	-0.01	Intra	1.3628	0.4027	0.5726	1.3175	0.2945	0.5020		
O(3)	....	H(11)	[ ]	2.48<<	2.72	-0.24	Intra	1.3628	0.4027	0.5726	1.1285	0.4370	0.6548		
O(3)	....	H(12)	[1655.01]	2.49<<	2.72	-0.23		1.3628	0.4027	0.5726	1.5621	0.4163	0.6619	C(15)	130
O(4)	....	N(1)	[ ]	2.901(2) <	3.07	-0.17	Intra	0.8638	0.2846	0.6757	0.9272	0.3918	0.5941		
O(4)	....	C(17)	[ ]	3.060(2) <	3.22	-0.16	Intra	0.8638	0.2846	0.6757	0.7230	0.4439	0.6719		
O(4)	....	C(19)	[1455.01]	3.389(3)	3.22	0.17		0.8638	0.2846	0.6757	0.2760	0.2756	0.7412	C(18)	122.31(14)
O(4)	....	C(19)	[ ]	2.659(3)<<	3.22	-0.56	Intra	0.8638	0.2846	0.6757	1.2760	0.2756	0.7412		
O(4)	....	H(12)	[ ]	2.91	2.72	0.19	Intra	0.8638	0.2846	0.6757	0.5621	0.4163	0.6619		
O(4)	....	H(14)	[ ]	2.79	2.72	0.07	Intra	0.8638	0.2846	0.6757	1.1457	0.2737	0.7698		
O(4)	....	H(15)	[1455.01]	2.89	2.72	0.17		0.8638	0.2846	0.6757	0.4564	0.2784	0.7559	C(18)	114
O(4)	....	H(16)	[ ]	2.48<<	2.72	-0.24	Intra	0.8638	0.2846	0.6757	1.2600	0.2325	0.7195		
O(4)	....	C(4)	[3456.01]	3.306(3)	3.22	0.09		0.8638	0.2846	0.6757	0.6399	0.2024	0.5695	C(18)	129.36(13)
O(4)	....	H(6)	[3456.01]	2.56 <	2.72	-0.16		0.8638	0.2846	0.6757	0.7316	0.2425	0.5837	C(18)	118
O(4)	....	H(22)	[4646.01]	2.70 <	2.72	-0.02		0.8638	0.2846	0.6757	0.7222	0.1567	0.7163	C(18)	146
O(5)	....	S(1)	[ ]	3.4321(16)	3.32	0.11	Intra	1.2260	0.3381	0.7092	0.7261	0.4551	0.7425	C(19)	120.12(13)
O(5)	....	S(1)	[1655.01]	3.4332(16)	3.32	0.11		1.2260	0.3381	0.7092	1.7262	0.4551	0.7425	C(18)	139.88(12)
														C(19)	104.38(12)
O(5)	....	C(17)	[ ]	3.342(2)	3.22	0.12	Intra	1.2260	0.3381	0.7092	0.7230	0.4439	0.6719	C(19)	140.23(13)
O(5)	....	C(17)	[1655.01]	3.319(2)	3.22	0.10		1.2260	0.3381	0.7092	1.7230	0.4439	0.6719	C(18)	118.23(12)
														C(19)	120.78(13)
O(5)	....	H(11)	[ ]	2.36<<	2.72	-0.36	Intra	1.2260	0.3381	0.7092	1.1285	0.4370	0.6548	C(19)	177
O(5)	....	H(12)	[1655.01]	2.53 <	2.72	-0.19		1.2260	0.3381	0.7092	1.5621	0.4163	0.6619	C(18)	107
														C(19)	128
O(6)	....	O(2)	[ ]	2.9539(18) <	3.04	-0.09	Intra	0.7103	0.6690	0.5207	0.8653	0.5171	0.5299		
O(6)	....	C(9)	[ ]	3.242(2)	3.22	0.02	Intra	0.7103	0.6690	0.5207	0.5579	0.5227	0.4591		
O(6)	....	C(11)	[ ]	3.242(2)	3.22	0.02	Intra	0.7103	0.6690	0.5207	0.2157	0.6127	0.4498		
O(6)	....	C(11)	[1655.01]	3.284(2)	3.22	0.06		0.7103	0.6690	0.5207	1.2157	0.6127	0.4498	C(20)	114.58(12)
O(6)	....	C(21)	[ ]	2.773(2)<<	3.22	-0.45	Intra	0.7103	0.6690	0.5207	0.4806	0.6483	0.6187		
O(6)	....	C(22)	[ ]	3.018(2)<<	3.22	-0.20	Intra	0.7103	0.6690	0.5207	0.4516	0.7297	0.6181		
O(6)	....	H(2)	[1655.01]	2.64 <	2.72	-0.08		0.7103	0.6690	0.5207	1.0355	0.7408	0.5827	C(20)	138
O(6)	....	H(8)	[1655.01]	2.54 <	2.72	-0.18		0.7103	0.6690	0.5207	1.1208	0.6525	0.4636	C(20)	125
O(6)	....	H(17)	[ ]	2.69 <	2.72	-0.03	Intra	0.7103	0.6690	0.5207	0.6737	0.6368	0.6237		
O(6)	....	H(18)	[ ]	2.39<<	2.72	-0.33	Intra	0.7103	0.6690	0.5207	0.5379	0.7485	0.5860		
O(7)	....	N(2)	[ ]	2.687(2)<<	3.07	-0.38	Intra	0.1614	0.5693	0.6594	0.3927	0.6148	0.5702		
O(7)	....	C(16)	[1455.01]	3.214(2) <	3.22	-0.01		0.1614	0.5693	0.6594	-0.0319	0.4058	0.6501	C(23)	153.86(13)
O(7)	....	C(17)	[1455.01]	3.229(2)	3.22	0.01		0.1614	0.5693	0.6594	-0.2770	0.4439	0.6719	C(23)	168.75(12)
O(7)	....	C(24)	[ ]	2.664(2)<<	3.22	-0.56	Intra	0.1614	0.5693	0.6594	0.2502	0.6226	0.7549		
O(7)	....	H(4)	[ ]	2.34<<	2.72	-0.38	Intra	0.1614	0.5693	0.6594	0.2507	0.5871	0.5702		
O(7)	....	H(11)	[1455.01]	2.48<<	2.72	-0.24		0.1614	0.5693	0.6594	0.1285	0.4370	0.6548	C(23)	140
O(7)	....	H(13)	[1455.01]	2.69 <	2.72	-0.03		0.1614	0.5693	0.6594	-0.2907	0.4916	0.6552	C(23)	166

O(7)	....	H(17)	[1455.01]	2.90	2.72	0.18		0.1614	0.5693	0.6594	-0.3263	0.6368	0.6237	C(23)	107
O(7)	....	H(20)	[ ]	2.81	2.72	0.09	Intra	0.1614	0.5693	0.6594	0.3137	0.5752	0.7657		
O(7)	....	H(21)	[ ]	2.46<<	2.72	-0.26	Intra	0.1614	0.5693	0.6594	0.0597	0.6200	0.7465		
O(8)	....	S(2)	[ ]	3.4342(14)	3.32	0.11	Intra	0.3970	0.6461	0.7090	0.1101	0.7609	0.6200	C(24)	120.54(13)
O(8)	....	C(22)	[ ]	2.802(2)<<	3.22	-0.42	Intra	0.3970	0.6461	0.7090	0.4516	0.7297	0.6181	C(24)	152.06(14)
O(8)	....	H(17)	[ ]	2.58 <	2.72	-0.14	Intra	0.3970	0.6461	0.7090	0.6737	0.6368	0.6237	C(24)	158
O(8)	....	H(19)	[ ]	2.58 <	2.72	-0.14	Intra	0.3970	0.6461	0.7090	0.5480	0.7496	0.6488	C(24)	149
O(8)	....	C(19)	[4756.01]	3.187(3) <	3.22	-0.03		0.3970	0.6461	0.7090	0.7240	0.7756	0.7588	C(23)	143.63(12)
O(8)	....	H(15)	[4756.01]	2.73	2.72	0.01		0.3970	0.6461	0.7090	0.5436	0.7784	0.7441	C(23)	138
N(1)	....	O(2)	[ ]	2.8719(19) <	3.07	-0.20	Intra	0.9272	0.3918	0.5941	0.8653	0.5171	0.5299	C(16)	115.00(10)
N(1)	....	O(3)	[1455.01]	2.890(2) <	3.07	-0.18		0.9272	0.3918	0.5941	0.3628	0.4027	0.5726	C(15)	127.73(12)
														C(16)	108.01(11)
N(1)	....	O(4)	[ ]	2.901(2) <	3.07	-0.17	Intra	0.9272	0.3918	0.5941	0.8638	0.2846	0.6757	C(15)	125.35(12)
N(1)	....	C(5)	[ ]	3.431(3)	3.25	0.18	Intra	0.9272	0.3918	0.5941	1.1909	0.3199	0.4814	C(16)	146.08(12)
N(1)	....	C(7)	[ ]	3.154(2) <	3.25	-0.10	Intra	0.9272	0.3918	0.5941	0.8819	0.4181	0.4720	C(16)	160.26(11)
N(1)	....	C(8)	[ ]	3.283(2)	3.25	0.03	Intra	0.9272	0.3918	0.5941	0.7736	0.4884	0.4907	C(16)	135.58(11)
N(1)	....	H(12)	[ ]	2.56 <	2.75	-0.19	Intra	0.9272	0.3918	0.5941	0.5621	0.4163	0.6619	C(15)	167
N(1)	....	H(13)	[ ]	2.66 <	2.75	-0.09	Intra	0.9272	0.3918	0.5941	0.7093	0.4916	0.6552	C(15)	132
N(2)	....	S(2)	[ ]	3.3273(16) <	3.35	-0.02	Intra	0.3927	0.6148	0.5702	0.1101	0.7609	0.6200	C(20)	114.37(11)
N(2)	....	O(2)	[ ]	3.162(2)	3.07	0.09	Intra	0.3927	0.6148	0.5702	0.8653	0.5171	0.5299	C(21)	107.27(11)
														H(4)	106
N(2)	....	O(7)	[ ]	2.687(2)<<	3.07	-0.38	Intra	0.3927	0.6148	0.5702	0.1614	0.5693	0.6594	C(20)	169.79(12)
N(2)	....	C(9)	[ ]	3.409(2)	3.25	0.16	Intra	0.3927	0.6148	0.5702	0.5579	0.5227	0.4591	C(21)	147.98(12)
N(2)	....	C(11)	[ ]	3.188(2) <	3.25	-0.06	Intra	0.3927	0.6148	0.5702	0.2157	0.6127	0.4498	C(21)	154.87(11)
N(2)	....	H(18)	[ ]	2.63 <	2.75	-0.12	Intra	0.3927	0.6148	0.5702	0.5379	0.7485	0.5860	H(4)	141
C(1)	....	C(8)	[ ]	2.925(2)<<	3.40	-0.48	Intra	0.6300	0.4329	0.3870	0.7736	0.4884	0.4907	O(1)	172.25(16)
C(1)	....	H(5)	[ ]	2.63<<	2.90	-0.27	Intra	0.6300	0.4329	0.3870	0.9134	0.3183	0.3659	C(14)	168
C(1)	....	H(10)	[ ]	2.63<<	2.90	-0.27	Intra	0.6300	0.4329	0.3870	0.2289	0.5068	0.3480	C(2)	167
C(2)	....	C(5)	[ ]	2.765(3)<<	3.40	-0.63	Intra	0.8268	0.3944	0.4209	1.1909	0.3199	0.4814	C(1)	178.33(13)
C(2)	....	C(9)	[ ]	2.919(2)<<	3.40	-0.48	Intra	0.8268	0.3944	0.4209	0.5579	0.5227	0.4591	C(3)	177.56(14)
C(2)	....	C(13)	[1655.01]	3.474(3)	3.40	0.07		0.8268	0.3944	0.4209	1.2758	0.5254	0.3816		
C(3)	....	O(1)	[ ]	2.782(3)<<	3.22	-0.44	Intra	0.9540	0.3342	0.4005	0.5950	0.4146	0.3417	C(4)	176.85(15)
C(3)	....	C(6)	[ ]	2.796(3)<<	3.40	-0.60	Intra	0.9540	0.3342	0.4005	1.0606	0.3789	0.5033	H(5)	178
C(4)	....	C(7)	[ ]	2.803(3)<<	3.40	-0.60	Intra	1.1399	0.2976	0.4305	0.8819	0.4181	0.4720	H(6)	178
C(4)	....	O(4)	[3556.01]	3.306(3)	3.22	0.09		1.1399	0.2976	0.4305	1.3638	0.2154	0.3243	C(5)	147.37(16)
C(4)	....	H(7)	[3456.01]	2.92	2.90	0.02		1.1399	0.2976	0.4305	0.8175	0.2055	0.4980	C(3)	104
C(5)	....	O(3)	[ ]	2.918(3)<<	3.22	-0.30	Intra	1.1909	0.3199	0.4814	1.3628	0.4027	0.5726	C(4)	163.27(15)
C(5)	....	N(1)	[ ]	3.431(3)	3.25	0.18	Intra	1.1909	0.3199	0.4814	0.9272	0.3918	0.5941	C(4)	145.93(15)
C(5)	....	C(2)	[ ]	2.765(3)<<	3.40	-0.63	Intra	1.1909	0.3199	0.4814	0.8268	0.3944	0.4209	H(7)	179
C(5)	....	H(7)	[3456.01]	2.87 <	2.90	-0.03		1.1909	0.3199	0.4814	0.8175	0.2055	0.4980	C(6)	103
C(6)	....	O(2)	[ ]	2.844(2)<<	3.22	-0.38	Intra	1.0606	0.3789	0.5033	0.8653	0.5171	0.5299	C(5)	166.60(14)
C(6)	....	C(3)	[ ]	2.796(3)<<	3.40	-0.60	Intra	1.0606	0.3789	0.5033	0.9540	0.3342	0.4005	C(15)	173.62(13)
C(6)	....	H(3)	[ ]	2.51<<	2.90	-0.39	Intra	1.0606	0.3789	0.5033	0.7661	0.3812	0.5828	C(5)	128
C(7)	....	N(1)	[ ]	3.154(2) <	3.25	-0.10	Intra	0.8819	0.4181	0.4720	0.9272	0.3918	0.5941	C(2)	151.51(12)
C(7)	....	C(4)	[ ]	2.803(3)<<	3.40	-0.60	Intra	0.8819	0.4181	0.4720	1.1399	0.2976	0.4305	C(8)	172.16(14)
C(7)	....	C(14)	[ ]	2.916(3)<<	3.40	-0.48	Intra	0.8819	0.4181	0.4720	0.4829	0.4943	0.4103	C(6)	176.31(14)
C(7)	....	H(3)	[ ]	2.96	2.90	0.06	Intra	0.8819	0.4181	0.4720	0.7661	0.3812	0.5828	C(2)	140
C(8)	....	O(3)	[1455.01]	3.339(2)	3.22	0.12		0.7736	0.4884	0.4907	0.3628	0.4027	0.5726		

C(8)	....	N(1)	[ ]	3.283(2)	3.25	0.03	Intra	0.7736	0.4884	0.4907	0.9272	0.3918	0.5941	C(9)	146.94(12)
C(8)	....	C(1)	[ ]	2.925(2)<<	3.40	-0.48	Intra	0.7736	0.4884	0.4907	0.6300	0.4329	0.3870	O(2)	169.87(14)
C(8)	....	C(11)	[1655.01]	3.375(3) <	3.40	-0.03		0.7736	0.4884	0.4907	1.2157	0.6127	0.4498	C(7)	105.51(12)
C(8)	....	C(12)	[1655.01]	3.406(3)	3.40	0.01		0.7736	0.4884	0.4907	1.1394	0.5832	0.4019		
C(8)	....	C(15)	[ ]	3.053(3)<<	3.40	-0.35	Intra	0.7736	0.4884	0.4907	1.1287	0.3946	0.5597	C(9)	168.19(13)
C(8)	....	C(20)	[ ]	2.979(2)<<	3.40	-0.42	Intra	0.7736	0.4884	0.4907	0.5274	0.6258	0.5251	C(7)	176.60(14)
C(8)	....	H(3)	[ ]	3.08	2.90	0.18	Intra	0.7736	0.4884	0.4907	0.7661	0.3812	0.5828	C(9)	133
C(9)	....	O(6)	[ ]	3.242(2)	3.22	0.02	Intra	0.5579	0.5227	0.4591	0.7103	0.6690	0.5207	C(14)	144.10(12)
C(9)	....	N(2)	[ ]	3.409(2)	3.25	0.16	Intra	0.5579	0.5227	0.4591	0.3927	0.6148	0.5702	C(14)	149.29(14)
C(9)	....	C(2)	[ ]	2.919(2)<<	3.40	-0.48	Intra	0.5579	0.5227	0.4591	0.8268	0.3944	0.4209	C(10)	179.52(13)
C(9)	....	C(12)	[ ]	2.793(3)<<	3.40	-0.61	Intra	0.5579	0.5227	0.4591	0.1394	0.5832	0.4019	C(8)	177.68(13)
C(9)	....	C(12)	[1655.01]	3.449(3)	3.40	0.05		0.5579	0.5227	0.4591	1.1394	0.5832	0.4019	C(10)	105.04(12)
C(10)	....	O(2)	[1455.01]	3.372(2)	3.22	0.15		0.4304	0.5844	0.4778	-0.1347	0.5171	0.5299	C(9)	102.13(12)
C(10)	....	O(2)	[ ]	2.845(2)<<	3.22	-0.38	Intra	0.4304	0.5844	0.4778	0.8653	0.5171	0.5299	C(11)	175.59(13)
C(10)	....	C(13)	[ ]	2.795(3)<<	3.40	-0.61	Intra	0.4304	0.5844	0.4778	0.2758	0.5254	0.3816	C(20)	171.47(13)
C(10)	....	H(4)	[ ]	2.52<<	2.90	-0.38	Intra	0.4304	0.5844	0.4778	0.2507	0.5871	0.5702	C(9)	120
														C(11)	101
C(11)	....	O(2)	[1455.01]	3.231(2)	3.22	0.01		0.2157	0.6127	0.4498	-0.1347	0.5171	0.5299	C(12)	100.56(12)
C(11)	....	O(6)	[1455.01]	3.284(2)	3.22	0.06		0.2157	0.6127	0.4498	-0.2897	0.6690	0.5207	C(10)	115.91(12)
														C(12)	113.30(13)
C(11)	....	O(6)	[ ]	3.242(2)	3.22	0.02	Intra	0.2157	0.6127	0.4498	0.7103	0.6690	0.5207	C(12)	145.72(14)
C(11)	....	N(2)	[ ]	3.188(2) <	3.25	-0.06	Intra	0.2157	0.6127	0.4498	0.3927	0.6148	0.5702	C(12)	157.29(14)
C(11)	....	C(8)	[1455.01]	3.375(3) <	3.40	-0.03		0.2157	0.6127	0.4498	-0.2264	0.4884	0.4907		
C(11)	....	C(14)	[ ]	2.775(3)<<	3.40	-0.63	Intra	0.2157	0.6127	0.4498	0.4829	0.4943	0.4103	H(8)	179
C(11)	....	S(2)	[3566.01]	3.556(2)	3.50	0.06		0.2157	0.6127	0.4498	0.6101	0.7391	0.3800		
C(11)	....	H(18)	[3466.01]	2.89 <	2.90	-0.01		0.2157	0.6127	0.4498	0.0379	0.7515	0.4140	C(10)	138
C(12)	....	C(8)	[1455.01]	3.406(3)	3.40	0.01		0.1394	0.5832	0.4019	-0.2264	0.4884	0.4907		
C(12)	....	C(9)	[1455.01]	3.449(3)	3.40	0.05		0.1394	0.5832	0.4019	-0.4421	0.5227	0.4591	C(13)	108.72(13)
C(12)	....	C(9)	[ ]	2.793(3)<<	3.40	-0.61	Intra	0.1394	0.5832	0.4019	0.5579	0.5227	0.4591	H(9)	178
C(13)	....	O(1)	[ ]	2.807(3)<<	3.22	-0.41	Intra	0.2758	0.5254	0.3816	0.5950	0.4146	0.3417	C(12)	174.93(15)
C(13)	....	C(2)	[1455.01]	3.474(3)	3.40	0.07		0.2758	0.5254	0.3816	-0.1732	0.3944	0.4209		
C(13)	....	C(10)	[ ]	2.795(3)<<	3.40	-0.61	Intra	0.2758	0.5254	0.3816	0.4304	0.5844	0.4778	H(10)	177
C(14)	....	C(7)	[ ]	2.916(3)<<	3.40	-0.48	Intra	0.4829	0.4943	0.4103	0.8819	0.4181	0.4720	C(13)	174.60(14)
C(14)	....	C(11)	[ ]	2.775(3)<<	3.40	-0.63	Intra	0.4829	0.4943	0.4103	0.2157	0.6127	0.4498	C(1)	177.46(13)
C(15)	....	O(2)	[ ]	2.749(2)<<	3.22	-0.47	Intra	1.1287	0.3946	0.5597	0.8653	0.5171	0.5299	O(3)	115.48(14)
C(15)	....	C(8)	[ ]	3.053(3)<<	3.40	-0.35	Intra	1.1287	0.3946	0.5597	0.7736	0.4884	0.4907	O(3)	129.83(14)
C(15)	....	C(18)	[ ]	3.291(3) <	3.40	-0.11	Intra	1.1287	0.3946	0.5597	1.0102	0.3353	0.6792	C(6)	140.81(12)
C(15)	....	H(7)	[ ]	2.56<<	2.90	-0.34	Intra	1.1287	0.3946	0.5597	1.3175	0.2945	0.5020	N(1)	129
C(15)	....	H(11)	[ ]	2.55<<	2.90	-0.35	Intra	1.1287	0.3946	0.5597	1.1285	0.4370	0.6548	C(6)	165
C(16)	....	O(3)	[ ]	2.795(2)<<	3.22	-0.42	Intra	0.9681	0.4058	0.6501	1.3628	0.4027	0.5726	C(17)	146.81(12)
														C(18)	103.07(12)
C(16)	....	O(7)	[1655.01]	3.214(2) <	3.22	-0.01		0.9681	0.4058	0.6501	1.1614	0.5693	0.6594	N(1)	106.53(10)
														C(18)	138.04(12)
C(16)	....	H(1)	[ ]	2.97	2.90	0.07	Intra	0.9681	0.4058	0.6501	0.8819	0.4911	0.7471	N(1)	152
C(16)	....	H(12)	[1655.01]	3.00	2.90	0.10		0.9681	0.4058	0.6501	1.5621	0.4163	0.6619	N(1)	104
														C(17)	137
C(17)	....	O(3)	[1455.01]	3.198(2) <	3.22	-0.02		0.7230	0.4439	0.6719	0.3628	0.4027	0.5726	S(1)	144.76(10)
C(17)	....	O(4)	[ ]	3.060(2) <	3.22	-0.16	Intra	0.7230	0.4439	0.6719	0.8638	0.2846	0.6757	H(13)	155

C(17)	....	O(5)	[1455.01]	3.319(2)	3.22	0.10		0.7230	0.4439	0.6719	0.2260	0.3381	0.7092	C(16)	115.55(11)
														H(13)	127
C(17)	....	O(5)	[ ]	3.342(2)	3.22	0.12	Intra	0.7230	0.4439	0.6719	1.2260	0.3381	0.7092	H(12)	112
														H(13)	135
C(17)	....	O(7)	[1655.01]	3.229(2)	3.22	0.01		0.7230	0.4439	0.6719	1.1614	0.5693	0.6594	H(12)	155
C(17)	....	H(3)	[ ]	2.56<<	2.90	-0.34	Intra	0.7230	0.4439	0.6719	0.7661	0.3812	0.5828	S(1)	159
C(17)	....	H(11)	[1455.01]	3.02	2.90	0.12		0.7230	0.4439	0.6719	0.1285	0.4370	0.6548	C(16)	136
C(18)	....	S(1)	[ ]	3.104(2)<<	3.50	-0.40	Intra	1.0102	0.3353	0.6792	0.7261	0.4551	0.7425	O(4)	109.03(14)
C(18)	....	C(15)	[ ]	3.291(3) <	3.40	-0.11	Intra	1.0102	0.3353	0.6792	1.1287	0.3946	0.5597	O(4)	107.91(13)
														O(5)	111.74(12)
C(18)	....	H(3)	[ ]	2.87 <	2.90	-0.03	Intra	1.0102	0.3353	0.6792	0.7661	0.3812	0.5828	O(5)	146
C(18)	....	H(12)	[ ]	2.75 <	2.90	-0.15	Intra	1.0102	0.3353	0.6792	0.5621	0.4163	0.6619	O(5)	138
C(18)	....	H(14)	[ ]	2.66<<	2.90	-0.24	Intra	1.0102	0.3353	0.6792	1.1457	0.2737	0.7698	C(16)	146
C(18)	....	H(16)	[ ]	2.51<<	2.90	-0.39	Intra	1.0102	0.3353	0.6792	1.2600	0.2325	0.7195	C(16)	158
C(19)	....	O(4)	[ ]	2.659(3)<<	3.22	-0.56	Intra	1.2760	0.2756	0.7412	0.8638	0.2846	0.6757	H(15)	162
C(19)	....	O(4)	[1655.01]	3.389(3)	3.22	0.17		1.2760	0.2756	0.7412	1.8638	0.2846	0.6757	H(14)	161
C(19)	....	O(8)	[4746.01]	3.187(3) <	3.22	-0.03		1.2760	0.2756	0.7412	1.6030	0.1461	0.7910	O(5)	157.98(16)
C(19)	....	H(19)	[4746.01]	2.98	2.90	0.08		1.2760	0.2756	0.7412	1.4520	0.2496	0.8512	O(5)	136
														H(16)	115
C(20)	....	O(2)	[ ]	2.648(2)<<	3.22	-0.57	Intra	0.5274	0.6258	0.5251	0.8653	0.5171	0.5299		
C(20)	....	C(8)	[ ]	2.979(2)<<	3.40	-0.42	Intra	0.5274	0.6258	0.5251	0.7736	0.4884	0.4907	O(6)	103.34(13)
														N(2)	109.06(11)
C(20)	....	C(22)	[ ]	3.084(3)<<	3.40	-0.32	Intra	0.5274	0.6258	0.5251	0.4516	0.7297	0.6181	C(10)	153.32(14)
C(20)	....	H(8)	[ ]	2.62<<	2.90	-0.28	Intra	0.5274	0.6258	0.5251	0.1208	0.6525	0.4636	O(6)	114
C(20)	....	H(17)	[ ]	2.62<<	2.90	-0.28	Intra	0.5274	0.6258	0.5251	0.6737	0.6368	0.6237	C(10)	153
C(20)	....	H(18)	[ ]	2.77 <	2.90	-0.13	Intra	0.5274	0.6258	0.5251	0.5379	0.7485	0.5860	C(10)	151
C(21)	....	O(6)	[ ]	2.773(2)<<	3.22	-0.45	Intra	0.4806	0.6483	0.6187	0.7103	0.6690	0.5207	C(23)	162.19(12)
C(21)	....	H(2)	[ ]	2.97	2.90	0.07	Intra	0.4806	0.6483	0.6187	0.0355	0.7408	0.5827	H(17)	153
C(22)	....	S(2)	[1655.01]	3.354(2) <	3.50	-0.15		0.4516	0.7297	0.6181	1.1101	0.7609	0.6200	S(2)	151.08(8)
C(22)	....	O(6)	[ ]	3.018(2)<<	3.22	-0.20	Intra	0.4516	0.7297	0.6181	0.7103	0.6690	0.5207	S(2)	123.31(10)
														H(19)	125
C(22)	....	O(8)	[ ]	2.802(2)<<	3.22	-0.42	Intra	0.4516	0.7297	0.6181	0.3970	0.6461	0.7090	H(18)	157
C(22)	....	C(20)	[ ]	3.084(3)<<	3.40	-0.32	Intra	0.4516	0.7297	0.6181	0.5274	0.6258	0.5251	S(2)	109.86(9)
														H(19)	141
C(22)	....	H(2)	[1655.01]	3.07	2.90	0.17		0.4516	0.7297	0.6181	1.0355	0.7408	0.5827	S(2)	153
C(22)	....	H(4)	[ ]	3.10	2.90	0.20	Intra	0.4516	0.7297	0.6181	0.2507	0.5871	0.5702	H(19)	142
C(23)	....	S(2)	[ ]	3.1302(18)<<	3.50	-0.37	Intra	0.3275	0.6158	0.6640	0.1101	0.7609	0.6200	O(7)	110.32(13)
C(23)	....	H(4)	[ ]	2.48<<	2.90	-0.42	Intra	0.3275	0.6158	0.6640	0.2507	0.5871	0.5702	O(8)	165
C(23)	....	H(13)	[ ]	3.02	2.90	0.12	Intra	0.3275	0.6158	0.6640	0.7093	0.4916	0.6552	O(8)	103
C(23)	....	H(19)	[ ]	2.76 <	2.90	-0.14	Intra	0.3275	0.6158	0.6640	0.5480	0.7496	0.6488	O(7)	156
C(23)	....	H(20)	[ ]	2.70<<	2.90	-0.20	Intra	0.3275	0.6158	0.6640	0.3137	0.5752	0.7657	C(21)	149
C(23)	....	H(21)	[ ]	2.49<<	2.90	-0.41	Intra	0.3275	0.6158	0.6640	0.0597	0.6200	0.7465	C(21)	154
C(24)	....	O(7)	[ ]	2.664(2)<<	3.22	-0.56	Intra	0.2502	0.6226	0.7549	0.1614	0.5693	0.6594	H(22)	161
C(24)	....	H(1)	[1455.01]	3.08	2.90	0.18		0.2502	0.6226	0.7549	-0.1181	0.4911	0.7471	O(8)	120
														H(22)	131
C(24)	....	O(1)	[2565.01]	2.889(3)<<	3.22	-0.33		0.2502	0.6226	0.7549	-0.0950	0.5854	0.8417	O(8)	173.19(17)
H(1)	....	C(16)	[ ]	2.97	2.90	0.07	Intra	0.8819	0.4911	0.7471	0.9681	0.4058	0.6501		
H(1)	....	C(24)	[1655.01]	3.08	2.90	0.18		0.8819	0.4911	0.7471	1.2502	0.6226	0.7549	S(1)	167

H(1)	....	H(13)	[ ]	2.49	2.40	0.09	Intra	0.8819	0.4911	0.7471	0.7093	0.4916	0.6552		
H(1)	....	H(21)	[1655.01]	2.57	2.40	0.17		0.8819	0.4911	0.7471	1.0597	0.6200	0.7465	S(1)	150
H(2)	....	O(6)	[1455.01]	2.64 <	2.72	-0.08		0.0355	0.7408	0.5827	-0.2897	0.6690	0.5207	S(2)	156
H(2)	....	C(21)	[ ]	2.97	2.90	0.07	Intra	0.0355	0.7408	0.5827	0.4806	0.6483	0.6187		
H(2)	....	C(22)	[1455.01]	3.07	2.90	0.17		0.0355	0.7408	0.5827	-0.5484	0.7297	0.6181		
H(2)	....	H(18)	[1455.01]	2.50	2.40	0.10		0.0355	0.7408	0.5827	-0.4621	0.7485	0.5860	S(2)	107
H(2)	....	H(18)	[ ]	2.52	2.40	0.12	Intra	0.0355	0.7408	0.5827	0.5379	0.7485	0.5860		
H(3)	....	O(2)	[ ]	2.92	2.72	0.20	Intra	0.7661	0.3812	0.5828	0.8653	0.5171	0.5299		
H(3)	....	O(3)	[1455.01]	2.08<<	2.72	-0.64		0.7661	0.3812	0.5828	0.3628	0.4027	0.5726	N(1)	153
H(3)	....	C(6)	[ ]	2.51<<	2.90	-0.39	Intra	0.7661	0.3812	0.5828	1.0606	0.3789	0.5033		
H(3)	....	C(7)	[ ]	2.96	2.90	0.06	Intra	0.7661	0.3812	0.5828	0.8819	0.4181	0.4720		
H(3)	....	C(8)	[ ]	3.08	2.90	0.18	Intra	0.7661	0.3812	0.5828	0.7736	0.4884	0.4907		
H(3)	....	C(17)	[ ]	2.56<<	2.90	-0.34	Intra	0.7661	0.3812	0.5828	0.7230	0.4439	0.6719		
H(3)	....	C(18)	[ ]	2.87 <	2.90	-0.03	Intra	0.7661	0.3812	0.5828	1.0102	0.3353	0.6792		
H(3)	....	H(12)	[ ]	2.35 <	2.40	-0.05	Intra	0.7661	0.3812	0.5828	0.5621	0.4163	0.6619		
H(3)	....	H(6)	[3456.01]	2.60	2.40	0.20		0.7661	0.3812	0.5828	0.7316	0.2425	0.5837	N(1)	106
H(4)	....	O(2)	[1455.01]	2.55 <	2.72	-0.17		0.2507	0.5871	0.5702	-0.1347	0.5171	0.5299	N(2)	156
H(4)	....	O(7)	[ ]	2.34<<	2.72	-0.38	Intra	0.2507	0.5871	0.5702	0.1614	0.5693	0.6594	N(2)	104
H(4)	....	C(10)	[ ]	2.52<<	2.90	-0.38	Intra	0.2507	0.5871	0.5702	0.4304	0.5844	0.4778		
H(4)	....	C(22)	[ ]	3.10	2.90	0.20	Intra	0.2507	0.5871	0.5702	0.4516	0.7297	0.6181		
H(4)	....	C(23)	[ ]	2.48<<	2.90	-0.42	Intra	0.2507	0.5871	0.5702	0.3275	0.6158	0.6640		
H(5)	....	O(1)	[ ]	2.48<<	2.72	-0.24	Intra	0.9134	0.3183	0.3659	0.5950	0.4146	0.3417		
H(5)	....	C(1)	[ ]	2.63<<	2.90	-0.27	Intra	0.9134	0.3183	0.3659	0.6300	0.4329	0.3870		
H(5)	....	H(6)	[ ]	2.34 <	2.40	-0.06	Intra	0.9134	0.3183	0.3659	1.2316	0.2575	0.4163		
H(5)	....	H(16)	[3456.01]	2.49	2.40	0.09		0.9134	0.3183	0.3659	0.7600	0.2675	0.2805	C(3)	173
H(6)	....	H(5)	[ ]	2.34 <	2.40	-0.06	Intra	1.2316	0.2575	0.4163	0.9134	0.3183	0.3659		
H(6)	....	H(7)	[ ]	2.33 <	2.40	-0.07	Intra	1.2316	0.2575	0.4163	1.3175	0.2945	0.5020		
H(6)	....	O(4)	[3556.01]	2.56 <	2.72	-0.16		1.2316	0.2575	0.4163	1.3638	0.2154	0.3243	C(4)	136
H(6)	....	H(3)	[3556.01]	2.60	2.40	0.20		1.2316	0.2575	0.4163	1.2661	0.1188	0.4172	C(4)	145
H(7)	....	O(3)	[ ]	2.71 <	2.72	-0.01	Intra	1.3175	0.2945	0.5020	1.3628	0.4027	0.5726		
H(7)	....	C(15)	[ ]	2.56<<	2.90	-0.34	Intra	1.3175	0.2945	0.5020	1.1287	0.3946	0.5597		
H(7)	....	H(6)	[ ]	2.33 <	2.40	-0.07	Intra	1.3175	0.2945	0.5020	1.2316	0.2575	0.4163		
H(7)	....	C(4)	[3556.01]	2.92	2.90	0.02		1.3175	0.2945	0.5020	1.6399	0.2024	0.5695	C(5)	171
H(7)	....	C(5)	[3556.01]	2.87 <	2.90	-0.03		1.3175	0.2945	0.5020	1.6909	0.1801	0.5186	C(5)	153
H(8)	....	O(6)	[1455.01]	2.54 <	2.72	-0.18		0.1208	0.6525	0.4636	-0.2897	0.6690	0.5207	C(11)	135
H(8)	....	C(20)	[ ]	2.62<<	2.90	-0.28	Intra	0.1208	0.6525	0.4636	0.5274	0.6258	0.5251		
H(8)	....	H(9)	[ ]	2.33 <	2.40	-0.07	Intra	0.1208	0.6525	0.4636	-0.0070	0.6029	0.3832		
H(8)	....	H(18)	[3466.01]	2.28 <	2.40	-0.12		0.1208	0.6525	0.4636	0.0379	0.7515	0.4140	C(11)	122
H(9)	....	H(8)	[ ]	2.33 <	2.40	-0.07	Intra	-0.0070	0.6029	0.3832	0.1208	0.6525	0.4636		
H(9)	....	H(10)	[ ]	2.33 <	2.40	-0.07	Intra	-0.0070	0.6029	0.3832	0.2289	0.5068	0.3480		
H(9)	....	S(2)	[3466.01]	3.19	3.00	0.19		-0.0070	0.6029	0.3832	-0.3899	0.7391	0.3800	C(12)	142
H(10)	....	O(1)	[ ]	2.52<<	2.72	-0.20	Intra	0.2289	0.5068	0.3480	0.5950	0.4146	0.3417		
H(10)	....	C(1)	[ ]	2.63<<	2.90	-0.27	Intra	0.2289	0.5068	0.3480	0.6300	0.4329	0.3870		
H(10)	....	H(9)	[ ]	2.33 <	2.40	-0.07	Intra	0.2289	0.5068	0.3480	-0.0070	0.6029	0.3832		
H(11)	....	S(1)	[ ]	3.03	3.00	0.03	Intra	1.1285	0.4370	0.6548	0.7261	0.4551	0.7425		
H(11)	....	O(3)	[ ]	2.48<<	2.72	-0.24	Intra	1.1285	0.4370	0.6548	1.3628	0.4027	0.5726		
H(11)	....	O(5)	[ ]	2.36<<	2.72	-0.36	Intra	1.1285	0.4370	0.6548	1.2260	0.3381	0.7092		
H(11)	....	O(7)	[1655.01]	2.48<<	2.72	-0.24		1.1285	0.4370	0.6548	1.1614	0.5693	0.6594	C(16)	130

==

H(11)	....	C(15)	[ ]	2.55<<	2.90	-0.35	Intra	1.1285	0.4370	0.6548	1.1287	0.3946	0.5597		
H(11)	....	C(17)	[1655.01]	3.02	2.90	0.12		1.1285	0.4370	0.6548	1.7230	0.4439	0.6719	C(16)	147
H(11)	....	H(12)	[1655.01]	2.22 <	2.40	-0.18		1.1285	0.4370	0.6548	1.5621	0.4163	0.6619	C(16)	134
H(11)	....	H(13)	[ ]	2.34 <	2.40	-0.06	Intra	1.1285	0.4370	0.6548	0.7093	0.4916	0.6552		
H(12)	....	O(3)	[1455.01]	2.49<<	2.72	-0.23		0.5621	0.4163	0.6619	0.3628	0.4027	0.5726	C(17)	128
H(12)	....	O(4)	[ ]	2.91	2.72	0.19	Intra	0.5621	0.4163	0.6619	0.8638	0.2846	0.6757		
H(12)	....	O(5)	[1455.01]	2.53 <	2.72	-0.19		0.5621	0.4163	0.6619	0.2260	0.3381	0.7092	C(17)	136
H(12)	....	N(1)	[ ]	2.56 <	2.75	-0.19	Intra	0.5621	0.4163	0.6619	0.9272	0.3918	0.5941		
H(12)	....	C(16)	[1455.01]	3.00	2.90	0.10		0.5621	0.4163	0.6619	-0.0319	0.4058	0.6501	C(17)	150
H(12)	....	C(18)	[ ]	2.75 <	2.90	-0.15	Intra	0.5621	0.4163	0.6619	1.0102	0.3353	0.6792		
H(12)	....	H(3)	[ ]	2.35 <	2.40	-0.05	Intra	0.5621	0.4163	0.6619	0.7661	0.3812	0.5828		
H(12)	....	H(11)	[1455.01]	2.22 <	2.40	-0.18		0.5621	0.4163	0.6619	0.1285	0.4370	0.6548	C(17)	137
H(13)	....	O(7)	[1655.01]	2.69 <	2.72	-0.03		0.7093	0.4916	0.6552	1.1614	0.5693	0.6594	C(17)	114
H(13)	....	N(1)	[ ]	2.66 <	2.75	-0.09	Intra	0.7093	0.4916	0.6552	0.9272	0.3918	0.5941		
H(13)	....	C(23)	[ ]	3.02	2.90	0.12	Intra	0.7093	0.4916	0.6552	0.3275	0.6158	0.6640	C(17)	135
H(13)	....	H(1)	[ ]	2.49	2.40	0.09	Intra	0.7093	0.4916	0.6552	0.8819	0.4911	0.7471		
H(13)	....	H(11)	[ ]	2.34 <	2.40	-0.06	Intra	0.7093	0.4916	0.6552	1.1285	0.4370	0.6548		
H(14)	....	O(4)	[ ]	2.79	2.72	0.07	Intra	1.1457	0.2737	0.7698	0.8638	0.2846	0.6757		
H(14)	....	C(18)	[ ]	2.66<<	2.90	-0.24	Intra	1.1457	0.2737	0.7698	1.0102	0.3353	0.6792		
H(14)	....	S(2)	[4646.01]	3.09	3.00	0.09		1.1457	0.2737	0.7698	0.8899	0.2609	0.8800	C(19)	163
H(15)	....	O(4)	[1655.01]	2.89	2.72	0.17		1.4564	0.2784	0.7559	1.8638	0.2846	0.6757	C(19)	113
H(15)	....	O(8)	[4746.01]	2.73	2.72	0.01		1.4564	0.2784	0.7559	1.6030	0.1461	0.7910	C(19)	109
H(15)	....	H(19)	[4746.01]	2.48	2.40	0.08		1.4564	0.2784	0.7559	1.4520	0.2496	0.8512	C(19)	111
H(16)	....	O(4)	[ ]	2.48<<	2.72	-0.24	Intra	1.2600	0.2325	0.7195	0.8638	0.2846	0.6757		
H(16)	....	C(18)	[ ]	2.51<<	2.90	-0.39	Intra	1.2600	0.2325	0.7195	1.0102	0.3353	0.6792		
H(16)	....	H(5)	[3556.01]	2.49	2.40	0.09		1.2600	0.2325	0.7195	1.4134	0.1817	0.6341	C(19)	141
H(17)	....	S(2)	[1655.01]	3.19	3.00	0.19		0.6737	0.6368	0.6237	1.1101	0.7609	0.6200	C(21)	120
H(17)	....	O(6)	[ ]	2.69 <	2.72	-0.03	Intra	0.6737	0.6368	0.6237	0.7103	0.6690	0.5207		
H(17)	....	O(7)	[1655.01]	2.90	2.72	0.18		0.6737	0.6368	0.6237	1.1614	0.5693	0.6594	C(21)	162
H(17)	....	O(8)	[ ]	2.58 <	2.72	-0.14	Intra	0.6737	0.6368	0.6237	0.3970	0.6461	0.7090		
H(17)	....	C(20)	[ ]	2.62<<	2.90	-0.28	Intra	0.6737	0.6368	0.6237	0.5274	0.6258	0.5251		
H(17)	....	H(18)	[ ]	2.40	2.40	0.00	Intra	0.6737	0.6368	0.6237	0.5379	0.7485	0.5860		
H(17)	....	H(19)	[ ]	2.29 <	2.40	-0.11	Intra	0.6737	0.6368	0.6237	0.5480	0.7496	0.6488		
H(18)	....	S(2)	[1655.01]	3.01	3.00	0.01		0.5379	0.7485	0.5860	1.1101	0.7609	0.6200	C(22)	102
H(18)	....	O(6)	[ ]	2.39<<	2.72	-0.33	Intra	0.5379	0.7485	0.5860	0.7103	0.6690	0.5207	C(22)	121
H(18)	....	N(2)	[ ]	2.63 <	2.75	-0.12	Intra	0.5379	0.7485	0.5860	0.3927	0.6148	0.5702		
H(18)	....	C(20)	[ ]	2.77 <	2.90	-0.13	Intra	0.5379	0.7485	0.5860	0.5274	0.6258	0.5251		
H(18)	....	H(2)	[ ]	2.52	2.40	0.12	Intra	0.5379	0.7485	0.5860	0.0355	0.7408	0.5827		
H(18)	....	H(2)	[1655.01]	2.50	2.40	0.10		0.5379	0.7485	0.5860	1.0355	0.7408	0.5827	C(22)	116
H(18)	....	H(17)	[ ]	2.40	2.40	0.00	Intra	0.5379	0.7485	0.5860	0.6737	0.6368	0.6237		
H(18)	....	C(11)	[3566.01]	2.89 <	2.90	-0.01		0.5379	0.7485	0.5860	0.7157	0.8873	0.5502	C(22)	136
H(18)	....	H(8)	[3566.01]	2.28 <	2.40	-0.12		0.5379	0.7485	0.5860	0.6208	0.8475	0.5364	C(22)	146
H(19)	....	S(2)	[1655.01]	2.92 <	3.00	-0.08		0.5480	0.7496	0.6488	1.1101	0.7609	0.6200	C(22)	108
H(19)	....	O(8)	[ ]	2.58 <	2.72	-0.14	Intra	0.5480	0.7496	0.6488	0.3970	0.6461	0.7090		
H(19)	....	C(23)	[ ]	2.76 <	2.90	-0.14	Intra	0.5480	0.7496	0.6488	0.3275	0.6158	0.6640		
H(19)	....	H(17)	[ ]	2.29 <	2.40	-0.11	Intra	0.5480	0.7496	0.6488	0.6737	0.6368	0.6237		
H(19)	....	C(19)	[4756.01]	2.98	2.90	0.08		0.5480	0.7496	0.6488	0.7240	0.7756	0.7588	C(22)	161
H(19)	....	H(15)	[4756.01]	2.48	2.40	0.08		0.5480	0.7496	0.6488	0.5436	0.7784	0.7441	C(22)	148

H(20)	....	S(1)	[ ]	3.11	3.00	0.11	Intra	0.3137	0.5752	0.7657	0.7261	0.4551	0.7425	C(24)	145
H(20)	....	O(7)	[ ]	2.81	2.72	0.09	Intra	0.3137	0.5752	0.7657	0.1614	0.5693	0.6594		
H(20)	....	C(23)	[ ]	2.70<<	2.90	-0.20	Intra	0.3137	0.5752	0.7657	0.3275	0.6158	0.6640		
H(20)	....	O(1)	[2565.01]	2.82	2.72	0.10		0.3137	0.5752	0.7657	-0.0950	0.5854	0.8417		
H(21)	....	O(7)	[ ]	2.46<<	2.72	-0.26	Intra	0.0597	0.6200	0.7465	0.1614	0.5693	0.6594		
H(21)	....	C(23)	[ ]	2.49<<	2.90	-0.41	Intra	0.0597	0.6200	0.7465	0.3275	0.6158	0.6640		
H(21)	....	H(1)	[1455.01]	2.57	2.40	0.17		0.0597	0.6200	0.7465	-0.1181	0.4911	0.7471	C(24)	113
H(21)	....	O(1)	[2565.01]	2.62 <	2.72	-0.10		0.0597	0.6200	0.7465	-0.0950	0.5854	0.8417		
H(22)	....	O(1)	[2565.01]	2.73	2.72	0.01		0.2778	0.6567	0.7837	-0.0950	0.5854	0.8417		
H(22)	....	O(4)	[4656.01]	2.70 <	2.72	-0.02		0.2778	0.6567	0.7837	0.1362	0.7846	0.8243	C(24)	146

Summary of Shortest Inter Contacts with d(I-J) < R(I) + R(J) + 0.2 of Residue # 1 to Neighbouring ARU'S

Nr	ARU	Nr.Cont.	d(min)	Del	XHn	X	- At(I)		At(J)	- Y	YHn	Note	Partaking ARU's in Close Contact Resd.
1	[1455.01]	36	2.0800	-0.64	1	N(1)	- H(3)	...	O(3)	-C(15)	0	<<	1455.01
2	[3466.01]	4	2.2800	-0.12	1	C(11)	- H(8)	...	H(18)	-C(22)	2	<	3466.01
3	[3566.01]	4	2.2800	-0.12	2	C(22)	- H(18)	...	H(8)	-C(11)	1	<	3566.01
4	[4656.01]	2	2.7000	-0.02	3	C(24)	- H(22)	...	O(4)	-C(18)	0	<	4656.01
5	[2564.01]	4	2.6200	-0.10	0	C(1)	- O(1)	...	H(21)	-C(24)	3	<	2564.01
6	[1655.01]	36	2.0800	-0.64	0	C(15)	- O(3)	...	H(3)	-N(1)	1	<<	1655.01
7	[3456.01]	6	2.4900	0.09	1	C(3)	- H(5)	...	H(16)	-C(19)	3		3456.01
8	[4646.01]	2	2.7000	-0.02	0	C(18)	- O(4)	...	H(22)	-C(24)	3	<	4646.01
9	[4756.01]	4	2.4800	0.08	2	C(22)	- H(19)	...	H(15)	-C(19)	3		4756.01
10	[3556.01]	6	2.4900	0.09	3	C(19)	- H(16)	...	H(5)	-C(3)	1		3556.01
11	[4746.01]	4	2.4800	0.08	3	C(19)	- H(15)	...	H(19)	-C(22)	2		4746.01
12	[2565.01]	4	2.6200	-0.10	3	C(24)	- H(21)	...	O(1)	-C(1)	0	<	2565.01

Symbols :: < denotes contacts less than the sum of the van der Waals Radii and << contacts less than this sum minus 0.2 Angstrom.
Nr.Cont. = Number of short contacts from current ARU to surrounding ARU's (from list above).

Asymmetric Residue Unit (= ARU) Code List

ARU-CODE		CIF-CODE	Symmetry-Code		sym	TX	TY	TZ	Ires		x(cen)	y(cen)	z(cen)
[1455.01]	=	[1_455]	=-1+x,y,z	= [	1	-1	0	0	1	]	-0.320	0.491	0.573
[3466.01]	=	[2_466]	=-1/2+x,3/2-y,1-z	= [	3	-1	1	1	1	]	0.180	1.009	0.427
[3566.01]	=	[2_566]	=1/2+x,3/2-y,1-z	= [	3	0	1	1	1	]	1.180	1.009	0.427
[4656.01]	=	[3_656]	=1-x,1/2+y,3/2-z	= [	4	1	0	1	1	]	0.320	0.991	0.927
[2564.01]	=	[4_564]	=1/2-x,1-y,-1/2+z	= [	2	0	1	-1	1	]	-0.180	0.509	0.073
[1655.01]	=	[1_655]	=1+x,y,z	= [	1	1	0	0	1	]	1.680	0.491	0.573
[3456.01]	=	[2_456]	=-1/2+x,1/2-y,1-z	= [	3	-1	0	1	1	]	0.180	0.009	0.427

===

[4646.01] = [3_646] =1-x,-1/2+y,3/2-z	= [4 1 -1 1 1]	0.320	-0.009	0.927
[4756.01] = [3_756] =2-x,1/2+y,3/2-z	= [4 2 0 1 1]	1.320	0.991	0.927
[3556.01] = [2_556] =1/2+x,1/2-y,1-z	= [3 0 0 1 1]	1.180	0.009	0.427
[4746.01] = [3_746] =2-x,-1/2+y,3/2-z	= [4 2 -1 1 1]	1.320	-0.009	0.927
[2565.01] = [4_565] =1/2-x,1-y,1/2+z	= [2 0 1 0 1]	-0.180	0.509	1.073

Note: Symmetry Operations Refer to the Coordinates listed in the Fractional Coordinate Table given above

X(J) = X(sym) + TX , Y(J) = Y(sym) + TY , Z(J) = Z(sym) + TZ,

SYM - Number of the Symmetry Operator.
Ires - Residue Number.
TX, TY, TZ - Unit Cell Translations.

```
=========================================================================================================================================
Analysis of Short Non-Hydrogen Inter-Molecular Contacts For Inter-Molecular Clusters and/or Networks   (Minor Disorder Excluded)
=========================================================================================================================================

Contact-Nr Atom I[   ARU   ]      Atom J[   ARU   ]    d(I-J)   Del
-----------------------------------------------------------------------------------------------------------------------------------------
   1       O(1)  [  1555.01] ...  C(24)  [  2564.01] =  2.889  -0.33
   2       C(24) [  1555.01] ...  O(1)   [  2565.01] =  2.889  -0.33

                                                        ==============
******************************************************   Cluster  =  1 ***********************************************************
                                                        ==============

(N:M)  :  ARU   --    Connected  with (N) Interactions to/from (M) ARU(S).    T = Translated Molecule (Infinite chain etc.)
-----------------------------------------------------------------------------------------------------------------------------------------
 2  2  1555.01  --    2564.01   2565.01T
 2  2  2564.01  --    1554.01T  1555.01

                                                        ==============
******************************************************   Cluster  =  2 ***********************************************************
                                                        ==============

(N:M)  :  ARU   --    Connected  with (N) Interactions to/from (M) ARU(S).    T = Translated Molecule (Infinite chain etc.)
-----------------------------------------------------------------------------------------------------------------------------------------
 2  2  3555.01  --    4645.01   4644.01T
 2  2  4645.01  --    3556.01T  3555.01
```

Analysis of Potential Hydrogen Bonds and Schemes with d(D...A) < R(D)+R(A)+0.50, d(H...A) < R(H)+R(A)-0.12 Ang., D-H...A > 100.0 Deg

Note: - ARU codes in [] are with reference to the Coordinates printed above (Possibly transformed, when MOVE .NE. 1.555)

Nr	Typ	Res	Donor --- H....Acceptor	[ARU]	D - H	H...A	D...A	D - H...A	A..H..A*	A'..H..A"	Sum(XY,YZ)	Sum(XZ)
		1	S(1) --H(1) .. ?		1.04							
		1	S(2) --H(2) .. ?		1.09							
1		1	N(1) --H(3) ..O(3)	[1455.01]	0.88	2.08	2.890(2)	153				
2		1	N(2) --H(4) ..O(2)	[1455.01]	0.88	2.55	3.373(2)	156				
3	Intra	1	N(2) --H(4) ..O(7)	[ ]	0.88	2.34	2.687(2)	104'	100'		360	
4		1	C(4) --H(6) ..O(4)	[3556.01]	0.95	2.56	3.306(3)	136				
5		1	C(11) --H(8) ..O(6)	[1455.01]	0.95	2.54	3.284(2)	135				
6		1	C(16) --H(11) ..O(7)	[1655.01]	1.00	2.48	3.214(2)	130				
7		1	C(17) --H(12) ..O(3)	[1455.01]	0.99	2.49	3.198(2)	128				
8		1	C(17) --H(12) ..O(5)	[1455.01]	0.99	2.53	3.319(2)	136'	96'		360	
9	Intra	1	C(22) --H(18) ..O(6)	[ ]	0.99	2.39	3.018(2)	121				

Translation of ARU-Code to CIF and Equivalent Position Code

[1455.] = [1_455] =-1+x,y,z
[1655.] = [1_655] =1+x,y,z
[3556.] = [2_556] =1/2+x,1/2-y,1-z

For C--H...Acceptor Interactions See: Th. Steiner, Cryst. Rev, (1996), 6, 1-57

H-Bond classification [G.A.Jeffrey, H.Maluszynska & J.Mitra., Int.J.Biol.Macromol.(1985),7,336-348]

2-Centre (linear) D-H...X most prob. angle 160 deg - also: G.A.Jeffrey & W.Saenger, Hydrogen Bonding in Biological Structures
3-Centre (bifurcated) SUM of 3 angl. about H = 360 deg Springer-Verlag, Berlin, 1991, pp 20.
4-Centre (trifurcated)

Analysis of Potential Donor/Acceptor Atoms -- (Major Disorder Form Only)

At.Nr	D/A	#Cov.Bonds	# H	#D-H..A	#A..H	#A..H-C	Sum(A-H)	Sum(A-X)
1	S(1)	2	1 H	0	0	0	0	1
2	S(2)	2	1 H	0	0	0	0	1
3	O(1)	1	-	0	0	0	0	1
4	O(2)	1	-	0	1	0	1	2
5	O(3)	1	-	0	1	1	2	3
6	O(4)	1	-	0	0	1	1	2
7	O(5)	2	-	0	0	1	1	3

==

8	O(6)	1	-	0	0	2	2	3
9	O(7)	1	-	0	1	1	2	3
10	O(8)	2	-	0	0	0	0	2
11	N(1)	3	1 H	1	0	0	1	3
12	N(2)	3	1 H	2	0	0	2	4

==

Analysis of Hydrogen Bonded Molecular Aggregates (See also Acta Cryst. B36, 1980, 2113 - 2115) -- (Major Disorder Component Only)

==

Coordinates of Donor and Acceptor Atoms | Coordinates of D/A-Bonded Atom(s)

D/A I	[ARU]	X	Y	Z	--	D/A J	[ARU]	X	Y	Z	Atom K	X	Y	Z	I..J--K Angle
N(1)	[1555.01],	0.9272	0.3918	0.5941	>>	O(3)	[1455.01],	0.3628	0.4027	0.5726	C(15)	0.1287	0.3946	0.5597	167.90(14)
O(3)	[1555.01],	1.3628	0.4027	0.5726	<<	N(1)	[1655.01],	1.9272	0.3918	0.5941	C(15)	2.1287	0.3946	0.5597	127.73(11)
											C(16)	1.9681	0.4058	0.6501	108.01(10)
											H(3)	1.7661	0.3812	0.5828	19.10(12)
N(2)	[1555.01],	0.3927	0.6148	0.5702	>>	O(2)	[1455.01],	-0.1347	0.5171	0.5299	C(8)	-0.2264	0.4884	0.4907	141.27(12)
O(2)	[1555.01],	0.8653	0.5171	0.5299	<<	N(2)	[1655.01],	1.3927	0.6148	0.5702	C(20)	1.5274	0.6258	0.5251	102.58(10)
											C(21)	1.4806	0.6483	0.6187	136.85(10)
											H(4)	1.2507	0.5871	0.5702	17.84(11)
N(2)	[1555.01],	0.3927	0.6148	0.5702	>>	O(7)	[1555.01],	0.1614	0.5693	0.6594	C(23)	0.3275	0.6158	0.6640	63.77(10)
O(7)	[1555.01],	0.1614	0.5693	0.6594	<<	N(2)	[1555.01],	0.3927	0.6148	0.5702	C(20)	0.5274	0.6258	0.5251	169.79(13)
											C(21)	0.4806	0.6483	0.6187	63.25(8)
											H(4)	0.2507	0.5871	0.5702	57.53(12)

** Aggregate = 1 **

(N:M) : ARU -- Connected with N Hydrogen Bonds to/from M ARU(S). T = Translated Molecule (Infinite chain etc.)

4 2 1555.01 -- 1455.01T 1655.01T

** Aggregate = 2 **

(N:M) : ARU -- Connected with N Hydrogen Bonds to/from M ARU(S). T = Translated Molecule (Infinite chain etc.)

4 2 2555.01 -- 2655.01T 2455.01T

** Aggregate = 3 **

(N:M) : ARU -- Connected with N Hydrogen Bonds to/from M ARU(S). T = Translated Molecule (Infinite chain etc.)

4 2 3555.01 -- 3455.01T 3655.01T

===

==============
** Aggregate = 4 **
==============

```
(N:M)  :  ARU   --    Connected with N Hydrogen Bonds to/from M ARU(S).    T = Translated Molecule (Infinite chain etc.)
---------------------------------------------------------------------------------------------------------------------
 4  2  4555.01  --   4655.01T  4455.01T
```

==

Search for Infinite ARU-Chains (Max = 4)

==

1-Membered Infinite ARU-Chain (Translation [-1 0 0])

1555.01 1455.01

Analysis of the (Cooperative) Hydrogen Bond Network (i.e. (In)Finite O-H...O-H...O-H.. Chains and/or Rings (Max = 18 Membered))

NetworK = 1

Code Acc	Donor Atom		X	Y	Z	Acceptor Atom		X	Y	Z up to BondCode(s) of Forward Link(s)
1.555	N(1)	[1555.01]	0.9272	0.3918	0.5941	O(3)	[1455.01]	0.3628	0.4027	0.5726
	H(3)		0.7661	0.3812	0.5828					

NetworK = 2

Code Acc	Donor Atom		X	Y	Z	Acceptor Atom		X	Y	Z up to BondCode(s) of Forward Link(s)
2.555	N(2)	[1555.01]	0.3927	0.6148	0.5702	O(2)	[1455.01]	-0.1347	0.5171	0.5299
	H(4)		0.2507	0.5871	0.5702					
3.555	N(2)	[1555.01]	0.3927	0.6148	0.5702	O(7)	[1555.01]	0.1614	0.5693	0.6594
	H(4)		0.2507	0.5871	0.5702					

Hydrogen Bonds are Coded as N.IJK Where N = Sequence Number of Hydrogen Bond (NOTE: New Hbond Numbering system)
I - 5 = Nr of Translation Units Along A-Axis
J - 5 = Nr of Translation Units Along B-Axis
K - 5 = Nr of Translation Units Along C-Axis

Ring Closure Links are Indicated with 'R' and Infinite Chain Links With 'T'

==

Analysis of the Coordination Geometry

Distances are calculated from atom I of Unique Molecule Coordinate List to atom J in Asymmetric Residue Unit: ARU.

Phi = Azimuth angle(counter clockwise from X0 in X0,Y0-Plane), Mu = Angle between D and X0,Y0-plane.

'To-Code' : '--' = Bonded atoms, '<<' = .LT. sum vdW-radii - 0.2, '.<' = .LT. sum vdW-radii, '..' = .GT. sum vdW-radii.

>>>> NOTICE >>>> : The Symmetry Code Character Added to the Atom Label Applies to the Current Coordination Sphere Only.
>>>>>>>>>>>>>>>>> : Symmetry operations refer to the coordinates listed in the fractional coordinate table given above

The List May be Limited to the Shortest Distances.

3.6 Angstrom Coordination Sphere Around Atom I = S(1) [ARU = 1555.01] 0.72615 0.45508 0.74252 3.6414 8.5042 18.8871

Nr	d(I,J)	To	Atom J	Symm_Oper. on Atom J	ARU(J)	Type	Phi	Mu	X	Y	Z	XO	YO	ZO
1	1.04	--	H(1)			Intra	40.75	6.39	0.88193	0.49110	0.74706	4.4226	9.1773	19.0026
2	1.8098(19)	--	C(17)			Intra	-94.32	-83.35	0.72300	0.44389	0.67185	3.6256	8.2951	17.0895
3	2.8005(19)	<<	C(16)			Intra	-37.19	-57.05	0.96810	0.40581	0.65013	4.8547	7.5835	16.5370
4	3.104(2)	<<	C(18)			Intra	-57.53	-31.26	1.01020	0.33530	0.67921	5.0659	6.2659	17.2767
5	3.4321(16)	..	O(5)			Intra	-41.08	-14.32	1.22600	0.33814	0.70915	6.1480	6.3189	18.0383
6	3.4332(16)	..	O(5)a	[-1+x,y,z	= 1455.01]		-138.93	-14.31	0.22600	0.33814	0.70915	1.1333	6.3189	18.0383
7	2.33	<<	H(13)			Intra	97.04	-72.78	0.70933	0.49163	0.65520	3.5571	9.1872	16.6660
8	2.33	<<	H(12)			Intra	-138.64	-61.88	0.56210	0.41633	0.66189	2.8188	7.7801	16.8362
9	3.03	..	H(11)			Intra	-9.53	-47.48	1.12845	0.43696	0.65482	5.6588	8.1656	16.6563
10	3.11	..	H(20)			Intra	132.65	10.92	0.31375	0.57521	0.76568	1.5734	10.7491	19.4762
11	3.51	..	H(21)b	[1+x,y,z	= 1655.01]		61.51	1.65	1.05972	0.61999	0.74650	5.3142	11.5859	18.9883
12	3.58	..	H(15)a	[-1+x,y,z	= 1455.01]		-112.28	5.44	0.45638	0.27836	0.75587	2.2886	5.2018	19.2267
13	3.59	..	H(10)c	[1/2-x,1-y,1/2+z	= 2565.01]		162.65	48.31	0.27114	0.49322	0.84805	1.3597	9.2169	21.5714

Angles (Degrees) At1...V...At2 with Vertex V = S(1)

C(17)	C(16)	29.81(7)	C(17)	C(18)	53.51(7)	C(17)	O(5)	71.77(7)	C(17)	O(5)a	71.00(7)
C(16)	C(18)	29.38(5)	C(16)	O(5)	42.84(5)	C(16)	O(5)a	84.25(5)	C(18)	O(5)	22.67(4)
C(18)	O(5)a	75.40(5)	O(5)	O(5)a	93.85(4)						

3.6 Angstrom Coordination Sphere Around Atom I = S(2) [ARU = 1555.01] 0.11009 0.76088 0.61998 0.5521 14.2188 15.7701

Nr	d(I,J)	To	Atom J	Symm_Oper. on Atom J	ARU(J)	Type	Phi	Mu	X	Y	Z	XO	YO	ZO
1	1.09	--	H(2)			Intra	-134.89	-60.81	0.03550	0.74079	0.58269	0.1780	13.8434	14.8216
2	1.810(2)	--	C(22)			Intra	-18.81	-1.50	0.45160	0.72966	0.61812	2.2646	13.6354	15.7228
3	2.8067(19)	<<	C(21)			Intra	-48.55	-0.68	0.48060	0.64832	0.61868	2.4101	12.1154	15.7371
4	3.1302(18)	<<	C(23)			Intra	-68.10	20.98	0.32750	0.61577	0.66404	1.6423	11.5071	16.8909
5	3.3273(16)	.<	N(2)			Intra	-62.57	-22.38	0.39270	0.61475	0.57017	1.9693	11.4880	14.5031
6	3.354(2)	.<	C(22)a	[-1+x,y,z	= 1455.01]		-169.98	-0.81	-0.54840	0.72966	0.61812	-2.7501	13.6354	15.7228
7	3.4342(14)	..	O(8)			Intra	-56.15	41.23	0.39700	0.64610	0.70896	1.9908	12.0739	18.0335
8	3.556(2)	..	C(11)b	[-1/2+x,3/2-y,1-z	= 3466.01]		129.94	-29.95	-0.28430	0.88728	0.55019	-1.4257	16.5809	13.9949
9	2.32	<<	H(19)			Intra	-5.49	18.36	0.54800	0.74958	0.64876	2.7481	14.0076	16.5022
10	2.32	<<	H(18)			Intra	-6.15	-21.84	0.53787	0.74852	0.58598	2.6973	13.9878	14.9053
11	2.92	.<	H(19)a	[-1+x,y,z	= 1455.01]		-175.72	14.52	-0.45200	0.74958	0.64876	-2.2666	14.0076	16.5022
12	3.01	..	H(18)a	[-1+x,y,z	= 1455.01]		-175.40	-16.72	-0.46213	0.74852	0.58598	-2.3174	13.9878	14.9053
13	3.09	..	H(14)d	[1-x,1/2+y,3/2-z	= 4656.01]		169.42	65.04	-0.14569	0.77370	0.73017	-0.7306	14.4584	18.5730
14	3.19	..	H(9)c	[1/2+x,3/2-y,1-z	= 3566.01]		52.97	-1.45	0.49296	0.89706	0.61680	2.4721	16.7636	15.6892
15	3.19	..	H(17)a	[-1+x,y,z	= 1455.01]		-133.34	1.68	-0.32630	0.63678	0.62365	-1.6363	11.8997	15.8635
16	3.56	..	H(4)			Intra	-77.75	-20.84	0.25072	0.58708	0.57024	1.2573	10.9709	14.5049

Angles (Degrees) At1...V...At2 with Vertex V = S(2)

C(22) ,	C(21)	29.74(7)	C(22) ,	C(23)	53.17(7)	C(22) ,	N(2)	47.35(6)	C(22) ,	C(22)a	151.08(7)
C(22) ,	O(8)	54.51(7)	C(22) ,	C(11)b	136.67(7)	C(21) ,	C(23)	28.89(5)	C(21) ,	N(2)	25.64(5)
C(21) ,	C(22)a	121.42(5)	C(21) ,	O(8)	42.47(4)	C(21) ,	C(11)b	149.34(5)	C(23) ,	N(2)	43.70(4)
C(23) ,	C(22)a	101.38(5)	C(23) ,	O(8)	22.63(4)	C(23) ,	C(11)b	161.44(5)	N(2) ,	C(22)a	105.74(4)
N(2) ,	O(8)	63.89(4)	N(2) ,	C(11)b	126.30(4)	C(22)a ,	O(8)	108.25(4)	C(22)a ,	C(11)b	63.95(5)
O(8) ,	C(11)b	167.69(4)									

3.6 Angstrom Coordination Sphere Around Atom I = O(1) [ARU = 1555.01] 0.59500 0.41462 0.34165 2.9838 7.7481 8.6904

Nr	d(I,J)	To	Atom J	Symm_Oper. on Atom J	ARU(J)	Type	Phi	Mu	X	Y	Z	XO	YO	ZO
1	1.217(2)	--	C(1)			Intra	62.74	71.64	0.63000	0.43285	0.38704	3.1593	8.0888	9.8449
2	2.357(2)	<<	C(2)			Intra	-18.04	58.76	0.82680	0.39436	0.42089	4.1462	7.3695	10.7060
3	2.362(2)	<<	C(14)			Intra	110.67	47.62	0.48290	0.49434	0.41026	2.4216	9.2379	10.4356
4	2.782(3)	<<	C(3)			Intra	-39.86	32.54	0.95400	0.33419	0.40047	4.7840	6.2451	10.1866
5	2.807(3)	<<	C(13)			Intra	127.71	21.20	0.27580	0.52540	0.38155	1.3831	9.8183	9.7053
6	2.889(3)	<<	C(24)a	[1/2-x,1-y,-1/2+z	= 2564.01]		-158.12	-49.78	0.24980	0.37741	0.25493	1.2527	7.0528	6.4845
7	2.48	<<	H(5)			Intra	-48.44	14.39	0.91339	0.31825	0.36592	4.5804	5.9472	9.3077
8	2.52	<<	H(10)			Intra	136.83	3.70	0.22886	0.50678	0.34805	1.1477	9.4704	8.8532
9	2.62	.<	H(21)a	[1/2-x,1-y,-1/2+z	= 2564.01]		-140.19	-67.35	0.44028	0.38001	0.24650	2.2079	7.1014	6.2701
10	2.73	..	H(22)a	[1/2-x,1-y,-1/2+z	= 2564.01]		-144.52	-32.71	0.22224	0.34332	0.28370	1.1145	6.4157	7.2163
11	2.82	..	H(20)a	[1/2-x,1-y,-1/2+z	= 2564.01]		174.70	-43.19	0.18625	0.42479	0.26568	0.9340	7.9382	6.7580
12	2.98	..	H(1)b	[3/2-x,1-y,-1/2+z	= 2664.01]		86.24	-53.73	0.61807	0.50890	0.24706	3.0994	9.5100	6.2843
13	3.27	..	H(16)c	[-1/2+x,1/2-y,1-z	= 3456.01]		-73.25	-28.44	0.75995	0.26750	0.28052	3.8109	4.9989	7.1354
14	3.54	..	H(20)b	[3/2-x,1-y,-1/2+z	= 2664.01]		3.67	-33.04	1.18625	0.42479	0.26568	5.9487	7.9382	6.7580

Angles (Degrees) At1...V...At2 with Vertex V = O(1)

C(1)	, C(2)	33.11(11)	C(1)	, C(14)	32.50(11)	C(1)	, C(3)	63.10(12)	C(1)	, C(13)	62.13(12)
C(1)	, C(24)a	151.47(17)	C(2)	, C(14)	65.61(7)	C(2)	, C(3)	30.03(6)	C(2)	, C(13)	95.19(7)
C(2)	, C(24)a	155.46(9)	C(14)	, C(3)	95.59(8)	C(14)	, C(13)	29.78(6)	C(14)	, C(24)a	124.98(10)
C(3)	, C(13)	124.97(7)	C(3)	, C(24)a	131.94(8)	C(13)	, C(24)a	96.43(8)			

3.6 Angstrom Coordination Sphere Around Atom I = O(2) [ARU = 1555.01] 0.86530 0.51705 0.52992 4.3392 9.6623 13.4793

Nr	d(I,J)	To	Atom J	Symm_Oper. on Atom J	ARU(J)	Type	Phi	Mu	X	Y	Z	XO	YO	ZO
1	1.221(2)	--	C(8)			Intra	-130.69	-54.72	0.77360	0.48843	0.49073	3.8794	9.1274	12.4825
2	2.366(2)	<<	C(7)			Intra	-87.42	-38.51	0.88190	0.41809	0.47201	4.4225	7.8130	12.0063
3	2.373(2)	<<	C(9)			Intra	176.07	-49.37	0.55790	0.52272	0.45912	2.7977	9.7682	11.6784
4	2.648(2)	<<	C(20)			Intra	129.83	-2.64	0.52740	0.62575	0.52513	2.6448	11.6936	13.3575
5	2.749(2)	<<	C(15)			Intra	-60.01	15.97	1.12870	0.39458	0.55965	5.6601	7.3736	14.2355
6	2.844(2)	<<	C(6)			Intra	-69.23	-13.80	1.06060	0.37888	0.50325	5.3186	7.0802	12.8009
7	2.845(2)	<<	C(10)			Intra	150.02	-27.77	0.43040	0.58437	0.47781	2.1583	10.9203	12.1538
8	2.8719(19)	.<	N(1)			Intra	-82.45	34.67	0.92720	0.39175	0.59414	4.6496	7.3207	15.1128
9	2.9539(18)	.<	O(6)			Intra	105.31	-4.56	0.71030	0.66903	0.52069	3.5619	12.5024	13.2445
10	3.162(2)	..	N(2)			Intra	142.39	18.89	0.39270	0.61475	0.57017	1.9693	11.4880	14.5031
11	3.231(2)	..	C(11)b	[1+x,y,z	= 1655.01]		45.50	-39.11	1.21570	0.61272	0.44981	6.0964	11.4501	11.4416
12	3.372(2)	..	C(10)b	[1+x,y,z	= 1655.01]		23.94	-23.15	1.43040	0.58437	0.47781	7.1730	10.9203	12.1538
13	3.373(2)	..	N(2)b	[1+x,y,z	= 1655.01]		34.62	17.67	1.39270	0.61475	0.57017	6.9840	11.4880	14.5031
14	3.460(2)	..	O(3)			Intra	-40.58	18.29	1.36280	0.40269	0.57261	6.8340	7.5252	14.5652
15	3.478(2)	..	O(3)a	[-1+x,y,z	= 1455.01]		-139.70	18.19	0.36280	0.40269	0.57261	1.8193	7.5252	14.5652
16	2.55	.<	H(4)b	[1+x,y,z	= 1655.01]		34.10	23.72	1.25072	0.58708	0.57024	6.2720	10.9709	14.5049
17	2.92	..	H(3)			Intra	-101.08	27.48	0.76613	0.38116	0.58283	3.8419	7.1229	14.8252
18	3.30	..	H(8)b	[1+x,y,z	= 1655.01]		63.15	-30.74	1.12084	0.65253	0.46358	5.6207	12.1940	11.7919
19	3.32	..	H(13)			Intra	-148.73	73.98	0.70933	0.49163	0.65520	3.5571	9.1872	16.6660
20	3.41	..	H(17)			Intra	113.24	44.40	0.67370	0.63678	0.62365	3.3784	11.8997	15.8635
21	3.50	..	H(4)			Intra	156.99	17.03	0.25072	0.58708	0.57024	1.2573	10.9709	14.5049

Angles (Degrees) At1...V...At2 with Vertex V = O(2)

C(8)	C(7)	33.13(10)	C(8)	C(9)	32.37(10)	C(8)	C(20)	93.29(12)	C(8)	C(15)	92.34(11)
C(8)	C(6)	62.44(10)	C(8)	C(10)	61.62(11)	C(8)	N(1)	98.51(10)	C(8)	O(6)	104.90(11)
C(8)	N(2)	103.59(12)	C(8)	C(11)b	86.12(11)	C(8)	C(10)b	99.15(12)	C(8)	N(2)b	141.27(12)
C(8)	O(3)	104.91(11)	C(8)	O(3)a	73.32(11)	C(7)	C(9)	65.49(7)	C(7)	C(20)	126.41(8)
C(7)	C(15)	60.23(6)	C(7)	C(6)	29.49(6)	C(7)	C(10)	94.73(7)	C(7)	N(1)	73.32(6)
C(7)	O(6)	135.35(7)	C(7)	N(2)	132.79(8)	C(7)	C(11)b	91.19(7)	C(7)	C(10)b	90.99(7)
C(7)	N(2)b	125.77(8)	C(7)	O(3)	71.77(6)	C(7)	O(3)a	74.90(6)	C(9)	C(20)	61.00(7)
C(9)	C(15)	123.93(7)	C(9)	C(6)	94.77(7)	C(9)	C(10)	29.40(6)	C(9)	N(1)	122.57(7)
C(9)	O(6)	74.09(6)	C(9)	N(2)	74.52(6)	C(9)	C(11)b	81.37(6)	C(9)	C(10)b	103.35(7)
C(9)	N(2)b	135.69(7)	C(9)	O(3)	137.24(7)	C(9)	O(3)a	78.10(6)	C(20)	C(15)	163.52(8)
C(20)	C(6)	154.95(8)	C(20)	C(10)	31.67(5)	C(20)	N(1)	136.12(7)	C(20)	O(6)	24.55(5)
C(20)	N(2)	24.82(5)	C(20)	C(11)b	83.95(6)	C(20)	C(10)b	103.50(6)	C(20)	N(2)b	95.77(6)
C(20)	O(3)	161.75(7)	C(20)	O(3)a	91.27(6)	C(15)	C(6)	31.14(5)	C(15)	C(10)	149.85(7)
C(15)	N(1)	27.46(5)	C(15)	O(6)	161.61(7)	C(15)	N(2)	138.76(7)	C(15)	C(11)b	111.90(7)
C(15)	C(10)b	90.86(6)	C(15)	N(2)b	89.45(6)	C(15)	O(3)	18.70(5)	C(15)	O(3)a	75.56(6)
C(6)	C(10)	123.66(7)	C(6)	N(1)	50.07(5)	C(6)	O(6)	160.86(7)	C(6)	N(2)	149.28(7)
C(6)	C(11)b	99.48(6)	C(6)	C(10)b	87.45(6)	C(6)	N(2)b	107.09(6)	C(6)	O(3)	42.75(5)

==

C(6)	O(3)a	76.47(6)	C(10)	N(1)	135.10(7)	C(10)	O(6)	48.41(5)	C(10)	N(2)	47.24(5)
C(10)	C(11)b	83.01(6)	C(10)	C(10)b	107.22(6)	C(10)	N(2)b	120.21(6)	C(10)	O(3)	166.41(6)
C(10)	O(3)a	82.06(6)	N(1)	O(6)	149.05(6)	N(1)	N(2)	111.57(6)	N(1)	C(11)b	138.70(7)
N(1)	C(10)b	115.91(6)	N(1)	N(2)b	100.60(6)	N(1)	O(3)	40.53(4)	N(1)	O(3)a	53.11(5)
O(6)	N(2)	43.39(4)	O(6)	C(11)b	63.95(5)	O(6)	C(10)b	80.28(5)	O(6)	N(2)b	73.14(5)
O(6)	O(3)	143.96(6)	O(6)	O(3)a	115.15(6)	N(2)	C(11)b	107.00(5)	N(2)	C(10)b	122.80(5)
N(2)	N(2)b	100.19(5)	N(2)	O(3)	142.70(6)	N(2)	O(3)a	73.18(5)	C(11)b	C(10)b	24.28(5)
C(11)b	N(2)b	57.68(5)	C(11)b	O(3)	98.49(6)	C(11)b	O(3)a	158.61(6)	C(10)b	N(2)b	42.13(4)
C(10)b	O(3)	75.39(5)	C(10)b	O(3)a	163.92(6)	N(2)b	O(3)	70.95(5)	N(2)b	O(3)a	143.70(5)
O(3)	O(3)a	92.57(5)									

3.6 Angstrom Coordination Sphere Around Atom I = O(3) [ARU = 1555.01] 1.36280 0.40269 0.57261 6.8340 7.5252 14.5652

Nr	d(I,J)	To	Atom J	Symm_Oper. on Atom J	ARU(J)	Type	Phi	Mu	X	Y	Z	XO	YO	ZO
1	1.229(2)	--	C(15)			Intra	-172.64	-15.56	1.12870	0.39458	0.55965	5.6601	7.3736	14.2355
2	2.261(2)	<<	N(1)			Intra	-174.65	14.02	0.92720	0.39175	0.59414	4.6496	7.3207	15.1128
3	2.368(2)	<<	C(6)			Intra	-163.64	-48.16	1.06060	0.37888	0.50325	5.3186	7.0802	12.8009
4	2.795(2)	<<	C(16)			Intra	178.31	44.88	0.96810	0.40581	0.65013	4.8547	7.5835	16.5370
5	2.890(2)	.<	N(1)a	[1+x,y,z	= 1655.01]		-4.13	10.92	1.92720	0.39175	0.59414	9.6643	7.3207	15.1128
6	2.918(3)	<<	C(5)			Intra	-119.12	-52.62	1.19090	0.31988	0.48145	5.9720	5.9777	12.2464
7	3.198(2)	.<	C(17)a	[1+x,y,z	= 1655.01]		23.09	52.12	1.72300	0.44389	0.67185	8.6403	8.2951	17.0895
8	3.339(2)	..	C(8)a	[1+x,y,z	= 1655.01]		37.87	-38.59	1.77360	0.48843	0.49073	8.8941	9.1274	12.4825
9	3.460(2)	..	O(2)			Intra	139.42	-18.29	0.86530	0.51705	0.52992	4.3392	9.6623	13.4793
10	3.473(2)	..	C(18)			Intra	-144.54	51.32	1.01020	0.33530	0.67921	5.0659	6.2659	17.2767
11	3.478(2)	..	O(2)a	[1+x,y,z	= 1655.01]		40.30	-18.19	1.86530	0.51705	0.52992	9.3539	9.6623	13.4793
12	3.528(2)	..	C(7)			Intra	173.19	-46.50	0.88190	0.41809	0.47201	4.4225	7.8130	12.0063
13	2.08	<<	H(3)a	[1+x,y,z	= 1655.01]		-11.25	7.18	1.76613	0.38116	0.58283	8.8566	7.1229	14.8252
14	2.48	<<	H(11)			Intra	151.41	57.38	1.12845	0.43696	0.65482	5.6588	8.1656	16.6563
15	2.49	<<	H(12)a	[1+x,y,z	= 1655.01]		14.31	65.57	1.56210	0.41633	0.66189	7.8335	7.7801	16.8362
16	2.71	.<	H(7)			Intra	-96.41	-41.42	1.31750	0.29446	0.50203	6.6069	5.5027	12.7699
17	3.03	..	H(3)			Intra	-172.34	4.92	0.76613	0.38116	0.58283	3.8419	7.1229	14.8252
18	3.19	..	H(13)a	[1+x,y,z	= 1655.01]		43.72	41.14	1.70933	0.49163	0.65520	8.5718	9.1872	16.6660
19	3.49	..	H(4)a	[1+x,y,z	= 1655.01]		99.26	-0.99	1.25072	0.58708	0.57024	6.2720	10.9709	14.5049
20	3.53	..	H(6)b	[1/2+x,1/2-y,1-z	= 3556.01]		-58.30	4.58	1.73158	0.24248	0.58370	8.6834	4.5313	14.8473

Angles (Degrees) At1...V...At2 with Vertex V = O(3)

C(15) , N(1)	29.65(10)	C(15) , C(6)	33.44(11)	C(15) , C(16)	61.00(11)	C(15) , N(1)a	167.90(15)
C(15) , C(5)	55.89(11)	C(15) , C(17)a	141.36(13)	C(15) , C(8)a	118.77(13)	C(15) , O(2)	45.82(11)
C(15) , C(18)	71.24(11)	C(15) , O(2)a	133.18(13)	C(15) , C(7)	33.11(10)	N(1) , C(6)	62.95(7)
N(1) , C(16)	31.44(6)	N(1) , N(1)a	153.35(8)	N(1) , C(5)	81.90(7)	N(1) , C(17)a	112.10(8)
N(1) , C(8)a	142.23(8)	N(1) , O(2)	55.62(6)	N(1) , C(18)	44.47(6)	N(1) , O(2)a	146.21(8)
N(1) , C(7)	61.49(6)	C(6) , C(16)	94.38(8)	C(6) , N(1)a	138.99(9)	C(6) , C(5)	28.26(6)
C(6) , C(17)a	174.15(9)	C(6) , C(8)a	91.16(7)	C(6) , O(2)	54.60(6)	C(6) , C(18)	100.82(7)
C(6) , O(2)a	110.28(7)	C(6) , C(7)	15.73(5)	C(16) , N(1)a	124.15(7)	C(16) , C(5)	111.26(8)
C(16) , C(17)a	80.68(6)	C(16) , C(8)a	150.13(8)	C(16) , O(2)	72.41(6)	C(16) , C(18)	25.33(5)
C(16) , O(2)a	136.11(7)	C(16) , C(7)	91.49(6)	N(1)a , C(5)	113.73(7)	N(1)a , C(17)a	46.71(5)
N(1)a , C(8)a	63.12(5)	N(1)a , O(2)	144.03(7)	N(1)a , C(18)	108.96(6)	N(1)a , O(2)a	52.63(5)
N(1)a , C(7)	144.36(7)	C(5) , C(17)a	157.19(8)	C(5) , C(8)a	86.62(6)	C(5) , O(2)	82.25(6)
C(5) , C(18)	106.12(7)	C(5) , O(2)a	106.97(6)	C(5) , C(7)	42.69(5)	C(17)a , C(8)a	91.63(6)
C(17)a , O(2)	120.42(7)	C(17)a , C(18)	76.03(6)	C(17)a , O(2)a	71.90(5)	C(17)a , C(7)	159.88(8)
C(8)a , O(2)	87.29(6)	C(8)a , C(18)	167.16(7)	C(8)a , O(2)a	20.51(4)	C(8)a , C(7)	86.00(6)
O(2) , C(18)	95.85(5)	O(2) , O(2)a	92.57(5)	O(2) , C(7)	39.56(4)	C(18) , O(2)a	146.65(6)
C(18) , C(7)	104.35(6)	O(2)a , C(7)	102.63(6)				

3.6 Angstrom Coordination Sphere Around Atom I = O(4) [ARU = 1555.01] 0.86380 0.28464 0.67573 4.3317 5.3192 17.1882

Nr	d(I,J)	To	Atom J	Symm_Oper. on Atom J	ARU(J)	Type	Phi	Mu	X	Y	Z	XO	YO	ZO
1	1.201(3)	--	C(18)			Intra	52.21	4.23	1.01020	0.33530	0.67921	5.0659	6.2659	17.2767
2	2.241(2)	<<	O(5)			Intra	28.83	22.29	1.22600	0.33814	0.70915	6.1480	6.3189	18.0383
3	2.414(2)	<<	C(16)			Intra	76.99	-15.65	0.96810	0.40581	0.65013	4.8547	7.5835	16.5370
4	2.659(3)	<<	C(19)			Intra	-4.65	38.75	1.27600	0.27564	0.74116	6.3988	5.1510	18.8525
5	2.901(2)	.<	N(1)			Intra	80.97	-45.68	0.92720	0.39175	0.59414	4.6496	7.3207	15.1128
6	3.060(2)	.<	C(17)			Intra	103.35	-1.85	0.72300	0.44389	0.67185	3.6256	8.2951	17.0895
7	3.306(3)	..	C(4)b	[-1/2+x,1/2-y,1-z	= 3456.01]		-126.16	-54.85	0.63990	0.20242	0.56947	3.2089	3.7827	14.4853
8	3.389(3)	..	C(19)a	[-1+x,y,z	= 1455.01]		-176.73	29.41	0.27600	0.27564	0.74116	1.3841	5.1510	18.8525
9	3.457(2)	..	O(5)a	[-1+x,y,z	= 1455.01]		162.64	14.23	0.22600	0.33814	0.70915	1.1333	6.3189	18.0383
10	3.551(3)	..	C(24)d	[1-x,-1/2+y,3/2-z	= 4646.01]		-100.69	29.78	0.74980	0.12259	0.74507	3.7600	2.2909	18.9520
11	3.593(3)	..	C(3)b	[-1/2+x,1/2-y,1-z	= 3456.01]		-132.78	-32.64	0.45400	0.16581	0.59953	2.2767	3.0985	15.2499
12	2.48	<<	H(16)			Intra	-26.13	26.70	1.25995	0.23250	0.71948	6.3183	4.3448	18.3011
13	2.56	.<	H(6)b	[-1/2+x,1/2-y,1-z	= 3456.01]		-130.08	-66.26	0.73158	0.24248	0.58370	3.6687	4.5313	14.8473
14	2.70	.<	H(22)d	[1-x,-1/2+y,3/2-z	= 4646.01]		-106.53	22.48	0.72224	0.15668	0.71630	3.6218	2.9279	18.2202
15	2.79	..	H(14)			Intra	-8.23	59.17	1.14569	0.27370	0.76983	5.7453	5.1147	19.5818
16	2.89	..	H(15)a	[-1+x,y,z	= 1455.01]		-176.71	44.89	0.45638	0.27836	0.75587	2.2886	5.2018	19.2267
17	2.91	..	H(12)			Intra	121.58	-6.95	0.56210	0.41633	0.66189	2.8188	7.7801	16.8362
18	3.01	..	H(3)			Intra	105.19	-51.66	0.76613	0.38116	0.58283	3.8419	7.1229	14.8252
19	3.15	..	H(5)b	[-1/2+x,1/2-y,1-z	= 3456.01]		-139.59	-19.65	0.41339	0.18175	0.63408	2.0730	3.3964	16.1288
20	3.19	..	H(11)			Intra	65.00	-9.61	1.12845	0.43696	0.65482	5.6588	8.1656	16.6563
21	3.37	..	H(16)a	[-1+x,y,z	= 1455.01]		-162.16	19.28	0.25995	0.23250	0.71948	1.3036	4.3448	18.3011
22	3.52	..	H(5)c	[1/2+x,1/2-y,1-z	= 3556.01]		-34.90	-17.50	1.41339	0.18175	0.63408	7.0877	3.3964	16.1288

Angles (Degrees) At1...V...At2 with Vertex V = O(4)

C(18)	O(5)	28.96(11)	C(18)	C(16)	31.58(11)	C(18)	C(19)	61.88(13)	C(18)	N(1)	56.08(11)
C(18)	C(17)	51.46(12)	C(18)	C(4)b	129.36(13)	C(18)	C(19)a	122.31(14)	C(18)	O(5)a	108.63(13)
C(18)	C(24)d	137.21(14)	C(18)	C(3)b	151.20(13)	O(5)	C(16)	60.53(7)	O(5)	C(19)	32.93(8)
O(5)	N(1)	82.80(7)	O(5)	C(17)	76.43(7)	O(5)	C(4)b	142.46(9)	O(5)	C(19)a	122.74(8)
O(5)	O(5)a	121.84(7)	O(5)	C(24)d	108.82(8)	O(5)	C(3)b	160.73(9)	C(16)	C(19)	93.43(9)
C(16)	N(1)	30.21(5)	C(16)	C(17)	29.41(6)	C(16)	C(4)b	106.80(7)	C(16)	C(19)a	111.56(9)
C(16)	O(5)a	89.74(7)	C(16)	C(24)d	165.71(8)	C(16)	C(3)b	123.93(8)	C(19)	N(1)	113.97(9)
C(19)	C(17)	105.13(8)	C(19)	C(4)b	138.28(9)	C(19)	C(19)a	111.44(8)	C(19)	O(5)a	125.70(8)
C(19)	C(24)d	76.13(8)	C(19)	C(3)b	138.00(8)	N(1)	C(17)	48.02(5)	N(1)	C(4)b	76.88(6)
N(1)	C(19)a	118.74(8)	N(1)	O(5)a	94.46(6)	N(1)	C(24)d	164.05(7)	N(1)	C(3)b	95.92(6)
C(17)	C(4)b	110.32(7)	C(17)	C(19)a	82.15(7)	C(17)	O(5)a	60.87(5)	C(17)	C(24)d	143.93(8)
C(17)	C(3)b	116.85(7)	C(4)b	C(19)a	94.76(8)	C(4)b	O(5)a	91.22(7)	C(4)b	C(24)d	87.42(6)
C(4)b	C(3)b	22.69(5)	C(19)a	O(5)a	24.35(5)	C(19)a	C(24)d	64.77(7)	C(19)a	C(3)b	74.74(7)
O(5)a	C(24)d	88.60(7)	O(5)a	C(3)b	77.42(6)	C(24)d	C(3)b	69.44(6)			

3.6 Angstrom Coordination Sphere Around Atom I = O(5) [ARU = 1555.01] 1.22600 0.33814 0.70915 6.1480 6.3189 18.0383

Nr	d(I,J)	To	Atom J	Symm_Oper. on Atom J	ARU(J)	Type	Phi	Mu	X	Y	Z	XO	YO	ZO
1	1.324(2)	--	C(18)			Intra	-177.19	-35.10	1.01020	0.33530	0.67921	5.0659	6.2659	17.2767
2	1.446(3)	--	C(19)			Intra	-77.88	34.28	1.27600	0.27564	0.74116	6.3988	5.1510	18.8525
3	2.241(2)	<<	O(4)			Intra	-151.17	-22.29	0.86380	0.28464	0.67573	4.3317	5.3192	17.1882
4	2.351(2)	<<	C(16)			Intra	135.64	-39.69	0.96810	0.40581	0.65013	4.8547	7.5835	16.5370
5	3.319(2)	..	C(17)a	[1+x,y,z	= 1655.01]		38.41	-16.61	1.72300	0.44389	0.67185	8.6403	8.2951	17.0895
6	3.342(2)	..	C(17)			Intra	141.92	-16.49	0.72300	0.44389	0.67185	3.6256	8.2951	17.0895
7	3.4321(16)	..	S(1)			Intra	138.92	14.32	0.72615	0.45508	0.74252	3.6414	8.5042	18.8871
8	3.4332(16)	..	S(1)a	[1+x,y,z	= 1655.01]		41.07	14.31	1.72615	0.45508	0.74252	8.6561	8.5042	18.8871
9	3.436(2)	..	N(1)			Intra	146.23	-58.36	0.92720	0.39175	0.59414	4.6496	7.3207	15.1128
10	3.457(2)	..	O(4)a	[1+x,y,z	= 1655.01]		-17.36	-14.23	1.86380	0.28464	0.67573	9.3464	5.3192	17.1882
11	2.00	<<	H(14)			Intra	-108.49	50.56	1.14569	0.27370	0.76983	5.7453	5.1147	19.5818
12	2.00	<<	H(15)			Intra	-44.04	36.48	1.45638	0.27836	0.75587	7.3033	5.2018	19.2267
13	2.00	<<	H(16)			Intra	-85.07	7.55	1.25995	0.23250	0.71948	6.3183	4.3448	18.3011
14	2.36	<<	H(11)			Intra	104.84	-35.88	1.12845	0.43696	0.65482	5.6588	8.1656	16.6563
15	2.53	.<	H(12)a	[1+x,y,z	= 1655.01]		40.92	-28.32	1.56210	0.41633	0.66189	7.8335	7.7801	16.8362
16	3.48	..	H(1)			Intra	121.12	16.11	0.88193	0.49110	0.74706	4.4226	9.1773	19.0026

Angles (Degrees) At1...V...At2 with Vertex V = O(5)

C(18) , C(19)	115.67(17)	C(18) , O(4)	26.05(9)	C(18) , C(16)	37.32(10)	C(18) , C(17)a	118.23(12)
C(18) , C(17)	40.86(10)	C(18) , S(1)	64.59(10)	C(18) , S(1)a	139.88(12)	C(18) , N(1)	33.47(10)
C(18) , O(4)a	127.08(12)	C(19) , O(4)	89.64(13)	C(19) , C(16)	152.84(15)	C(19) , C(17)a	120.78(13)
C(19) , C(17)	140.23(13)	C(19) , S(1)	120.12(13)	C(19) , S(1)a	104.38(12)	C(19) , N(1)	142.25(13)
C(19) , O(4)a	75.19(12)	O(4) , C(16)	63.37(7)	O(4) , C(17)a	139.98(8)	O(4) , C(17)	62.89(6)
O(4) , S(1)	77.64(6)	O(4) , S(1)a	165.92(7)	O(4) , N(1)	56.88(6)	O(4) , O(4)a	121.84(7)
C(16) , C(17)a	84.85(7)	C(16) , C(17)	23.83(6)	C(16) , S(1)	54.10(5)	C(16) , S(1)a	102.56(7)
C(16) , N(1)	19.86(5)	C(16) , O(4)a	120.50(8)	C(17)a , C(17)	97.67(6)	C(17)a , S(1)	103.89(5)
C(17)a , S(1)a	31.04(3)	C(17)a , N(1)	84.86(5)	C(17)a , O(4)a	53.65(5)	C(17) , S(1)	30.96(3)
C(17) , S(1)a	104.19(5)	C(17) , N(1)	41.99(4)	C(17) , O(4)a	143.08(6)	S(1) , S(1)a	93.85(4)
S(1) , N(1)	72.93(4)	S(1) , O(4)a	157.02(5)	S(1)a , N(1)	110.09(5)	S(1)a , O(4)a	64.47(4)
N(1) , O(4)a	106.17(5)						

3.6 Angstrom Coordination Sphere Around Atom I = O(6) [ARU = 1555.01] 0.71030 0.66903 0.52069 3.5619 12.5024 13.2445

Nr	d(I,J)	To	Atom J	Symm_Oper. on Atom J	ARU(J)	Type	Phi	Mu	X	Y	Z	XO	YO	ZO
1	1.228(2)	--	C(20)			Intra	-138.59	5.28	0.52740	0.62575	0.52513	2.6448	11.6936	13.3575
2	2.269(2)	<<	N(2)			Intra	-147.51	33.69	0.39270	0.61475	0.57017	1.9693	11.4880	14.5031
3	2.380(2)	<<	C(10)			Intra	-131.58	-27.28	0.43040	0.58437	0.47781	2.1583	10.9203	12.1538
4	2.773(2)	<<	C(21)			Intra	-161.43	64.01	0.48060	0.64832	0.61868	2.4101	12.1154	15.7371
5	2.9539(18)	.<	O(2)			Intra	-74.69	4.56	0.86530	0.51705	0.52992	4.3392	9.6623	13.4793
6	3.018(2)	<<	C(22)			Intra	138.87	55.20	0.45160	0.72966	0.61812	2.2646	13.6354	15.7228
7	3.242(2)	..	C(11)			Intra	-157.01	-33.79	0.21570	0.61272	0.44981	1.0817	11.4501	11.4416
8	3.242(2)	..	C(9)			Intra	-105.62	-28.88	0.55790	0.52272	0.45912	2.7977	9.7682	11.6784
9	3.284(2)	..	C(11)a	[1+x,y,z	= 1655.01]		-22.55	-33.30	1.21570	0.61272	0.44981	6.0964	11.4501	11.4416
10	3.474(2)	..	C(8)			Intra	-84.63	-12.67	0.77360	0.48843	0.49073	3.8794	9.1274	12.4825
11	2.39	<<	H(18)			Intra	120.20	44.02	0.53787	0.74852	0.58598	2.6973	13.9878	14.9053
12	2.54	.<	H(8)a	[1+x,y,z	= 1655.01]		-8.52	-34.91	1.12084	0.65253	0.46358	5.6207	12.1940	11.7919
13	2.64	.<	H(2)a	[1+x,y,z	= 1655.01]		39.43	36.76	1.03550	0.74079	0.58269	5.1927	13.8434	14.8216
14	2.69	.<	H(17)			Intra	-106.94	76.47	0.67370	0.63678	0.62365	3.3784	11.8997	15.8635
15	3.04	..	H(4)			Intra	-146.40	24.49	0.25072	0.58708	0.57024	1.2573	10.9709	14.5049
16	3.24	..	H(2)b	[1/2+x,3/2-y,1-z	= 3566.01]		117.48	-54.16	0.53550	0.75921	0.41731	2.6854	14.1876	10.6149
17	3.31	..	H(8)			Intra	-174.04	-26.05	0.12084	0.65253	0.46358	0.6060	12.1940	11.7919
18	3.36	..	H(4)a	[1+x,y,z	= 1655.01]		-29.47	22.04	1.25072	0.58708	0.57024	6.2720	10.9709	14.5049
19	3.39	..	H(8)b	[1/2+x,3/2-y,1-z	= 3566.01]		97.66	6.78	0.62084	0.84747	0.53642	3.1133	15.8369	13.6446
20	3.53	..	H(18)b	[1/2+x,3/2-y,1-z	= 3566.01]		43.17	-50.31	1.03787	0.75148	0.41402	5.2046	14.0431	10.5312

Angles (Degrees) At1...V...At2 with Vertex V = O(6)

C(20) , N(2)	29.59(10)	C(20) , C(10)	33.26(10)	C(20) , C(21)	61.00(11)	C(20) , O(2)	63.64(11)
C(20) , C(22)	81.41(11)	C(20) , C(11)	42.77(10)	C(20) , C(9)	46.61(10)	C(20) , C(11)a	114.58(12)
C(20) , C(8)	56.54(11)	N(2) , C(10)	62.81(7)	N(2) , C(21)	31.52(6)	N(2) , O(2)	73.19(6)
N(2) , C(22)	53.89(6)	N(2) , C(11)	68.06(6)	N(2) , C(9)	74.07(6)	N(2) , C(11)a	134.67(7)
N(2) , C(8)	75.62(6)	C(10) , C(21)	94.26(7)	C(10) , O(2)	63.41(6)	C(10) , C(22)	111.87(8)
C(10) , C(11)	22.78(6)	C(10) , C(9)	22.92(6)	C(10) , C(11)a	89.46(7)	C(10) , C(8)	46.18(6)
C(21) , O(2)	84.47(6)	C(21) , C(22)	30.20(5)	C(21) , C(11)	97.86(7)	C(21) , C(9)	102.63(6)
C(21) , C(11)a	140.31(7)	C(21) , C(8)	95.71(6)	O(2) , C(22)	114.13(6)	O(2) , C(11)	86.18(5)
O(2) , C(9)	44.73(4)	O(2) , C(11)a	62.12(5)	O(2) , C(8)	19.86(4)	C(22) , C(11)	104.46(7)
C(22) , C(9)	127.73(7)	C(22) , C(11)a	154.55(7)	C(22) , C(8)	125.74(6)	C(11) , C(9)	43.72(5)
C(11) , C(11)a	100.44(6)	C(11) , C(8)	68.45(5)	C(9) , C(11)a	69.30(5)	C(9) , C(8)	25.37(4)
C(11)a , C(8)	59.85(5)						

3.6 Angstrom Coordination Sphere Around Atom I = O(7) [ARU = 1555.01] 0.16140 0.56930 0.65940 0.8094 10.6387 16.7728

Nr	d(I,J)	To	Atom J	Symm_Oper. on Atom J	ARU(J)	Type	Phi	Mu	X	Y	Z	XO	YO	ZO
1	1.209(2)	--	C(23)			Intra	46.19	5.60	0.32750	0.61577	0.66404	1.6423	11.5071	16.8909
2	2.2461(19)	<<	O(8)			Intra	50.54	34.14	0.39700	0.64610	0.70896	1.9908	12.0739	18.0335
3	2.412(2)	<<	C(21)			Intra	42.69	-25.44	0.48060	0.64832	0.61868	2.4101	12.1154	15.7371
4	2.664(2)	<<	C(24)			Intra	65.91	65.82	0.25020	0.62259	0.75493	1.2547	11.6345	19.2028
5	2.687(2)	<<	N(2)			Intra	36.21	-57.65	0.39270	0.61475	0.57017	1.9693	11.4880	14.5031
6	3.214(2)	.<	C(16)a	[-1+x,y,z	= 1455.01]		-107.60	-4.21	-0.03190	0.40581	0.65013	-0.1600	7.5835	16.5370
7	3.229(2)	..	C(17)a	[-1+x,y,z	= 1455.01]		-133.17	5.63	-0.27700	0.44389	0.67185	-1.3891	8.2951	17.0895
8	3.493(2)	..	C(22)			Intra	64.10	-17.49	0.45160	0.72966	0.61812	2.2646	13.6354	15.7228
9	2.34	<<	H(4)			Intra	36.57	-76.18	0.25072	0.58708	0.57024	1.2573	10.9709	14.5049
10	2.46	<<	H(21)			Intra	118.29	64.10	0.05972	0.61999	0.74650	0.2995	11.5859	18.9883
11	2.48	<<	H(11)a	[-1+x,y,z	= 1455.01]		-93.82	-2.69	0.12845	0.43696	0.65482	0.6441	8.1656	16.6563
12	2.69	.<	H(13)a	[-1+x,y,z	= 1455.01]		-147.37	-2.27	-0.29067	0.49163	0.65520	-1.4576	9.1872	16.6660
13	2.81	..	H(20)			Intra	8.23	74.06	0.31375	0.57521	0.76568	1.5734	10.7491	19.4762
14	2.90	..	H(17)a	[-1+x,y,z	= 1455.01]		152.72	-18.29	-0.32630	0.63678	0.62365	-1.6363	11.8997	15.8635
15	3.00	..	H(17)			Intra	26.14	-17.63	0.67370	0.63678	0.62365	3.3784	11.8997	15.8635
16	3.01	..	H(1)a	[-1+x,y,z	= 1455.01]		-133.80	47.76	-0.11807	0.49110	0.74706	-0.5921	9.1773	19.0026
17	3.11	..	H(13)			Intra	-27.84	-1.97	0.70933	0.49163	0.65520	3.5571	9.1872	16.6660
18	3.49	..	H(12)			Intra	-54.90	1.04	0.56210	0.41633	0.66189	2.8188	7.7801	16.8362

Angles (Degrees) At1...V...At2 with Vertex V = O(7)

C(23) ,	O(8)	28.82(10)	C(23) ,	C(21)	31.22(10)	C(23) ,	C(24)	61.79(11)	C(23) ,	N(2)	63.77(11)
C(23) ,	C(16)a	153.86(13)	C(23) ,	C(17)a	168.75(12)	C(23) ,	C(22)	29.09(10)	O(8) ,	C(21)	60.04(6)
O(8) ,	C(24)	32.98(7)	O(8) ,	N(2)	92.58(7)	O(8) ,	C(16)a	143.83(7)	O(8) ,	C(17)a	140.07(7)
O(8) ,	C(22)	53.23(5)	C(21) ,	C(24)	92.98(8)	C(21) ,	N(2)	32.54(6)	C(21) ,	C(16)a	138.66(7)
C(21) ,	C(17)a	159.80(7)	C(21) ,	C(22)	21.41(5)	C(24) ,	N(2)	125.47(8)	C(24) ,	C(16)a	118.21(7)
C(24) ,	C(17)a	107.20(8)	C(24) ,	C(22)	83.33(7)	N(2) ,	C(16)a	111.64(6)	N(2) ,	C(17)a	127.32(6)
N(2) ,	C(22)	45.17(5)	C(16)a ,	C(17)a	27.36(5)	C(16)a ,	C(22)	156.80(6)	C(17)a ,	C(22)	159.37(7)

3.6 Angstrom Coordination Sphere Around Atom I = O(8) [ARU = 1555.01] 0.39700 0.64610 0.70896 1.9908 12.0739 18.0335

Nr	d(I,J)	To	Atom J	Symm_Oper. on Atom J	ARU(J)	Type	Phi	Mu	X	Y	Z	XO	YO	ZO
1	1.322(2)	--	C(23)			Intra	-121.59	-59.79	0.32750	0.61577	0.66404	1.6423	11.5071	16.8909
2	1.450(3)	--	C(24)			Intra	-149.17	53.75	0.25020	0.62259	0.75493	1.2547	11.6345	19.2028
3	2.2461(19)	<<	O(7)			Intra	-129.46	-34.14	0.16140	0.56930	0.65940	0.8094	10.6387	16.7728
4	2.335(2)	<<	C(21)			Intra	5.65	-79.60	0.48060	0.64832	0.61868	2.4101	12.1154	15.7371
5	2.802(2)	<<	C(22)			Intra	80.05	-55.55	0.45160	0.72966	0.61812	2.2646	13.6354	15.7228
6	3.187(3)	.<	C(19)b	[2-x,1/2+y,3/2-z	= 4756.01]		55.89	23.46	0.72400	0.77564	0.75884	3.6307	14.4946	19.3022
7	3.4342(14)	..	S(2)			Intra	123.85	-41.23	0.11009	0.76088	0.61998	0.5521	14.2188	15.7701
8	3.579(2)	..	N(2)			Intra	-92.11	-80.57	0.39270	0.61475	0.57017	1.9693	11.4880	14.5031
9	2.00	<<	H(21)			Intra	-163.91	28.48	0.05972	0.61999	0.74650	0.2995	11.5859	18.9883
10	2.00	<<	H(20)			Intra	-107.49	46.09	0.31375	0.57521	0.76568	1.5734	10.7491	19.4762
11	2.00	<<	H(22)			Intra	161.70	71.67	0.27776	0.65668	0.78370	1.3929	12.2716	19.9346
12	2.58	.<	H(19)			Intra	68.62	-36.40	0.54800	0.74958	0.64876	2.7481	14.0076	16.5022
13	2.58	.<	H(17)			Intra	-7.15	-57.20	0.67370	0.63678	0.62365	3.3784	11.8997	15.8635
14	2.73	..	H(15)b	[2-x,1/2+y,3/2-z	= 4756.01]		73.43	19.13	0.54362	0.77836	0.74413	2.7261	14.5454	18.9281
15	2.98	..	H(16)b	[2-x,1/2+y,3/2-z	= 4756.01]		43.18	37.65	0.74005	0.73250	0.78052	3.7111	13.6884	19.8537
16	3.35	..	H(14)b	[2-x,1/2+y,3/2-z	= 4756.01]		46.12	9.26	0.85431	0.77370	0.73017	4.2841	14.4584	18.5730
17	3.49	..	H(21)a	[1+x,y,z	= 1655.01]		-8.35	15.87	1.05972	0.61999	0.74650	5.3142	11.5859	18.9883
18	3.56	..	H(13)			Intra	-61.52	-22.61	0.70933	0.49163	0.65520	3.5571	9.1872	16.6660

Angles (Degrees) At1...V...At2 with Vertex V = O(8)

C(23)	C(24)	115.67(16)	C(23)	O(7)	26.16(9)	C(23)	C(21)	37.34(10)	C(23)	C(22)	63.39(10)
C(23)	C(19)b	143.63(12)	C(23)	S(2)	65.66(9)	C(23)	N(2)	22.45(9)	C(24)	O(7)	89.54(12)
C(24)	C(21)	152.85(14)	C(24)	C(22)	152.06(14)	C(24)	C(19)b	99.81(12)	C(24)	S(2)	120.54(13)
C(24)	N(2)	137.98(12)	O(7)	C(21)	63.50(7)	O(7)	C(22)	86.83(7)	O(7)	C(19)b	168.34(8)
O(7)	S(2)	78.98(5)	O(7)	N(2)	48.59(5)	C(21)	C(22)	33.02(6)	C(21)	C(19)b	106.60(8)
C(21)	S(2)	54.26(5)	C(21)	N(2)	14.92(5)	C(22)	C(19)b	81.65(7)	C(22)	S(2)	31.73(4)
C(22)	N(2)	43.81(5)	C(19)b	S(2)	90.20(6)	C(19)b	N(2)	121.34(7)	S(2)	N(2)	56.60(3)

3.6 Angstrom Coordination Sphere Around Atom I = N(1) [ARU = 1555.01] 0.92720 0.39175 0.59414 4.6496 7.3207 15.1128

Nr	d(I,J)	To	Atom J	Symm_Oper. on Atom J	ARU(J)	Type	Phi	Mu	X	Y	Z	XO	YO	ZO
1	0.88	--	H(3)			Intra	-166.23	-19.08	0.76613	0.38116	0.58283	3.8419	7.1229	14.8252
2	1.339(2)	--	C(15)			Intra	3.00	-40.93	1.12870	0.39458	0.55965	5.6601	7.3736	14.2355
3	1.463(2)	--	C(16)			Intra	52.02	76.83	0.96810	0.40581	0.65013	4.8547	7.5835	16.5370
4	2.261(2)	<<	O(3)			Intra	5.35	-14.02	1.36280	0.40269	0.57261	6.8340	7.5252	14.5652
5	2.419(2)	<<	C(6)			Intra	-19.77	-72.91	1.06060	0.37888	0.50325	5.3186	7.0802	12.8009
6	2.430(2)	<<	C(17)			Intra	136.42	54.43	0.72300	0.44389	0.67185	3.6256	8.2951	17.0895
7	2.443(2)	<<	C(18)			Intra	-68.47	62.34	1.01020	0.33530	0.67921	5.0659	6.2659	17.2767
8	2.8719(19)	.<	O(2)			Intra	97.55	-34.67	0.86530	0.51705	0.52992	4.3392	9.6623	13.4793
9	2.890(2)	.<	O(3)a	[-1+x,y,z	= 1455.01]		175.87	-10.92	0.36280	0.40269	0.57261	1.8193	7.5252	14.5652
10	2.901(2)	.<	O(4)			Intra	-99.03	45.68	0.86380	0.28464	0.67573	4.3317	5.3192	17.1882
11	3.154(2)	.<	C(7)			Intra	114.77	-80.10	0.88190	0.41809	0.47201	4.4225	7.8130	12.0063
12	3.283(2)	..	C(8)			Intra	113.09	-53.25	0.77360	0.48843	0.49073	3.8794	9.1274	12.4825
13	3.431(3)	..	C(5)			Intra	-45.44	-56.67	1.19090	0.31988	0.48145	5.9720	5.9777	12.2464
14	3.436(2)	..	O(5)			Intra	-33.77	58.36	1.22600	0.33814	0.70915	6.1480	6.3189	18.0383
15	2.03	<<	H(11)			Intra	39.93	49.55	1.12845	0.43696	0.65482	5.6588	8.1656	16.6563
16	2.56	.<	H(12)			Intra	165.92	42.39	0.56210	0.41633	0.66189	2.8188	7.7801	16.8362
17	2.66	.<	H(13)			Intra	120.34	35.68	0.70933	0.49163	0.65520	3.5571	9.1872	16.6660
18	2.97	..	H(6)b	[-1/2+x,1/2-y,1-z	= 3456.01]		-109.38	-5.13	0.73158	0.24248	0.58370	3.6687	4.5313	14.8473
19	3.55	..	H(7)			Intra	-42.89	-41.25	1.31750	0.29446	0.50203	6.6069	5.5027	12.7699

Angles (Degrees) At1...V...At2 with Vertex V = N(1)

C(15) ,	C(16)	121.67(16)	C(15) ,	O(3)	26.99(10)	C(15) ,	C(6)	33.81(10)	C(15) ,	C(17)	146.61(13)
C(15) ,	C(18)	117.95(13)	C(15) ,	O(2)	71.14(10)	C(15) ,	O(3)a	127.73(12)	C(15) ,	O(4)	125.35(12)
C(15) ,	C(7)	53.33(10)	C(15) ,	C(8)	68.31(10)	C(15) ,	C(5)	34.64(9)	C(15) ,	O(5)	103.90(11)
C(16) ,	O(3)	94.83(11)	C(16) ,	C(6)	155.47(13)	C(16) ,	C(17)	36.39(10)	C(16) ,	C(18)	36.03(9)
C(16) ,	O(2)	115.00(10)	C(16) ,	O(3)a	108.01(11)	C(16) ,	O(4)	56.13(9)	C(16) ,	C(7)	160.26(11)
C(16) ,	C(8)	135.58(11)	C(16) ,	C(5)	146.08(12)	C(16) ,	O(5)	33.10(8)	O(3) ,	C(6)	60.68(7)
O(3) ,	C(17)	124.60(9)	O(3) ,	C(18)	95.10(8)	O(3) ,	O(2)	83.85(7)	O(3) ,	O(3)a	153.35(8)
O(3) ,	O(4)	109.97(8)	O(3) ,	C(7)	79.45(7)	O(3) ,	C(8)	89.02(7)	O(3) ,	C(5)	57.36(6)
O(3) ,	O(5)	79.12(6)	C(6) ,	C(17)	159.05(9)	C(6) ,	C(18)	139.16(9)	C(6) ,	O(2)	64.36(6)
C(6) ,	O(3)a	95.55(7)	C(6) ,	O(4)	130.21(8)	C(6) ,	C(7)	25.02(6)	C(6) ,	C(8)	49.74(6)
C(6) ,	C(5)	19.23(5)	C(6) ,	O(5)	131.62(8)	C(17) ,	C(18)	61.61(7)	C(17) ,	O(2)	95.18(7)
C(17) ,	O(3)a	73.33(7)	C(17) ,	O(4)	69.42(7)	C(17) ,	C(7)	135.10(8)	C(17) ,	C(8)	109.40(7)
C(17) ,	C(5)	177.52(8)	C(17) ,	O(5)	66.93(6)	C(18) ,	O(2)	150.96(8)	C(18) ,	O(3)a	111.42(8)
C(18) ,	O(4)	24.08(6)	C(18) ,	C(7)	162.22(8)	C(18) ,	C(8)	170.87(8)	C(18) ,	C(5)	120.36(8)
C(18) ,	O(5)	17.39(5)	O(2) ,	O(3)a	74.26(6)	O(2) ,	O(4)	163.28(7)	O(2) ,	C(7)	45.94(5)
O(2) ,	C(8)	21.59(4)	O(2) ,	C(5)	83.43(6)	O(2) ,	O(5)	140.28(6)	O(3)a ,	O(4)	94.42(7)
O(3)a ,	C(7)	74.44(6)	O(3)a ,	C(8)	65.13(6)	O(3)a ,	C(5)	104.29(6)	O(3)a ,	O(5)	127.52(6)
O(4) ,	C(7)	143.57(7)	O(4) ,	C(8)	158.02(7)	O(4) ,	C(5)	111.71(6)	O(4) ,	O(5)	40.32(5)
C(7) ,	C(8)	26.85(4)	C(7) ,	C(5)	42.76(5)	C(7) ,	O(5)	156.29(7)	C(8) ,	C(5)	68.68(5)

==

C(8)	, O(5)	160.90(6)	C(5)	, O(5)	115.41(6)

3.6 Angstrom Coordination Sphere Around Atom I = N(2) [ARU = 1555.01] 0.39270 0.61475 0.57017 1.9693 11.4880 14.5031

Nr	d(I,J)	To	Atom J	Symm_Oper. on Atom J	ARU(J)	Type	Phi	Mu	X	Y	Z	XO	YO	ZO
1	0.88	--	H(4)			Intra	-144.01	0.12	0.25072	0.58708	0.57024	1.2573	10.9709	14.5049
2	1.346(2)	--	C(20)			Intra	16.93	-58.35	0.52740	0.62575	0.52513	2.6448	11.6936	13.3575
3	1.453(2)	--	C(21)			Intra	54.91	58.14	0.48060	0.64832	0.61868	2.4101	12.1154	15.7371
4	2.269(2)	<<	O(6)			Intra	32.49	-33.69	0.71030	0.66903	0.52069	3.5619	12.5024	13.2445
5	2.410(2)	<<	C(23)			Intra	176.66	82.19	0.32750	0.61577	0.66404	1.6423	11.5071	16.8909
6	2.424(2)	<<	C(10)			Intra	-71.58	-75.71	0.43040	0.58437	0.47781	2.1583	10.9203	12.1538
7	2.487(2)	<<	C(22)			Intra	82.17	29.37	0.45160	0.72966	0.61812	2.2646	13.6354	15.7228
8	2.687(2)	<<	O(7)			Intra	-143.79	57.65	0.16140	0.56930	0.65940	0.8094	10.6387	16.7728
9	3.162(2)	..	O(2)			Intra	-37.61	-18.89	0.86530	0.51705	0.52992	4.3392	9.6623	13.4793
10	3.188(2)	.<	C(11)			Intra	-177.55	-73.82	0.21570	0.61272	0.44981	1.0817	11.4501	11.4416
11	3.3273(16)	.<	S(2)			Intra	117.43	22.38	0.11009	0.76088	0.61998	0.5521	14.2188	15.7701
12	3.373(2)	..	O(2)a	[-1+x,y,z	= 1455.01]		-145.38	-17.67	-0.13470	0.51705	0.52992	-0.6755	9.6623	13.4793
13	3.409(2)	..	C(9)			Intra	-64.28	-55.95	0.55790	0.52272	0.45912	2.7977	9.7682	11.6784
14	3.579(2)	..	O(8)			Intra	87.89	80.57	0.39700	0.64610	0.70896	1.9908	12.0739	18.0335
15	2.00	<<	H(17)			Intra	16.29	42.82	0.67370	0.63678	0.62365	3.3784	11.8997	15.8635
16	2.63	.<	H(18)			Intra	73.76	8.78	0.53787	0.74852	0.58598	2.6973	13.9878	14.9053
17	2.98	..	H(2)			Intra	127.25	6.14	0.03550	0.74079	0.58269	0.1780	13.8434	14.8216
18	3.12	..	H(8)			Intra	152.62	-60.48	0.12084	0.65253	0.46358	0.6060	12.1940	11.7919
19	3.31	..	H(19)			Intra	72.82	37.16	0.54800	0.74958	0.64876	2.7481	14.0076	16.5022
20	3.53	..	H(13)			Intra	-55.39	37.73	0.70933	0.49163	0.65520	3.5571	9.1872	16.6660

Angles (Degrees) At1...V...At2 with Vertex V = N(2)

C(20)	C(21)	120.32(15)	C(20)	O(6)	26.78(9)	C(20)	C(23)	155.55(14)	C(20)	C(10)	34.07(10)
C(20)	C(22)	103.06(12)	C(20)	O(7)	169.79(12)	C(20)	O(2)	55.69(10)	C(20)	C(11)	47.47(10)
C(20)	S(2)	114.37(11)	C(20)	O(2)a	102.58(11)	C(20)	C(9)	41.39(9)	C(20)	O(8)	144.27(12)
C(21)	O(6)	93.73(11)	C(21)	C(23)	36.51(9)	C(21)	C(10)	154.23(13)	C(21)	C(22)	34.37(9)
C(21)	O(7)	63.25(10)	C(21)	O(2)	107.27(11)	C(21)	C(11)	154.87(11)	C(21)	S(2)	56.73(9)
C(21)	O(2)a	136.85(11)	C(21)	C(9)	147.98(12)	C(21)	O(8)	24.44(9)	O(6)	C(23)	129.88(9)
O(6)	C(10)	60.82(7)	O(6)	C(22)	78.62(7)	O(6)	O(7)	155.90(8)	O(6)	O(2)	63.42(5)
O(6)	C(11)	70.62(6)	O(6)	S(2)	98.24(6)	O(6)	O(2)a	128.60(7)	O(6)	C(9)	66.13(6)
O(6)	O(8)	118.02(7)	C(23)	C(10)	166.53(9)	C(23)	C(22)	61.54(7)	C(23)	O(7)	26.74(6)
C(23)	O(2)	115.28(7)	C(23)	C(11)	156.04(9)	C(23)	S(2)	63.80(5)	C(23)	O(2)a	101.46(7)
C(23)	C(9)	149.07(8)	C(23)	O(8)	12.09(5)	C(10)	C(22)	131.92(9)	C(10)	O(7)	141.11(8)
C(10)	O(2)	59.51(6)	C(10)	C(11)	24.25(6)	C(10)	S(2)	126.47(7)	C(10)	O(2)a	68.91(6)
C(10)	C(9)	19.95(5)	C(10)	O(8)	173.64(8)	C(22)	O(7)	84.83(7)	C(22)	O(2)	124.63(8)
C(22)	C(11)	120.95(8)	C(22)	S(2)	32.36(5)	C(22)	O(2)a	135.18(8)	C(22)	C(9)	144.39(8)
C(22)	O(8)	51.26(6)	O(7)	O(2)	114.49(6)	O(7)	C(11)	133.42(7)	O(7)	S(2)	75.75(5)
O(7)	O(2)a	75.33(5)	O(7)	C(9)	130.20(7)	O(7)	O(8)	38.83(4)	O(2)	C(11)	83.73(6)
O(2)	S(2)	156.41(6)	O(2)	O(2)a	100.19(5)	O(2)	C(9)	42.13(4)	O(2)	O(8)	114.17(5)
C(11)	S(2)	104.89(5)	C(11)	O(2)a	58.92(5)	C(11)	C(9)	42.77(5)	C(11)	O(8)	162.00(7)

S(2)	,	O(2)a	103.05(5)	S(2)	,	C(9)	146.41(6)	S(2)	,	O(8)	59.51(3)
O(2)a	,	C(9)	70.49(5)	O(2)a	,	O(8)	113.13(5)	C(9)	,	O(8)	153.96(6)

Search for and Analysis of Solvent Accessible Voids in the Structure - Grid = 0.20 Ang., Probe Radius = 1.20 Ang., NStep = 6

van der Waals (or ion) Radii used in the Analysis

C	H	N	O	S
1.70	1.20	1.55	1.52	1.80

:: Grid: X-Axis Step = 0.0417 = Points 24, Angstrom Step = 0.21

:: Grid: Y-Axis Step = 0.0104 = Points 96, Angstrom Step = 0.19

:: Grid: Z-Axis Step = 0.0076 = Points 132, Angstrom Step = 0.19

:: Unit cell Contains NO Residual Solvent Accessible Void.

:: Note: use CALC VOID (not CALC SOLV) for Packing Index.

==

Report Expected Number of Independent Reflections for given Symmetry and Resolution.

:: Hmax = 6 Kmax = 24 Lmax= 33 , Sorting Order : Fast H, Medium K, Slow L

:: Actual Theta-Max: 27.480 Deg. (Applied Theta Limit: 27.480 Deg.)

Space Group H-M: P212121   Laue: mmm
Space Group Hall: P 2ac 2ab   [Schoenflies: D2^4]
Lattice Type: oP, Acentric, Orthorhombic, Multiplicity: 4(4), No: 19

CHIRAL - See P.G. Jones, Acta Cryst. (1986), A42, 57.

Nr	***** Symmetry Operation(s) *****		
1	H ,	K ,	L
2	1/2 - H ,	- K ,	1/2 + L
3	1/2 + H ,	1/2 - K ,	- L
4	- H ,	1/2 + K ,	1/2 - L

:: Number of Independent   Type H, K, L Reflections =   3184
:: Number of Independent   Type -H,-K,-L Reflections =   2292

Table 0 - Crystal Data and Details of the Structure Determination
for: A-Co P 21 21 21 R = 0.04

Crystal Data

Formula			C24 H22 N2 O8 S2
Formula Weight			530.57
Crystal System			orthorhombic
Space group		P212121	(No. 19)
a, b, c [Angstrom]	5.01471(10)	18.6873(3)	25.4365(6)
V [Ang**3]			2383.69(8)
Z			4
D(calc) [g/cm**3]			1.478
Mu(MoKa) [/mm]			0.277
F(000)			1104
Crystal Size [mm]			0.03 x 0.10 x 0.16

Data Collection

Temperature (K)			123
Radiation [Angstrom]		MoKa	0.71075
Theta Min-Max [Deg]			3.2, 27.5
Dataset			-6: 6 ; -23: 24 ; -32: 32
Tot., Uniq. Data, R(int)			23904, 5463, 0.037
Observed Data [I > 0.0 sigma(I)]			5041

Refinement

Nref, Npar	5463, 335
R, wR2, S	0.0362, 0.0925, 1.08
w = ¥S^2^(FO^2^)+(0.0544P)^2^+0.4090P] WHERE P=(FO^2^+2FC^2^)/3'	
Max. and Av. Shift/Error	0.00, 0.00
Flack x	0.00(6)
Min. and Max. Resd. Dens. [e/Ang^3]	-0.25, 0.43

===========
** N O T I C E **
===========

- PLATON Reference : Spek, A.L. (2003). J. Appl. Cryst. 36, 7-13.
  Spek, A.L. (2009). Acta Cryst. D65, 148-155.

- Output Values (Esd) may have been set to 99, 999 or 9999 to Avoid Format Overflow

- Derived Parameter SU's (= Esd's) may be Incorrect in Cases where Covariances in the Atom Parameters should have been taken into Account (e.g. Those Involving Atoms That were Refined with Constraints)

- ROUNDING, in particular of the Input Coordinate Data, may give deviating values for derived geometry parameters. However, differences should be within the associated esd-range.

- PLATON is NOT a Finished Program. The Implementation of Additional Options is Planned. Some of the More Advanced Features are Experimental and may Contain Loose Ends.

- The Communication of Glitches Encountered will be Appreciated: E-mail: a.l.spek@uu.nl

- Recent versions of PLATON may be obtained from http://www.platonsoft.nl/xraysoft

- More INFO can be found on http://http://www.platonsoft.nl/

==

Page - Index

==

Page   1 --- GENERAL
Page   2 --- ADDSYM
Page   3 --- GEOMETRY
Page   6 --- MOLSYM
Page   8 --- NONSYM
Page   9 --- ADP-Anal
Page 19 --- GEOMETRY
Page 45 --- INTER
Page 61 --- COORDN
Page 77 --- VOIDS
Page 78 --- EXPECT
Page 79 --- SUMMARY

Summary and Remarks : N = NOTE, W = WARNING, E = ERROR

==

W: NOMOVE option used.
:: >>> WARNING: 'CONNECTED INPUT SET' is assumed to be TRUE
:: >>> The Network Analysis may be INCORRECT when this assumption is FALSE

N: Number of Ignored Lines on INPUT   2
          of which blank in column 1   0
N: Number of Non-HBonded D-H atoms   2

==

:: Input Xtal Data from File revisedcif.cif - Data Type CIF

:: NORMAL END of PLATON :   81 Pages on FILE revisedcif.lis

:: SPF File spf on :revisedcif_pl.spf
