# Supplementary material for: Synthesis of a Novel Cysteine-Incorporated Anthraquinone Derivative and Its Structural Properties
Source: Molecules. 2015 Jun 3;20(6):10192–204. doi: 10.3390/molecules200610192 (PMC6272162; doi:10.3390/molecules200610192)
Supplement: Supplementary file 1 [file molecules-20-10192-s004.zip › molecules-80292-supplementary-2 - final.pdf]

X-ray Structure Report

for

7

Experimental

Data Collection

A yellow platelet crystal of $C_{24}H_{22}N_2O_8S_2$ having approximate dimensions of 0.160 × 0.100 × 0.030 mm was mounted on a glass fiber. All measurements were made on a Rigaku R-AXIS RAPID diffractometer using filtered Mo-Kα radiation.

The crystal-to-detector distance was 127.40 mm.

Cell constants and an orientation matrix for data collection corresponded to a primitive orthorhombic cell with dimensions:

a = 5.01471(10) Å
b = 18.6873(3) Å
c = 25.4365(6) Å
V = 2383.70(8) $Å^3$

For Z = 4 and F.W. = 530.57, the calculated density is 1.478 g/cm^3. The reflection conditions of:

h00: h = 2n
0k0: k = 2n
00l: l = 2n

uniquely determine the space group to be:

$P2_12_12_1$ (#19)

The data were collected at a temperature of −150 ± 1 °C to a maximum 2θ value of 55.0°. A total of 111 oscillation images were collected. A sweep of data was done using ω scans from 130.0 to 190.0° in 2.00° step, at χ = 45.0° and ϕ = 0.0°. The exposure rate was 40.0 [s/°]. A second sweep was performed using ω scans from 0.0 to 162.0° in 2.00° step, at χ = 45.0° and ϕ = 180.0°. The exposure rate was 40.0 [s/°]. The crystal-to-detector distance was 127.40 mm. Readout was performed in the 0.100 mm pixel mode.

Data Reduction

Of the 23904 reflections were collected, where 5463 were unique (R_{int} = 0.0368); equivalent reflections were merged.

The linear absorption coefficient, μ, for Mo-Kα radiation is 2.771 cm^{-1}. An empirical absorption correction was applied which resulted in transmission factors ranging from 0.823 to 0.992. The data were corrected for Lorentz and polarization effects.

Structure Solution and Refinement

The structure was solved by direct methods [1] and expanded using Fourier techniques. The non-hydrogen atoms were refined anisotropically. Hydrogen atoms were refined using the riding model. The final cycle of full-matrix least-squares refinement [2] on F^2 was based on 5463 observed reflections and 335 variable parameters and converged (largest parameter shift was 0.00 times its esd) with unweighted and weighted agreement factors of:

$$R1 = \Sigma ||Fo| - |Fc||/\Sigma |Fo| = 0.0362$$

$$wR2 = [\Sigma (w (Fo^2 - Fc^2)^2)/\Sigma w(Fo^2)^2]^{1/2} = 0.0925$$

The goodness of fit [3] was 1.08. Unit weights were used. Plots of $\Sigma w (|Fo| - |Fc|)^2$ *vs.* |Fo|, reflection order in data collection, sin θ/λ and various classes of indices showed no unusual trends. The maximum and minimum peaks on the final difference Fourier map corresponded to 0.43 and −0.25 $e^-/Å^3$, respectively. The final Flack parameter [4] was −0.00(6), indicating that the present absolute structure is correct [5].

Neutral atom scattering factors were taken from International Tables for Crystallography (IT), Vol. C, Table 6.1.1.4 [6]. Anomalous dispersion effects were included in Fcalc [7]; the values for Δf′ and Δf″ were those of Creagh and McAuley [8]. The values for the mass attenuation coefficients are those of Creagh and Hubbell [9]. All calculations were performed using the CrystalStructure [10] crystallographic software package except for refinement, which was performed using SHELXL97 [11].

References and Notes

1. SIR2008: Burla, M.C.; Caliandro, R.; Camalli, M.; Carrozzini, B.; Cascarano, G.L.; de Caro, L.; Giacovazzo, C., Polidori, G.; Siliqi, D.; Spagna R. IL MILIONE: A suite of computer programs for crystal structure solution of proteins. *J. Appl. Crystallogr.* **2007**, *40*, 609–613.
2. Least Squares function minimized: (SHELXL97)

   $\Sigma w(F_O^2 - F_C^2)^2$ where w = Least Squares weights.

3. Goodness of fit is defined as:

   $[\Sigma w(F_O^2 - F_C^2)^2/(N_O - N_V)]^{1/2}$

   where: N_O = number of observations
   N_V = number of variables

4. Flack, H.D. On enantiomorph-polarity estimation. *Acta Crystallogr.* **1983**, *A39*, 876–881.
5. Flack, H.D.; Bernardinelli, G. Reporting and evaluating absolute-structure and absolute-configuration determinations. *J. Appl. Crystallogr.* **2000**, *33*, 1143–1148.
6. Wilson, A.J.C. Tables 6.1.1.4. In *International Tables for X-ray Crystallography*; Kluwer Academic Publishers: Dordrecht, The Netherlands, 1992; Volume C, pp. 500–502.
7. Ibers, J.A.; Hamilton, W.C. Dispersion corrections and crystal structure refinements. *Acta Crystallogr.* **1964**, *17*, 781–782.
8. Creagh, D.C.; McAuley, W.J. Table 4.2.6.8. In *International Tables for Crystallography*; Wilson, A.J.C., Ed.; Kluwer Academic Publishers: Boston, MA, USA, 1992; Volume C, pp. 219–222.
9. Creagh, D.C.; Hubbell, J.H. Table 4.2.4.3. In *International Tables for Crystallography*; Wilson, A.J.C., Ed.; Kluwer Academic Publishers: Boston, MA, USA, 1992; Volume C, pp. 200–206.
10. *CrystalStructure 4.1:* Crystal Structure Analysis Package; Rigaku Corporation (2000–2014): Tokyo, Japan, 2014.
11. SHELXL97: Sheldrick, G.M. A short history of SHELX. *Acta Crystallogr.* **2008**, *A64*, 112–122.

Experimental Details

A. Crystal Data

Empirical Formula	$C_{24}H_{22}N_2O_8S_2$
Formula Weight	530.57
Crystal Color, Habit	yellow, platelet
Crystal Dimensions	0.160 × 0.100 × 0.030 mm
Crystal System	orthorhombic
Lattice Type	Primitive
Lattice Parameters	a = 5.01471(10) Å<br>b = 18.6873(3) Å<br>c = 25.4365(6) Å<br>V = 2383.70(8) $Å^3$
Space Group	$P2_12_12_1$ (#19)
Z value	4
D_{calc}	1.478 g/cm^3
F_{000}	1104.00
μ(MoKα)	2.771 cm^{-1}

B. Intensity Measurements

Diffractometer	R-AXIS RAPID
Radiation<br>Voltage, Current	MoKα (λ = 0.71075 Å)<br>50kV, 24mA
Temperature	−150.0 °C
Detector Aperture	460.0 × 256.0 mm
Data Images	111 exposures
ω oscillation Range (χ = 45.0, ϕ = 0.0)	130.0–190.0°
Exposure Rate	40.0 s/°
ω oscillation Range (χ = 45.0, ϕ = 180.0)	0.0–162.0°
Exposure Rate	40.0 s/°
Detector Position	127.40 mm
Pixel Size	0.100 mm
$2\theta_{max}$	55.0°
No. of Reflections Measured	Total: 23904<br>Unique: 5463 (R_{int} = 0.0368)<br>Friedel pairs: 2288
Corrections	Lorentz-polarization<br>Absorption<br>(trans. factors: 0.823–0.992)

C. Structure Solution and Refinement

Structure Solution	Direct Methods (SIR2008)
Refinement	Full-matrix least-squares on F^2
Function Minimized	Σ w $(Fo^2 - Fc^2)^2$
Least Squares Weights	$w = 1/[\sigma^2(Fo^2) + (0.0544 \cdot P)^2 + 0.4090 \cdot P]$<br>where $P = (Max(Fo^2,0) + 2Fc^2)/3$
$2\theta_{max}$ cutoff	55.0°
Anomalous Dispersion	All non-hydrogen atoms
No. Observations (All reflections)	5463
No. Variables	335
Reflection/Parameter Ratio	16.31
Residuals: R1 ($I>2.00\sigma(I)$)	0.0362
Residuals: R (All reflections)	0.0401
Residuals: wR2 (All reflections)	0.0925
Goodness of Fit Indicator	1.081
Flack Parameter (Friedel pairs = 2288)	−0.00(6)
Max Shift/Error in Final Cycle	0.001
Maximum peak in Final Diff. Map	0.43 $e^-/Å^3$
Minimum peak in Final Diff. Map	−0.25 $e^-/Å^3$

Table 1. Atomic coordinates and B_{iso}/B_{eq}.

Atom	x	y	z	B_{eq}
S1	0.72615(12)	0.45508(3)	0.74252(2)	2.955(11)
S2	0.11009(11)	0.76088(3)	0.61998(2)	2.465(10)
O1	0.5950(4)	0.41462(8)	0.34165(5)	3.04(3)
O2	0.8653(3)	0.51705(6)	0.52992(5)	1.72(2)
O3	1.3628(3)	0.40269(10)	0.57261(6)	2.86(3)
O4	0.8638(4)	0.28464(7)	0.67573(6)	2.89(3)
O5	1.2260(3)	0.33814(8)	0.70915(6)	2.61(3)
O6	0.7103(3)	0.66903(7)	0.52069(5)	2.02(2)
O7	0.1614(3)	0.56930(7)	0.65940(5)	2.36(3)
O8	0.3970(3)	0.64610(7)	0.70896(5)	2.50(3)
N1	0.9272(3)	0.39175(8)	0.59414(6)	1.42(2)
N2	0.3927(3)	0.61475(8)	0.57017(6)	1.69(2)
C1	0.6300(4)	0.43285(10)	0.38704(7)	1.94(3)
C2	0.8268(4)	0.39436(9)	0.42089(7)	1.65(3)
C3	0.9540(4)	0.33419(10)	0.40047(8)	2.18(3)
C4	1.1399(5)	0.29758(10)	0.43053(8)	2.36(3)
C5	1.1909(4)	0.31988(10)	0.48145(8)	2.02(3)
C6	1.0606(4)	0.37888(9)	0.50325(7)	1.53(3)
C7	0.8819(4)	0.41809(9)	0.47201(7)	1.39(3)
C8	0.7736(4)	0.48843(9)	0.49073(6)	1.33(3)
C9	0.5579(4)	0.52272(9)	0.45912(6)	1.37(3)
C10	0.4304(4)	0.58437(9)	0.47781(7)	1.55(3)
C11	0.2157(4)	0.61272(10)	0.44981(7)	1.86(3)
C12	0.1394(4)	0.58322(10)	0.40194(8)	2.11(3)
C13	0.2758(4)	0.52540(10)	0.38155(7)	1.96(3)
C14	0.4829(4)	0.49434(10)	0.41026(7)	1.64(3)
C15	1.1287(4)	0.39458(9)	0.55965(7)	1.57(3)
C16	0.9681(4)	0.40581(9)	0.65013(7)	1.48(3)
C17	0.7230(4)	0.44389(10)	0.67185(7)	1.92(3)
C18	1.0102(4)	0.33530(10)	0.67921(7)	1.80(3)
C19	1.2760(5)	0.27564(15)	0.74116(10)	3.64(5)
C20	0.5274(4)	0.62575(9)	0.52513(7)	1.58(3)
C21	0.4806(4)	0.64832(9)	0.61868(7)	1.70(3)
C22	0.4516(4)	0.72966(9)	0.61812(8)	1.89(3)
C23	0.3275(4)	0.61577(9)	0.66404(7)	1.74(3)
C24	0.2502(6)	0.62259(11)	0.75493(8)	2.99(4)

$B_{eq} = 8/3\pi^2(U_{11}(aa^*)^2 + U_{22}(bb^*)^2 + U_{33}(cc^*)^2 + 2U_{12}(aa^*bb^*)\cos\gamma + 2U_{13}(aa^*cc^*)\cos\beta + 2U_{23}(bb^*cc^*)\cos\alpha)$.

Table 2. Atomic coordinates and B_{iso} involving hydrogen atoms.

Atom	x	y	z	B_{iso}
H1	0.88193	0.49110	0.74706	4.564
H2	0.03550	0.74079	0.58269	3.715
H3	0.76613	0.38116	0.58283	1.709
H4	0.25072	0.58708	0.57024	2.028
H5	0.91339	0.31825	0.36592	2.613
H6	1.23158	0.25752	0.41630	2.828
H7	1.31750	0.29446	0.50203	2.428
H8	0.12084	0.65253	0.46358	2.232
H9	−0.00704	0.60294	0.38320	2.536
H10	0.22886	0.50678	0.34805	2.353
H11	1.12845	0.43696	0.65482	1.776
H12	0.56210	0.41633	0.66189	2.307
H13	0.70933	0.49163	0.65520	2.307
H14	1.14569	0.27370	0.76983	4.371
H15	1.45638	0.27836	0.75587	4.371
H16	1.25995	0.23250	0.71948	4.371
H17	0.67370	0.63678	0.62365	2.037
H18	0.53787	0.74852	0.58598	2.265
H19	0.54800	0.74958	0.64876	2.265
H20	0.31375	0.57521	0.76568	3.593
H21	0.05972	0.61999	0.74650	3.593
H22	0.27776	0.65668	0.78370	3.593

Table 3. Anisotropic displacement parameters

Atom	U_{11}	U_{22}	U_{33}	U_{12}	U_{13}	U_{23}
S1	0.0331(3)	0.0502(3)	0.0290(3)	0.0013(3)	0.0015(2)	−0.0137(2)
S2	0.0266(2)	0.0316(2)	0.0355(3)	0.0061(2)	0.0002(2)	−0.0019(2)
O1	0.0499(10)	0.0462(9)	0.0194(7)	0.0066(8)	−0.0103(7)	−0.0087(6)
O2	0.0254(7)	0.0223(6)	0.0176(6)	−0.0003(6)	−0.0039(5)	0.0001(5)
O3	0.0113(6)	0.0662(10)	0.0313(8)	−0.0009(7)	−0.0021(6)	−0.0057(7)
O4	0.0528(9)	0.0235(7)	0.0335(8)	−0.0100(7)	−0.0081(7)	0.0068(5)
O5	0.0225(7)	0.0441(8)	0.0326(7)	0.0027(7)	−0.0057(6)	0.0197(6)
O6	0.0255(7)	0.0238(6)	0.0274(7)	−0.0042(6)	0.0061(6)	0.0002(5)
O7	0.0371(8)	0.0268(7)	0.0256(7)	−0.0127(6)	0.0068(6)	−0.0015(5)
O8	0.0427(9)	0.0326(7)	0.0199(6)	−0.0140(7)	0.0032(6)	−0.0021(5)
N1	0.0122(7)	0.0232(7)	0.0187(7)	−0.0024(6)	−0.0020(6)	0.0038(6)
N2	0.0198(7)	0.0219(7)	0.0225(7)	−0.0041(7)	0.0035(6)	−0.0016(6)
C1	0.0264(9)	0.0288(9)	0.0186(8)	−0.0040(8)	−0.0010(8)	−0.0006(7)
C2	0.0207(9)	0.0221(8)	0.0198(8)	−0.0034(7)	0.0022(7)	−0.0003(7)
C3	0.0343(11)	0.0288(10)	0.0197(9)	−0.0035(9)	0.0056(8)	−0.0057(7)
C4	0.0333(11)	0.0239(9)	0.0323(10)	0.0041(9)	0.0083(9)	−0.0047(7)
C5	0.0226(10)	0.0242(9)	0.0301(10)	0.0037(7)	0.0029(8)	0.0034(7)
C6	0.0134(8)	0.0216(8)	0.0233(9)	−0.0019(7)	0.0028(6)	0.0015(7)
C7	0.0144(8)	0.0213(8)	0.0172(8)	−0.0027(7)	0.0016(7)	0.0010(6)
C8	0.0156(8)	0.0196(8)	0.0153(7)	−0.0020(7)	−0.0006(6)	0.0025(6)
C9	0.0149(8)	0.0204(8)	0.0168(8)	−0.0016(7)	0.0012(6)	0.0043(6)
C10	0.0170(8)	0.0225(8)	0.0194(8)	−0.0020(7)	0.0036(7)	0.0049(7)
C11	0.0187(9)	0.0255(9)	0.0266(9)	0.0021(8)	0.0038(7)	0.0078(7)
C12	0.0195(9)	0.0313(10)	0.0295(10)	−0.0024(8)	−0.0032(8)	0.0129(8)
C13	0.0231(9)	0.0307(9)	0.0207(8)	−0.0057(8)	−0.0055(8)	0.0052(7)
C14	0.0200(9)	0.0246(9)	0.0179(8)	−0.0052(7)	−0.0013(7)	0.0030(7)
C15	0.0144(8)	0.0209(8)	0.0244(9)	0.0006(7)	−0.0020(7)	0.0028(6)
C16	0.0154(8)	0.0200(8)	0.0208(8)	−0.0004(7)	−0.0017(7)	0.0024(6)
C17	0.0193(9)	0.0289(9)	0.0248(9)	0.0024(8)	−0.0017(8)	−0.0021(7)
C18	0.0240(9)	0.0265(9)	0.0179(8)	0.0039(8)	0.0008(7)	0.0013(7)
C19	0.0372(12)	0.0612(15)	0.0399(12)	0.0121(12)	−0.0009(11)	0.0319(11)
C20	0.0196(8)	0.0188(8)	0.0219(9)	0.0035(7)	0.0029(7)	0.0036(7)
C21	0.0210(9)	0.0220(8)	0.0215(8)	−0.0021(7)	0.0037(7)	−0.0001(7)
C22	0.0234(9)	0.0205(9)	0.0278(9)	−0.0019(7)	0.0033(8)	−0.0001(7)
C23	0.0251(10)	0.0192(8)	0.0219(9)	−0.0015(7)	0.0031(7)	0.0003(7)
C24	0.0578(14)	0.0354(11)	0.0206(9)	−0.0092(11)	0.0083(10)	−0.0014(8)

The general temperature factor expression: $\exp(-2\pi^2(a^{*2}U_{11}h^2 + b^{*2}U_{22}k^2 + c^{*2}U_{33}l^2 + 2a^*b^*U_{12}hk + 2a^*c^*U_{13}hl + 2b^*c^*U_{23}kl))$.

Table 4. Bond lengths (Å).

Atom	Atom	Distance	Atom	Atom	Distance
S1	C17	1.8098(19)	S2	C22	1.810(2)
O1	C1	1.217(2)	O2	C8	1.221(2)
O3	C15	1.229(2)	O4	C18	1.201(2)
O5	C18	1.325(2)	O5	C19	1.446(3)
O6	C20	1.228(2)	O7	C23	1.209(2)
O8	C23	1.322(2)	O8	C24	1.450(3)
N1	C15	1.339(2)	N1	C16	1.463(2)
N2	C20	1.346(2)	N2	C21	1.453(2)
C1	C2	1.494(3)	C1	C14	1.488(3)
C2	C3	1.393(3)	C2	C7	1.401(3)
C3	C4	1.386(3)	C4	C5	1.384(3)
C5	C6	1.397(3)	C6	C7	1.404(2)
C6	C15	1.504(3)	C7	C8	1.500(2)
C8	C9	1.492(2)	C9	C10	1.401(2)
C9	C14	1.403(2)	C10	C11	1.395(3)
C10	C20	1.511(2)	C11	C12	1.390(3)
C12	C13	1.380(3)	C13	C14	1.396(3)
C16	C17	1.524(3)	C16	C18	1.526(3)
C21	C22	1.527(2)	C21	C23	1.514(3)

Table 5. Bond lengths involving hydrogens (Å).

Atom	Atom	Distance	Atom	Atom	Distance
S1	H1	1.038	S2	H2	1.087
N1	H3	0.880	N2	H4	0.880
C3	H5	0.950	C4	H6	0.950
C5	H7	0.950	C11	H8	0.950
C12	H9	0.950	C13	H10	0.950
C16	H11	1.000	C17	H12	0.990
C17	H13	0.990	C19	H14	0.980
C19	H15	0.980	C19	H16	0.980
C21	H17	1.000	C22	H18	0.990
C22	H19	0.990	C24	H20	0.980
C24	H21	0.980	C24	H22	0.980

Table 6. Bond angles (°).

Atom	Atom	Atom	Angle	Atom	Atom	Atom	Angle
C18	O5	C19	115.68(17)	C23	O8	C24	115.66(16)
C15	N1	C16	121.67(15)	C20	N2	C21	120.32(15)
O1	C1	C2	120.50(18)	O1	C1	C14	121.42(18)
C2	C1	C14	118.07(15)	C1	C2	C3	118.43(16)
C1	C2	C7	120.84(16)	C3	C2	C7	120.73(17)
C2	C3	C4	120.04(18)	C3	C4	C5	119.45(18)
C4	C5	C6	121.51(18)	C5	C6	C7	119.08(17)
C5	C6	C15	115.24(16)	C7	C6	C15	125.68(15)
C2	C7	C6	119.07(16)	C2	C7	C8	120.04(15)
C6	C7	C8	120.58(15)	O2	C8	C7	120.44(16)
O2	C8	C9	121.65(15)	C7	C8	C9	117.88(14)
C8	C9	C10	120.07(14)	C8	C9	C14	120.62(15)
C10	C9	C14	119.30(16)	C9	C10	C11	119.42(16)
C9	C10	C20	122.98(16)	C11	C10	C20	117.43(15)
C10	C11	C12	120.58(17)	C11	C12	C13	120.22(18)
C12	C13	C14	119.84(17)	C1	C14	C9	120.71(16)
C1	C14	C13	118.84(16)	C9	C14	C13	120.39(17)
O3	C15	N1	123.37(17)	O3	C15	C6	119.81(16)
N1	C15	C6	116.48(15)	N1	C16	C17	108.90(14)
N1	C16	C18	109.65(14)	C17	C16	C18	109.82(15)
S1	C17	C16	114.01(13)	O4	C18	O5	124.98(18)
O4	C18	C16	124.08(17)	O5	C18	C16	110.93(16)
O6	C20	N2	123.62(16)	O6	C20	C10	120.28(16)
N2	C20	C10	116.00(15)	N2	C21	C22	113.14(15)
N2	C21	C23	108.66(14)	C22	C21	C23	111.02(15)
S2	C22	C21	114.27(13)	O7	C23	O8	125.02(17)
O7	C23	C21	124.31(16)	O8	C23	C21	110.65(15)

Table 7. Bond angles involving hydrogens (°).

Atom	Atom	Atom	Angle	Atom	Atom	Atom	Angle
C17	S1	H1	101.1	C22	S2	H2	101.0
C15	N1	H3	119.2	C16	N1	H3	119.2
C20	N2	H4	119.8	C21	N2	H4	119.8
C2	C3	H5	120.0	C4	C3	H5	120.0
C3	C4	H6	120.3	C5	C4	H6	120.3
C4	C5	H7	119.2	C6	C5	H7	119.3
C10	C11	H8	119.7	C12	C11	H8	119.7
C11	C12	H9	119.9	C13	C12	H9	119.9
C12	C13	H10	120.1	C14	C13	H10	120.1
N1	C16	H11	109.5	C17	C16	H11	109.5
C18	C16	H11	109.5	S1	C17	H12	108.8
S1	C17	H13	108.7	C16	C17	H12	108.8
C16	C17	H13	108.7	H12	C17	H13	107.6
O5	C19	H14	109.5	O5	C19	H15	109.5
O5	C19	H16	109.5	H14	C19	H15	109.5
H14	C19	H16	109.5	H15	C19	H16	109.5
N2	C21	H17	107.9	C22	C21	H17	108.0
C23	C21	H17	108.0	S2	C22	H18	108.7
S2	C22	H19	108.7	C21	C22	H18	108.7
C21	C22	H19	108.7	H18	C22	H19	107.6
O8	C24	H20	109.5	O8	C24	H21	109.5
O8	C24	H22	109.5	H20	C24	H21	109.5
H20	C24	H22	109.4	H21	C24	H22	109.5

Table 8. Torsion Angles (°). (Those having bond angles >160 or <20 degrees are excluded.)

Atom1	Atom2	Atom3	Atom4	Angle	Atom1	Atom2	Atom3	Atom4	Angle
C19	O5	C18	O4	−2.8(3)	C19	O5	C18	C16	176.24(15)
C24	O8	C23	O7	−2.8(3)	C24	O8	C23	C21	176.01(14)
C15	N1	C16	C17	144.75(14)	C15	N1	C16	C18	−95.06(17)
C16	N1	C15	O3	6.6(3)	C16	N1	C15	C6	179.87(13)
C20	N2	C21	C22	67.0(2)	C20	N2	C21	C23	−169.24(14)
C21	N2	C20	O6	−7.6(3)	C21	N2	C20	C10	175.97(13)
O1	C1	C2	C3	−4.6(3)	O1	C1	C2	C7	175.53(16)
O1	C1	C14	C9	−168.64(17)	O1	C1	C14	C13	8.6(3)
C2	C1	C14	C9	10.4(2)	C2	C1	C14	C13	−172.31(14)
C14	C1	C2	C3	176.32(14)	C14	C1	C2	C7	−3.6(2)
C1	C2	C3	C4	179.30(15)	C1	C2	C7	C6	177.62(14)
C1	C2	C7	C8	−8.8(2)	C3	C2	C7	C6	−2.3(3)
C3	C2	C7	C8	171.35(15)	C7	C2	C3	C4	−0.8(3)
C2	C3	C4	C5	2.2(3)	C3	C4	C5	C6	−0.4(3)
C4	C5	C6	C7	−2.6(3)	C4	C5	C6	C15	177.54(16)
C5	C6	C7	C2	3.9(2)	C5	C6	C7	C8	−169.65(15)
C5	C6	C15	O3	54.5(2)	C5	C6	C15	N1	−118.96(17)
C7	C6	C15	O3	−125.29(18)	C7	C6	C15	N1	61.2(2)
C15	C6	C7	C2	−176.26(14)	C15	C6	C7	C8	10.2(3)
C2	C7	C8	O2	−163.78(15)	C2	C7	C8	C9	14.4(2)
C6	C7	C8	O2	9.7(2)	C6	C7	C8	C9	−172.05(14)
O2	C8	C9	C10	−8.6(2)	O2	C8	C9	C14	170.54(14)
C7	C8	C9	C10	173.18(13)	C7	C8	C9	C14	−7.6(2)
C8	C9	C10	C11	−175.19(14)	C8	C9	C10	C20	9.7(2)
C8	C9	C14	C1	−4.6(2)	C8	C9	C14	C13	178.15(14)
C10	C9	C14	C1	174.54(14)	C10	C9	C14	C13	−2.7(2)
C14	C9	C10	C11	5.6(2)	C14	C9	C10	C20	−169.47(14)
C9	C10	C11	C12	−4.4(3)	C9	C10	C20	O6	81.7(2)
C9	C10	C20	N2	−101.77(19)	C11	C10	C20	O6	−93.5(2)
C11	C10	C20	N2	83.04(19)	C20	C10	C11	C12	171.02(14)
C10	C11	C12	C13	−0.0(3)	C11	C12	C13	C14	3.0(3)
C12	C13	C14	C1	−178.94(15)	C12	C13	C14	C9	−1.7(3)
N1	C16	C17	S1	172.77(11)	N1	C16	C18	O4	−51.3(2)
N1	C16	C18	O5	129.66(14)	C17	C16	C18	O4	68.3(2)
C17	C16	C18	O5	−110.71(16)	C18	C16	C17	S1	52.68(18)
N2	C21	C22	S2	69.97(18)	N2	C21	C23	O7	0.3(2)
N2	C21	C23	O8	−178.51(13)	C22	C21	C23	O7	125.36(17)
C22	C21	C23	O8	−53.47(19)	C23	C21	C22	S2	−52.51(19)

Table 9. Possible hydrogen bonds.

Donor	H	Acceptor	D...A	D-H	H...A	D-H...A
N1	H3	O3 [1]	2.890(2)	0.88	2.08	152.94
N2	H4	O7	2.687(2)	0.88	2.34	103.93

Symmetry Operators: (1) X−1,Y,Z.

Table 10. Intramolecular contacts less than 3.60 Å.

Atom	Atom	Distance	Atom	Atom	Distance
S1	O5	3.4320(16)	S1	C18	3.1035(19)
S2	O8	3.4342(15)	S2	N2	3.3273(16)
S2	C23	3.1301(18)	O1	C3	2.782(3)
O1	C13	2.807(2)	O2	O3	3.460(2)
O2	O6	2.9540(17)	O2	N1	2.8718(19)
O2	N2	3.162(2)	O2	C6	2.843(2)
O2	C10	2.845(2)	O2	C15	2.748(2)
O2	C20	2.648(2)	O3	C5	2.918(3)
O3	C7	3.528(2)	O3	C16	2.795(2)
O3	C18	3.474(2)	O4	N1	2.901(2)
O4	C17	3.060(2)	O4	C19	2.659(3)
O5	N1	3.436(2)	O5	C17	3.342(2)
O6	C8	3.474(2)	O6	C9	3.242(2)
O6	C11	3.242(2)	O6	C21	2.773(2)
O6	C22	3.018(2)	O7	N2	2.687(2)
O7	C22	3.493(2)	O7	C24	2.664(2)
O8	N2	3.579(2)	O8	C22	2.802(2)
N1	C5	3.431(3)	N1	C7	3.154(2)
N1	C8	3.283(2)	N2	C9	3.409(2)
N2	C11	3.188(2)	C1	C8	2.925(2)
C2	C5	2.765(3)	C2	C9	2.919(2)
C3	C6	2.796(3)	C4	C7	2.803(3)
C7	C14	2.916(3)	C8	C15	3.053(2)
C8	C20	2.979(2)	C9	C12	2.792(3)
C10	C13	2.795(3)	C11	C14	2.775(3)
C15	C18	3.291(3)	C20	C22	3.084(3)

Table 11. Intramolecular contacts less than 3.60 Å involving hydrogens.

Atom	Atom	Distance	Atom	Atom	Distance
S1	H11	3.027	S1	H20	3.109
S2	H4	3.556	O1	H5	2.485
O1	H10	2.523	O2	H3	2.917
O2	H4	3.502	O2	H13	3.316
O2	H17	3.408	O3	H3	3.030
O3	H7	2.714	O3	H11	2.483
O4	H3	3.013	O4	H11	3.185
O4	H12	2.910	O4	H14	2.787
O4	H16	2.477	O5	H1	3.475
O5	H11	2.358	O6	H4	3.041
O6	H8	3.308	O6	H17	2.694
O6	H18	2.390	O7	H4	2.335
O7	H12	3.495	O7	H13	3.109
O7	H17	3.003	O7	H20	2.811
O7	H21	2.463	O8	H13	3.557
O8	H17	2.582	O8	H19	2.580
N1	H7	3.553	N1	H12	2.556
N1	H13	2.663	N2	H2	2.976
N2	H8	3.116	N2	H13	3.534
N2	H18	2.634	N2	H19	3.309
C1	H5	2.626	C1	H10	2.634
C2	H6	3.267	C3	H7	3.248
C5	H3	3.535	C5	H5	3.252
C6	H3	2.506	C6	H6	3.282
C7	H3	2.960	C7	H5	3.285
C7	H7	3.270	C8	H3	3.084
C9	H4	3.436	C9	H8	3.271
C9	H10	3.285	C10	H4	2.518
C10	H9	3.275	C11	H4	3.106
C11	H10	3.259	C13	H8	3.256
C14	H9	3.260	C15	H7	2.558
C15	H11	2.547	C16	H1	2.967
C17	H3	2.559	C18	H1	3.445
C18	H3	2.871	C18	H12	2.745
C18	H13	3.344	C18	H14	2.665
C18	H15	3.153	C18	H16	2.512
C20	H2	3.585	C20	H8	2.619
C20	H17	2.619	C20	H18	2.768
C21	H2	2.968	C21	H13	3.279
C22	H4	3.098	C23	H2	3.447
C23	H4	2.476	C23	H13	3.016
C23	H18	3.348	C23	H19	2.762
C23	H20	2.695	C23	H21	2.492
C23	H22	3.148	H1	H11	2.838

Table 11. *Cont.*

Atom	Atom	Distance	Atom	Atom	Distance
H1	H12	3.036	H1	H13	2.492
H1	H20	3.288	H2	H4	3.085
H2	H8	3.476	H2	H18	2.525
H2	H19	3.075	H3	H11	2.782
H3	H12	2.350	H3	H13	2.781
H4	H8	3.046	H4	H17	2.685
H4	H18	3.367	H5	H6	2.340
H6	H7	2.328	H8	H9	2.335
H9	H10	2.330	H11	H12	2.872
H11	H13	2.337	H13	H17	2.834
H17	H18	2.396	H17	H19	2.291

Table 12. Intermolecular contacts less than 3.60 Å.

Atom	aTom	Distance	Atom	Atom	Distance
S1	O5[1]	3.4332(16)	S2	C11[2]	3.5555(19)
S2	C22[1]	3.354(2)	O1	C24[3]	2.889(3)
O2	O3[1]	3.478(2)	O2	N2[4]	3.373(2)
O2	C10[4]	3.372(2)	O2	C11[4]	3.230(2)
O3	O2[4]	3.478(2)	O3	N1[4]	2.890(2)
O3	C8[4]	3.339(2)	O3	C17[4]	3.198(2)
O4	O5[1]	3.457(2)	O4	C3[5]	3.593(3)
O4	C4[5]	3.305(3)	O4	C19[1]	3.389(3)
O4	C24[6]	3.551(2)	O5	S1[4]	3.4332(16)
O5	O4[4]	3.457(2)	O5	C17[4]	3.319(2)
O6	C11[4]	3.284(2)	O7	C16[1]	3.214(2)
O7	C17[1]	3.229(2)	O8	C19[7]	3.187(3)
N1	O3[1]	2.890(2)	N2	O2[1]	3.373(2)
C2	C13[4]	3.474(3)	C3	O4[8]	3.593(3)
C4	O4[8]	3.305(3)	C8	O3[1]	3.339(2)
C8	C11[4]	3.375(3)	C8	C12[4]	3.406(3)
C9	C12[4]	3.450(3)	C10	O2[1]	3.372(2)
C11	S2[9]	3.5555(19)	C11	O2[1]	3.230(2)
C11	O6[1]	3.284(2)	C11	C8[1]	3.375(3)
C12	C8[1]	3.406(3)	C12	C9[1]	3.450(3)
C13	C2[1]	3.474(3)	C16	O7[4]	3.214(2)
C17	O3[1]	3.198(2)	C17	O5[1]	3.319(2)
C17	O7[4]	3.229(2)	C19	O4[4]	3.389(3)
C19	O8[10]	3.187(3)	C22	S2[4]	3.354(2)
C24	O1[11]	2.889(3)	C24	O4[12]	3.551(2)

Symmetry Operators:

(1) X−1,Y,Z
(2) X+1/2−1,−Y+1/2+1,−Z+1
(3) −X+1/2,−Y+1,Z+1/2−1
(4) X+1,Y,Z
(5) X+1/2−1,−Y+1/2,−Z+1
(6) −X+1,Y+1/2−1,−Z+1/2+1
(7) −X+2,Y+1/2,−Z+1/2+1
(8) X+1/2,−Y+1/2,−Z+1
(9) X+1/2,−Y+1/2+1,−Z+1
(10) −X+2,Y+1/2−1,−Z+1/2+1
(11) −X+1/2,−Y+1,Z+1/2
(12) −X+1,Y+1/2,−Z+1/2+1

Table 13. Intermolecular contacts less than 3.60 Å involving hydrogens.

Atom	Atom	Distance	Atom	Atom	Distance
S1	H10^{1}	3.594	S1	H15^{2}	3.585
S1	H21^{3}	3.508	S2	H9^{4}	3.189
S2	H14^{5}	3.092	S2	H17^{2}	3.190
S2	H18^{2}	3.006	S2	H19^{2}	2.920
O1	H1^{6}	2.984	O1	H16^{7}	3.265
O1	H20^{8}	2.823	O1	H20^{6}	3.544
O1	H21^{8}	2.623	O1	H22^{8}	2.728
O2	H4^{3}	2.549	O2	H8^{3}	3.301
O3	H3^{3}	2.078	O3	H4^{3}	3.492
O3	H6^{9}	3.530	O3	H12^{3}	2.494
O3	H13^{3}	3.193	O4	H5^{7}	3.150
O4	H5^{9}	3.524	O4	H6^{7}	2.557
O4	H15^{2}	2.888	O4	H16^{2}	3.370
O4	H22^{10}	2.699	O5	H12^{3}	2.534
O6	H2^{3}	2.635	O6	H2^{4}	3.244
O6	H4^{3}	3.358	O6	H8^{3}	2.539
O6	H8^{4}	3.388	O6	H18^{4}	3.526
O7	H1^{2}	3.012	O7	H11^{2}	2.481
O7	H13^{2}	2.694	O7	H17^{2}	2.898
O8	H14^{11}	3.352	O8	H15^{11}	2.729
O8	H16^{11}	2.980	O8	H21^{3}	3.492
N1	H6^{7}	2.969	C1	H10^{3}	3.451
C2	H10^{3}	3.451	C3	H7^{7}	3.521
C3	H16^{7}	3.437	C3	H22^{6}	3.265
C4	H3^{9}	3.417	C4	H7^{7}	2.918
C5	H7^{7}	2.872	C6	H7^{7}	3.464
C8	H8^{3}	3.593	C9	H9^{3}	3.277
C11	H2^{4}	3.279	C11	H18^{12}	2.889
C12	H18^{12}	3.200	C12	H19^{12}	3.411
C13	H20^{8}	3.525	C14	H9^{3}	3.337
C15	H3^{3}	3.260	C15	H6^{7}	3.524
C15	H12^{3}	3.414	C16	H12^{3}	3.000
C17	H11^{2}	3.015	C18	H6^{7}	3.296
C18	H12^{3}	3.186	C18	H15^{2}	3.556
C19	H5^{9}	3.312	C19	H19^{13}	2.976
C19	H21^{10}	3.375	C19	H22^{13}	3.217
C20	H8^{3}	3.400	C21	H2^{3}	3.401
C22	H2^{3}	3.071	C22	H8^{4}	3.144
C22	H9^{4}	3.135	C22	H14^{11}	3.589
C22	H15^{11}	3.364	C23	H11^{2}	3.495
C23	H17^{2}	3.458	C24	H1^{2}	3.080
C24	H5^{14}	3.470	C24	H10^{1}	3.386
C24	H14^{5}	3.509	C24	H15^{11}	3.273
C24	H16^{5}	3.345	C24	H16^{11}	3.268

Table 13. *Cont.*

Atom	Atom	Distance	Atom	Atom	Distance
H1	O1^{14}	2.984	H1	O7^{3}	3.012
H1	C24^{3}	3.080	H1	H10^{14}	3.226
H1	H20^{3}	2.717	H1	H21^{3}	2.568
H2	O6^{2}	2.635	H2	O6^{12}	3.244
H2	C11^{12}	3.279	H2	C21^{2}	3.401
H2	C22^{2}	3.071	H2	H8^{12}	3.112
H2	H17^{2}	2.856	H2	H18^{2}	2.501
H2	H19^{2}	2.971	H3	O3^{2}	2.078
H3	C4^{7}	3.417	H3	C15^{2}	3.260
H3	H6^{7}	2.597	H3	H7^{2}	3.451
H4	O2^{2}	2.549	H4	O3^{2}	3.492
H4	O6^{2}	3.358	H4	H11^{2}	3.588
H4	H17^{2}	3.329	H5	O4^{7}	3.524
H5	O4^{9}	3.150	H5	C19^{7}	3.312
H5	C24^{6}	3.470	H5	H15^{7}	3.592
H5	H16^{7}	2.492	H5	H20^{6}	3.512
H5	H22^{6}	2.644	H6	O3^{7}	3.530
H6	O4^{9}	2.557	H6	N1^{9}	2.969
H6	C15^{9}	3.524	H6	C18^{9}	3.296
H6	H3^{9}	2.597	H6	H7^{7}	3.094
H7	C3^{9}	3.521	H7	C4^{9}	2.918
H7	C5^{9}	2.872	H7	C6^{9}	3.464
H7	H3^{3}	3.451	H7	H6^{9}	3.094
H7	H7^{7}	3.010	H7	H7^{9}	3.010
H8	O2^{2}	3.301	H8	O6^{2}	2.539
H8	O6^{12}	3.388	H8	C8^{2}	3.593
H8	C20^{2}	3.400	H8	C22^{12}	3.144
H8	H2^{4}	3.112	H8	H18^{12}	2.276
H8	H19^{12}	3.413	H9	S2^{12}	3.189
H9	C9^{2}	3.277	H9	C14^{2}	3.337
H9	C22^{12}	3.135	H9	H18^{12}	2.893
H9	H19^{12}	2.887	H10	S1^{8}	3.594
H10	C1^{2}	3.451	H10	C2^{2}	3.451
H10	C24^{8}	3.386	H10	H1^{6}	3.226
H10	H20^{8}	2.604	H10	H22^{8}	3.466
H11	O7^{3}	2.481	H11	C17^{3}	3.015
H11	C23^{3}	3.495	H11	H4^{3}	3.588
H11	H12^{3}	2.216	H11	H13^{3}	3.087
H12	O3^{2}	2.494	H12	O5^{2}	2.534
H12	C15^{2}	3.414	H12	C16^{2}	3.000
H12	C18^{2}	3.186	H12	H11^{2}	2.216
H12	H15^{2}	3.556	H13	O3^{2}	3.193
H13	O7^{3}	2.694	H13	H11^{2}	3.087

Table 13. *Cont.*

Atom	Atom	Distance	Atom	Atom	Distance
H14	$S2^{10}$	3.092	H14	$O8^{13}$	3.352
H14	$C22^{13}$	3.589	H14	$C24^{10}$	3.509
H14	$H15^{2}$	3.476	H14	$H19^{13}$	2.617
H14	$H21^{10}$	3.080	H14	$H22^{10}$	3.338
H15	$S1^{3}$	3.585	H15	$O4^{3}$	2.888
H15	$O8^{13}$	2.729	H15	$C18^{3}$	3.556
H15	$C22^{13}$	3.364	H15	$C24^{13}$	3.273
H15	$H5^{9}$	3.592	H15	$H12^{3}$	3.556
H15	$H14^{3}$	3.476	H15	$H19^{13}$	2.485
H15	$H22^{13}$	2.822	H16	$O1^{9}$	3.265
H16	$O4^{3}$	3.370	H16	$O8^{13}$	2.980
H16	$C3^{9}$	3.437	H16	$C24^{10}$	3.345
H16	$C24^{13}$	3.268	H16	$H5^{9}$	2.492
H16	$H19^{13}$	3.502	H16	$H21^{10}$	2.782
H16	$H22^{10}$	3.047	H16	$H22^{13}$	2.718
H17	$S2^{3}$	3.190	H17	$O7^{3}$	2.898
H17	$C23^{3}$	3.458	H17	$H2^{3}$	2.856
H17	$H4^{3}$	3.329	H18	$S2^{3}$	3.006
H18	$O6^{12}$	3.526	H18	$C11^{4}$	2.889
H18	$C12^{4}$	3.200	H18	$H2^{3}$	2.501
H18	$H8^{4}$	2.276	H18	$H9^{4}$	2.893
H19	$S2^{3}$	2.920	H19	$C12^{4}$	3.411
H19	$C19^{11}$	2.976	H19	$H2^{3}$	2.971
H19	$H8^{4}$	3.413	H19	$H9^{4}$	2.887
H19	$H14^{11}$	2.617	H19	$H15^{11}$	2.485
H19	$H16^{11}$	3.502	H20	$O1^{1}$	2.823
H20	$O1^{14}$	3.544	H20	$C13^{1}$	3.525
H20	$H1^{2}$	2.717	H20	$H5^{14}$	3.512
H20	$H10^{1}$	2.604	H21	$S1^{2}$	3.508
H21	$O1^{1}$	2.623	H21	$O8^{2}$	3.492
H21	$C19^{5}$	3.375	H21	$H1^{2}$	2.568
H21	$H14^{5}$	3.080	H21	$H16^{5}$	2.782
H22	$O1^{1}$	2.728	H22	$O4^{5}$	2.699
H22	$C3^{14}$	3.265	H22	$C19^{11}$	3.217
H22	$H5^{14}$	2.644	H22	$H10^{1}$	3.466
H22	$H14^{5}$	3.338	H22	$H15^{11}$	2.822
H22	$H16^{5}$	3.047	H22	$H16^{11}$	2.718

Symmetry Operators:

(1) −X+1/2,−Y+1,Z+1/2
(2) X−1,Y,Z
(3) X+1,Y,Z
(4) X+1/2,−Y+1/2+1,−Z+1
(5) −X+1,Y+1/2,−Z+1/2+1
(6) −X+1/2+1,−Y+1,Z+1/2−1
(7) X+1/2−1,−Y+1/2,−Z+1
(8) −X+1/2,−Y+1,Z+1/2−1
(9) X+1/2,−Y+1/2,−Z+1
(10) −X+1,Y+1/2−1,−Z+1/2+1
(11) −X+2,Y+1/2,−Z+1/2+1
(12) X+1/2−1,−Y+1/2+1,−Z+1
(13) −X+2,Y+1/2−1,−Z+1/2+1
(14) −X+1/2+1,−Y+1,Z+1/2
