# Supplementary material for: Synthesis of a Novel Cysteine-Incorporated Anthraquinone Derivative and Its Structural Properties
Source: Molecules. 2015 Jun 3;20(6):10192–204. doi: 10.3390/molecules200610192 (PMC6272162; doi:10.3390/molecules200610192)

Supplementary Materials

Fluorescence spectra of 7.

Fluorescence spectrum (λ_{ex} = 350 nm)

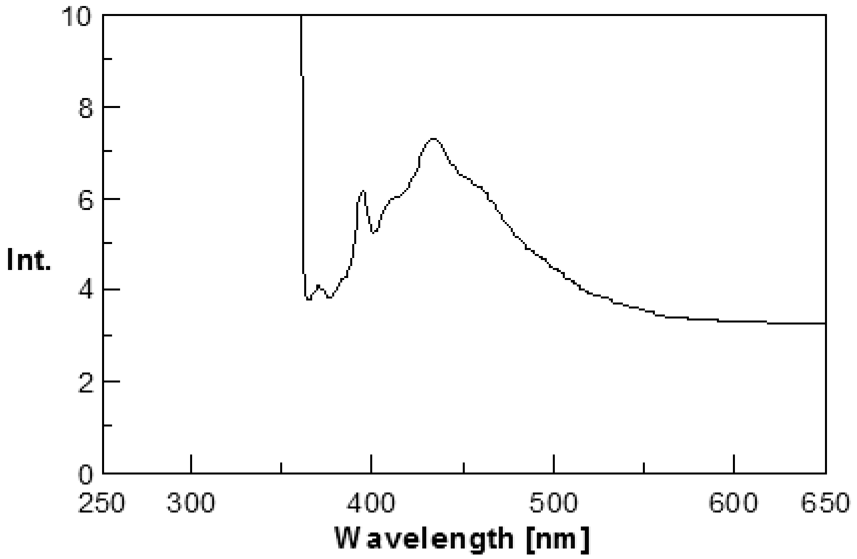

^{1}H- and ^{13}C-NMR spectra of 7.

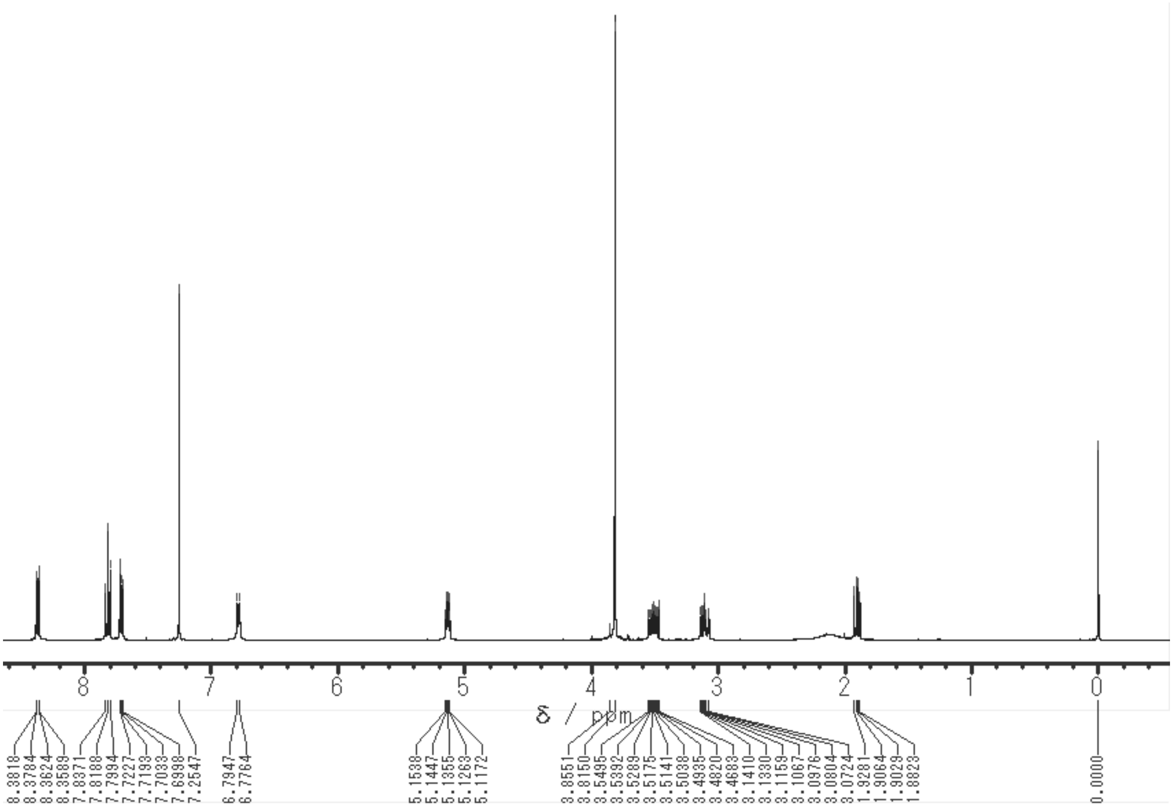

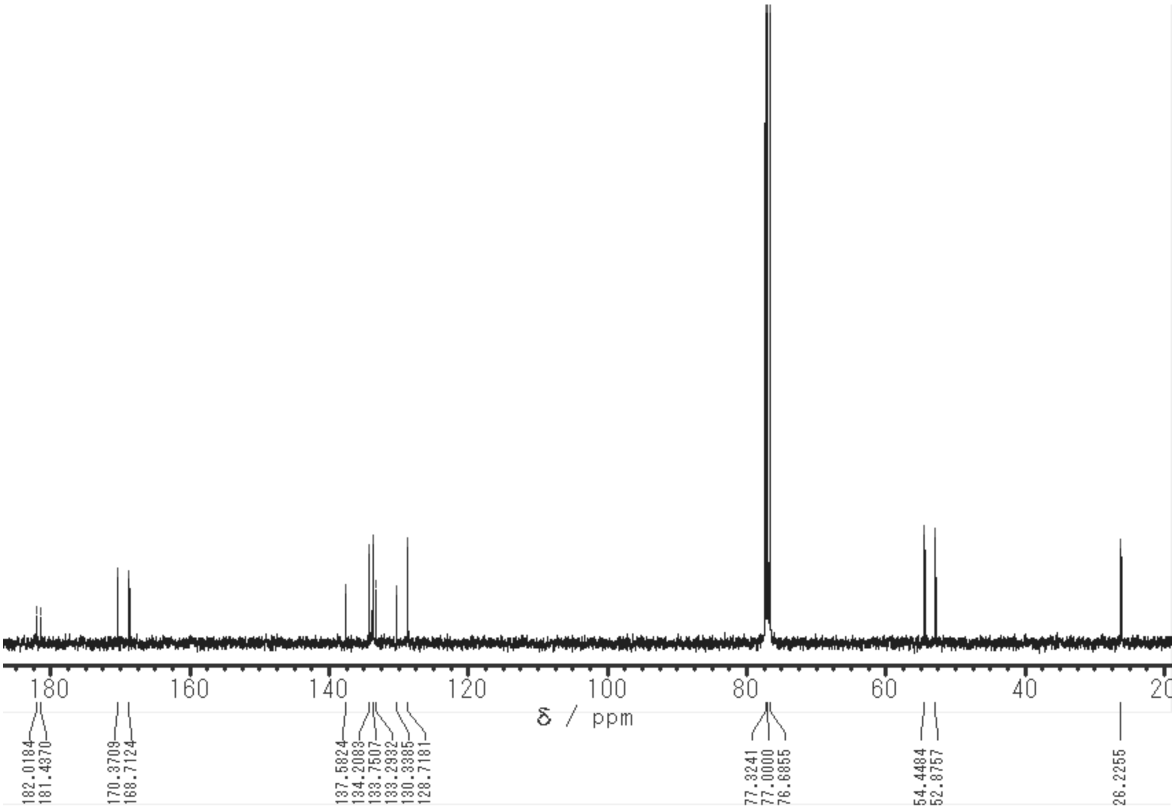

^{1}H- and ^{13}C-NMR spectra of 10.

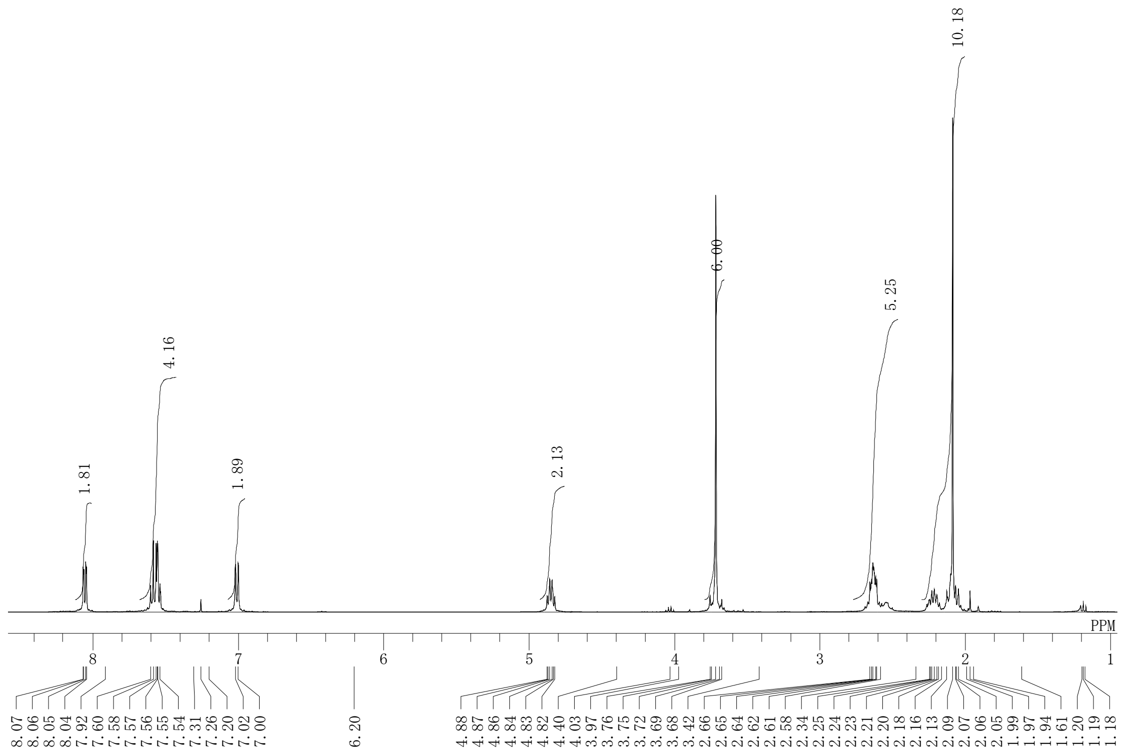

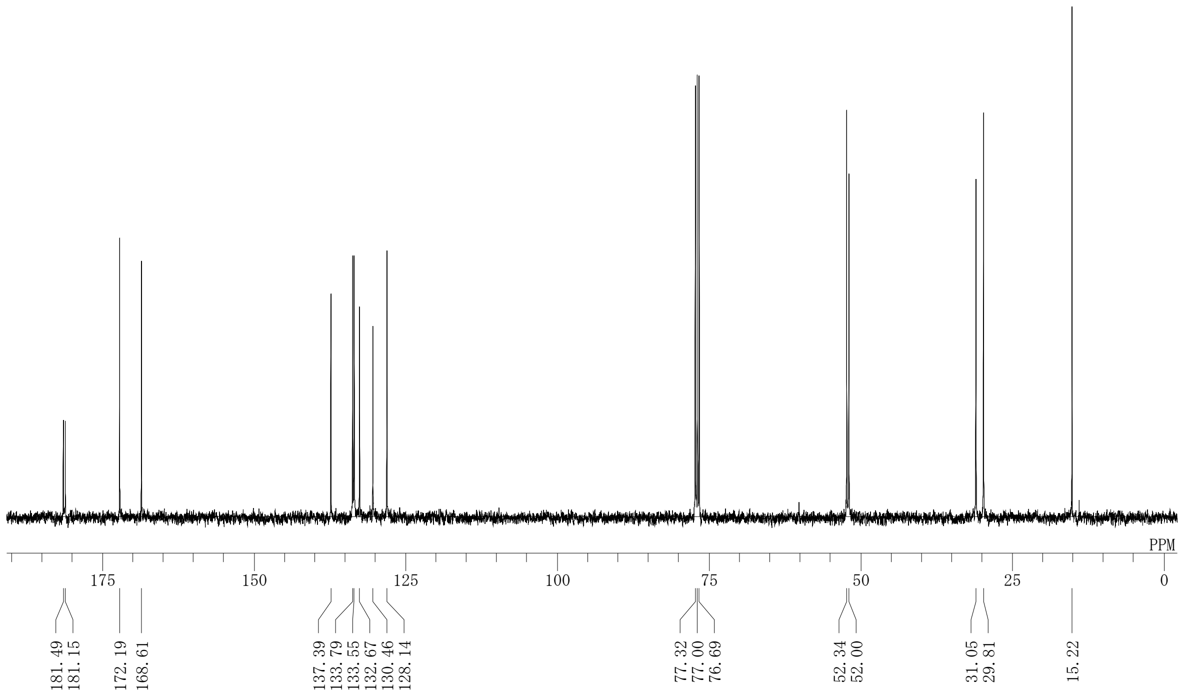

^{1}H- and ^{13}C-NMR spectra of 11.

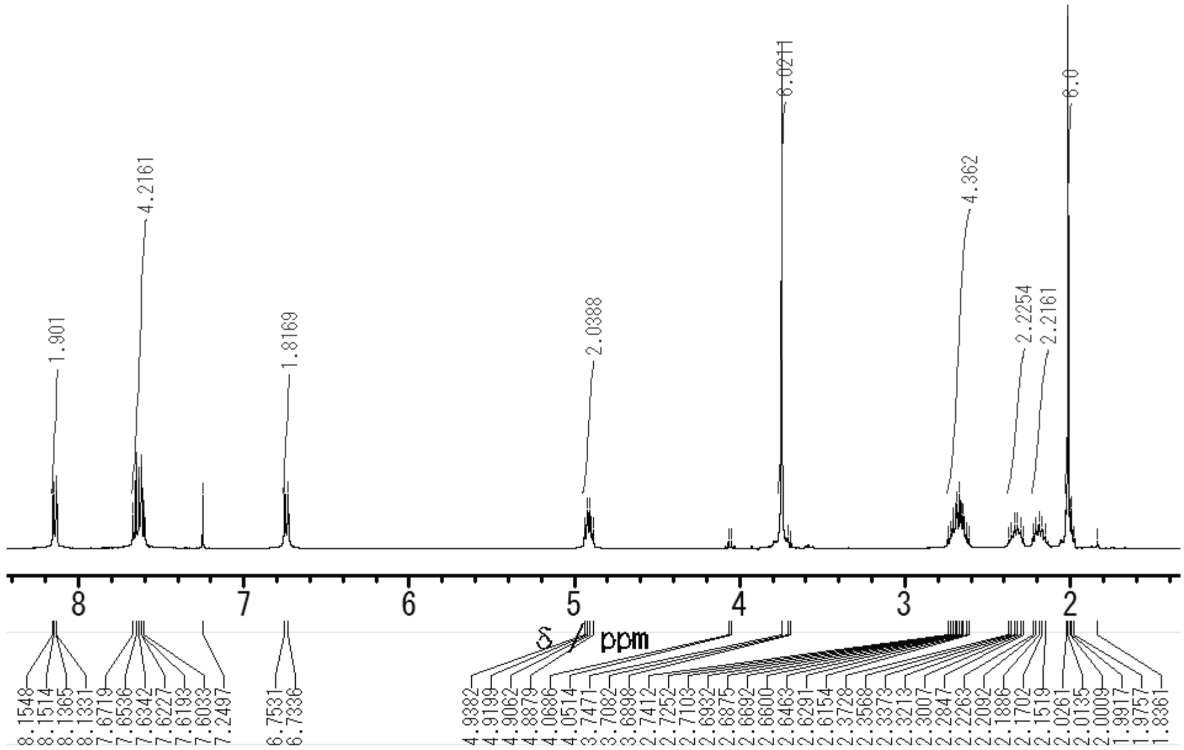

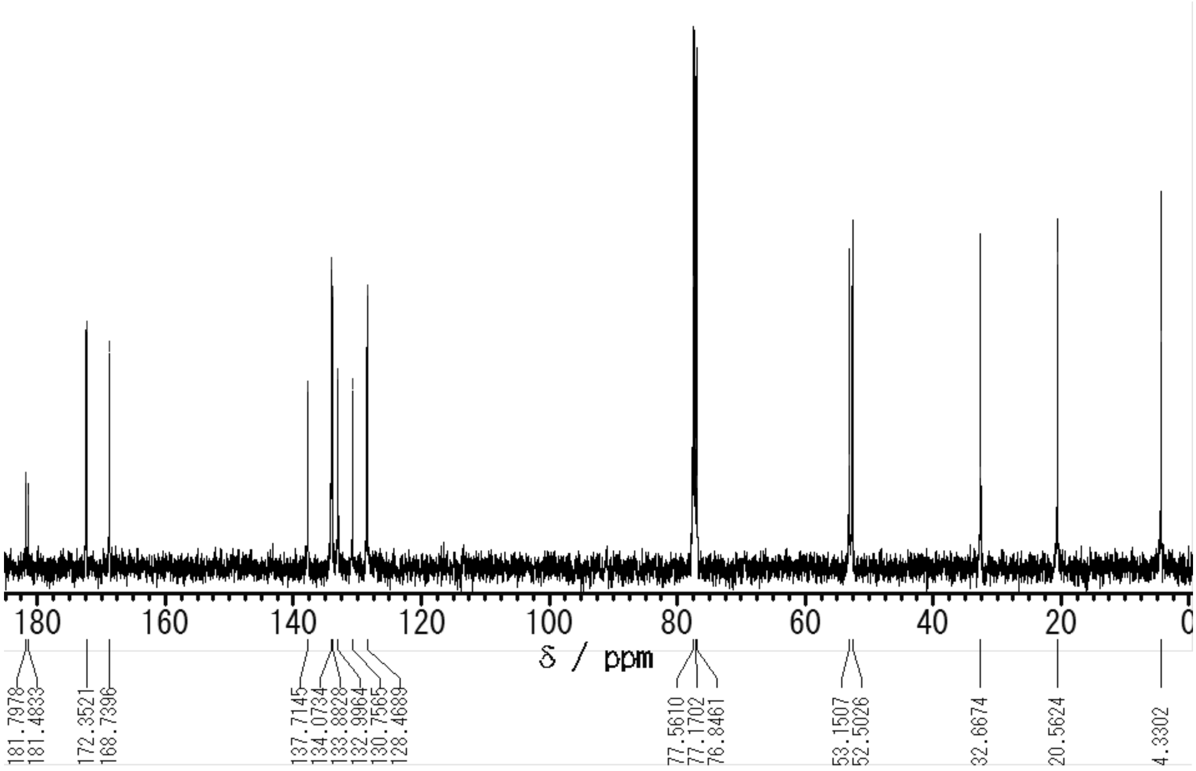

Supplement: Supplementary file 1 [file molecules-20-10192-s004.zip › molecules-80292-supplementary-1.pdf]
